# Robust circuitry-based scores of structural importance of human brain areas

**DOI:** 10.1371/journal.pone.0292613

**Published:** 2024-01-17

**Authors:** Dániel Hegedűs, Vince Grolmusz

**Affiliations:** 1 PIT Bioinformatics Group, Eötvös University, Budapest, Hungary; 2 Uratim Ltd., Budapest, Hungary; Medical University of Vienna: Medizinische Universitat Wien, AUSTRIA

## Abstract

We consider the 1015-vertex human consensus connectome computed from the diffusion MRI data of 1064 subjects. We define seven different orders on these 1015 graph vertices, where the orders depend on parameters derived from the brain circuitry, that is, from the properties of the edges (or connections) incident to the vertices ordered. We order the vertices according to their degree, the sum, the maximum, and the average of the fiber counts on the incident edges, and the sum, the maximum and the average length of the fibers in the incident edges. We analyze the similarities of these seven orders by the Spearman correlation coefficient and by their inversion numbers and have found that all of these seven orders have great similarities. In other words, if we interpret the orders as scoring of the importance of the vertices in the consensus connectome, then the scores of the vertices will be similar in all seven orderings. That is, important vertices of the human connectome typically have many neighbors connected with long and thick axonal fibers (where thickness is measured by fiber numbers), and their incident edges have high maximum and average values of length and fiber-number parameters, too. Therefore, these parameters may yield robust ways of deciding which vertices are more important in the anatomy of our brain circuitry than the others.

## Introduction

Identifying the most important nodes in large networks solely from their graph-theoretical properties was an important problem in the late 1990s, applied in scoring the web search engine hits. The most well-known solutions, the PageRank of Google [1] and the HITS algorithm of Kleinberg [2], fundamentally influenced the related areas.

Both the PageRank and the HITS algorithms score the nodes of a directed, unweighted graph, originally corresponded to the graph of the World Wide Web, but later, those algorithms were successfully applied for directed and undirected biological, social and chemical graphs, among other applications [3–6]. Other graph-theoretically inspired orders of importance were studied in biology in [7] in bipartite human disease-genes network and a more general setting, for undirected weighted graphs in [8].

Since all human activities are governed by the cooperation of the cells in our brain, the study of the connections of these cells has specific interest. Unfortunately, the connections of the 80 billion neurons of the human brain are not mapped yet and will not be mapped in the foreseeable future: to date, the only adult organism with completely mapped connections between its neurons (also called the connectome or braingraph) is the nematode *C. elegans*, having only 302 neurons [9]. The mapping was performed by electron-microscopy by staining of the axons of the nematode. Recently, after many years of concentrated efforts, the neuronal-level connectome of a part of the brain of the adult fruit fly *Drosophila melanogaster*, its central brain, is mapped and published [10]. Out of the 100,000 neurons of the fruit fly, the central brain contains around 25,000 neurons. More recently, the connectome of the Drosophila larva brain with 3016 neurons was published in [11]. The whole adult *Drosophila melanogaster* connectome is not published yet.

Instead of the neuronal-level connections, the imaging methods are capable today of mapping the human connectome on a much coarser scale than the level of the neurons. Due to the technical developments of magnetic resonance imaging (MRI) in the last fifteen years [12, 13], today we can map the macroscopic connections between 1000 anatomically identified brain areas. These developments have opened up a new area of brain science called “connectomics”. The connections between the brain areas are examined in the new field, and instead of comparing the volumes of brain areas between healthy or diseased, old and young, male or female subjects, it concentrates on a more central question: the connections between those areas.

Our research group has studied the mathematical properties of human connectomes by applying strict graph-theoretical methods and terms. We have used the public release imaging data sets of the Human Connectome Project [14] and prepared publicly available braingraphs from the imaging data, downloadable at the address https://braingraph.org in five different resolutions, i.e., with 86, 129, 234, 463 and 1015 nodes. [15–18]. The vertices of the braingraphs correspond to the anatomically identified areas of the cortical and sub-cortical gray matter, and two of the vertices are connected by an edge if the tractography phase [19, 20] of the processing identified axonal fibers between the areas, mapped to the vertices.

Using the exact methods and deep algorithms and approaches of graph theory (e.g., integer programming algorithms for computing exact solutions of NP-hard graph problems in connectomes, like minimum vertex cover or minimum bísection width), we have discovered numerous connectomical properties, related to the human sex differences [21–25], early brain development [16, 26–28], different lobal structures and organizations [29–31], and frequent edge sets in the whole brain or only those which are adjacent to the hippocampus [32–35].

In the present contribution, we consider an averaged consensus connectome computed from the imaging data of 1064 subjects, and we order the vertices of the consensus braingraph, intended to uncover their order of “importance” and compare the orders of vertices. Our main result is that orders generated from the

- degree of the nodes,
- sum of the number of fibers,
- maximum number of fibers,
- average number of fibers in the incident edges,
- sum of the fiber lengths,
- maximum fiber length,
- average fiber length in the incident edges

are similar to one another: their Spearman’s rank correlation is high, and their inversion numbers are low. Additionally, we also computed the Kendall rank correlation coefficient (Kendall’s *τ*, [36]), and gained similar results.

This result means that ordering by any of the seven parameters above produces similar orders of the nodes, where the “similar” word is explained in detail later in this work.

In other words, the result can be interpreted that the most important nodes in our braingraph statistically have numerous and long incident axonal fibers, with high maximum and averaged values either for the length or for the fiber numbers. That is, if a node is in front of others in one of the seven parameters above, then, typically, it will have high values in the remaining six parameters, too. Therefore, all of these seven orders are robust in comparison with the other six ones.

In what follows, we describe precisely our methods and results.

## Methods

### Graph construction

The primary data source of the present work is the 1200-subject public release of the Human Connectome Project [14]. The 3 Tesla diffusion magnetic resonance imaging data were processed with the help of the Connectome Mapper Tool Kit [19].

The braingraphs (or connectomes) applied in the present study were described, with their detailed construction in [17]. We have used the “1015 nodes set, 1064 brains, 1 000 000 streamlines, 10x repeated & averaged” link at the URL https://braingraph.org/download-pit-group-connectomes/.

While the detailed construction of the graphs is described in [17], which we have applied in numerous previous publications, we give here a very short summary of the construction: We have computed graphs on 1015 nodes, where each node corresponded to an anatomic area of the cortical- and sub-cortical gray matter. The parcellation tool FreeSurfer was applied here [20, 37, 38].

The details of the workflow we followed are described in [17]. Concisely, the axonal fibers were mapped by the MRtrix tractography software and repeated ten times for each subject. We connected two graph vertices, which corresponded to two gray matter areas, by an edge if, in all the 10 runs, axonal fibers were found running between the two areas. In this case, the maximum and the minimum number of fibers were deleted, and the remaining eight integer values were averaged and assigned to the edge as the fiber number weight. The length of the edge is also determined as the average length of the defining fibers. Consequently, all graph edges carry a positive weight (meaning the average of 8 fiber numbers) and a positive length (in millimeters).

Next, we constructed one single consensus graph on 1015 vertices from the 1064 individual graphs as follows. We have averaged the weight and the length for each edge, but we have followed different strategies. For averaging the weight, we added up the edge weight in graphs of all subject’s and divided the sum by 1064; if an edge was not present in a subject, then we counted it as an edge with (an artificial) weight of 0. In the case of computing the average length, the total summed lengths of the edges were divided by the number of the existing edges (for vertex pairs, which do not appear as an edge, 0 lengths were assigned).

Consequently, if #{*i*, *j*} denotes the number of appearance of edge {*i*, *j*}, and *s*_*i*,*j*,*k*_ and *h*_*i*,*j*,*k*_ denote in subject *k* the weight and the length of edge {*i*, *j*}, respectively, then

$$\begin{array}{c} s_{i,j}=\frac{1}{1064}\sum_{k=1}^{1064}s_{i,j,k} \end{array}$$ (1)

$$\begin{array}{c} h_{i,j}=\frac{1}{\#(i,j)}\sum_{k=1}^{1064}h_{i,j,k} \end{array}$$ (2)

We note that our earlier works [39, 40] also describe parameterizable consensus graphs by user-selectable parameters at the website of the Budapest Reference Connectome https://pitgroup.org/connectome. In contrast, the dataset of the present contribution is a static graph.

We need to add that in numerous previous articles we have analyzed the individual differences of the braingraphs (all these graphs are available from the repository https://braingraph.org) of the 1064 subjects in several viewpoints (see, e.g., [15, 16, 18, 21–35, 39, 40, 41]). Here we consider the consensus graph instead of the individual graphs.

### Ordering the nodes

Here we consider seven different orders on the set of the 1015 vertices of our consensus graph, with abbreviations:

- by the degree of the nodes (Degree);
- by the sum of the number of fibers in the incident edges (SUM-weight);
- by the maximum number of fibers in the incident edges (MAX-weight);
- by the average number of fibers in the incident edges (AVG-weight);
- by the sum of the fiber lengths in the incident edges (SUM-length);
- by the maximum fiber length in the incident edges (MAX-length);
- by the average fiber length in the incident edges (AVG-length).

The orders are defined by the decreasing values of these parameters.

The Degree describes the number of the incident edges, i.e., more important nodes are believed to be connected to many other nodes, so, consequently, their degree should be larger than the degree of the less important nodes.

SUM-weight is the weighted version of the Degree parameter. Here the fiber numbers of the incident edges are added up. Edges with higher fiber number or weight may connect more small gray matter areas than those with less weight. Consequently, we think that the SUM-weight parameter is more relevant in deciding the importance of a node than just the Degree.

MAX-weight—in a certain sense—is a simplification of the SUM-weight parameter. It describes only the weight of the largest-weight incident edge instead of adding up all the weights of the incident edges. Theoretically, it may happen that the ordering according to the MAX-weight differs a lot from the order defined by SUM-weight if, in many vertices, the incident edges have a small number of large and a large number of small weights. It turns out later that in the case of our graph, it is not true; the orders are similar.

AVG-weight: Clearly, for each vertex, the Degree times AVG-weight is the SUM-weight. Therefore, the AVG-weight-based vertex-order may differ strongly from both the Degree-order and the SUM-weight order. As we show, the AVG-weight based order is also similar to the Degree and to the SUM-weight order.

SUM-length is the length-weighted version of the Degree. The Degree and the SUM-length values may differ a lot if a node is adjacent to many other vertices by short edges or few other nodes but with very long edges. If an important node usually has numerous and long incident edges, then the orders by Degree and SUM-length would not differ a lot. We show that in the case of our consensus braingraph, this is the situation.

MAX-length can be large, while SUM-length is small, so the order according to these parameters can be different in numerous positions. We show that this is not the case in our graph.

AVG-length times the Degree is the SUM-length for each vertex. Therefore, the AVG-length-based order can strongly differ from both the Degree and the SUM-length based order. We show the opposite for the case of the consensus braingraph.

The seven orders are explicitly given in the S1 Table in the on-line supporting material.

In the following subsections, we introduce two tools for the analysis of the similarity of these orders: the Spearman correlation and the inversion numbers.

### Spearman’s rank correlation coefficient

The Spearman *ϱ* coefficient [42] is an ideal tool for comparing different orders on the same base set. In our case, the base set is the set of vertices, and the seven different orders are defined by the seven parameters Degree, SUM-weight, MAX-weight, AVG-weight, SUM-length, MAX-length, and AVG-length.

The *ϱ* coefficient gives information about the correlation of two attributes, using the two orderings by the two attributes of the elements. Two indices are associated with every element, telling what its index is in the given ordering. There is a simple equation that calculates the coefficient if the indices are unique, meaning that no two attributes are the same. (Luckily, this is true for the consensus braingraph.) If we calculate two attributes of *n* elements and *d*_*i*_ is the difference of the *i*-th element’s two indices, then:

$$\begin{array}{c} \varrho =1-\frac{6\cdot \sum_{i=1}^{n}d_{i}^{2}}{n^{3}-n} \end{array}$$ (3)

The value of Spearman’s correlation coefficient *ϱ* satisfies −1 ≤ *ϱ* ≤ 1, where *ϱ* = 1 means the perfect correlation and *ϱ* = −1 means the perfect opposition.

**Remark 1**. *The bounds for* *ϱ* *can be proven by using the cubic formula for the sum of the first* *n* *square numbers*: $\frac{n(n+1)(2n+1)}{6}$. *In the case of perfect opposition, we should calculate the sum of the first* $\frac{n}{2}$ *odd square numbers. This can easily be acquired from the sum of (not counted) even square numbers, as this is exactly four times the sum of the first that many square numbers*.

**Remark 2**. *The closer the coefficient* *ϱ* *is to* 0, *the less we can say about the predicted correlation. As* *n* *grows, the coefficients with smaller absolute values can also be significant. So the* *p-value is not only defined by* *ϱ*, *but it also depends on* *n*. *The acquired* *p-value is the probability of the correlation being that extreme under the assumption that the null hypothesis is true*.

### Inversion numbers

**Definition 1**. *In two given permutations of* *n* *elements, two different elements are* *in inversion with each other* *if their order is opposite in the permutations*.

**Definition 2**. *Two permutations’* *inversion number* *is the number of (not ordered) pairs of elements which are in inversion. An element’s* *inversion* *is the number of elements with which it is in inversion*.

**Lemma 3**. *The expected value of the inversion number of two permutations of length* *n* *is* $\frac{n(n-1)}{4}$.

*Proof*. Look at an arbitrary unordered {*i*, *j*} pair of elements. Because of symmetric reasoning, the expected value of the contribution of this pair to the inversion number is $\frac{1}{2}$. (As every permutation has a bijective pair which differs only in the (*i*, *j*) transposition. Transposition is the function that only swaps two elements in a permutation.) Since (n2)=n(n-1)2 unordered pair of elements exist in the set of *n* elements, by using the linearity of the expectation, we get that the expected value of the inversion number is the desired $\frac{1}{2}\cdot \frac{n(n-1)}{2}=\frac{n(n-1)}{4}$.

**Corollary 4**. *The expected value of the inversion of any element is* $\frac{n-1}{2}$.

*Proof*. The linearity of the expected value can be used again, but now for the result of Lemma 1. As there are *n* elements and each inversion is counted twice (for both elements of the pair), the expected value by elements is $\frac{n(n-1)}{4}\cdot \frac{2}{n}=\frac{n-1}{2}$.

*Alternative proof.* Make a bijection: Let the pair of a permutation be the opposite permutation. In every pair of permutations, every unordered pair of elements {*i*, *j*} has each of its two orderings in exactly one of the members of the permutation pair. So for every element *i*, there are *n* − 1 different *j* elements, and the expected value of their inversion one-by-one is $\frac{1}{2}$. So the expected value of the inversion of the arbitrary element *i* is $\frac{n-1}{2}$.

For any pair of the seven orders, we have computed the number of vertices in the graph with higher-than-expectation inversion-numbers. Let *N* = 1015, the number of vertices in the graph.

**Fig 1:**
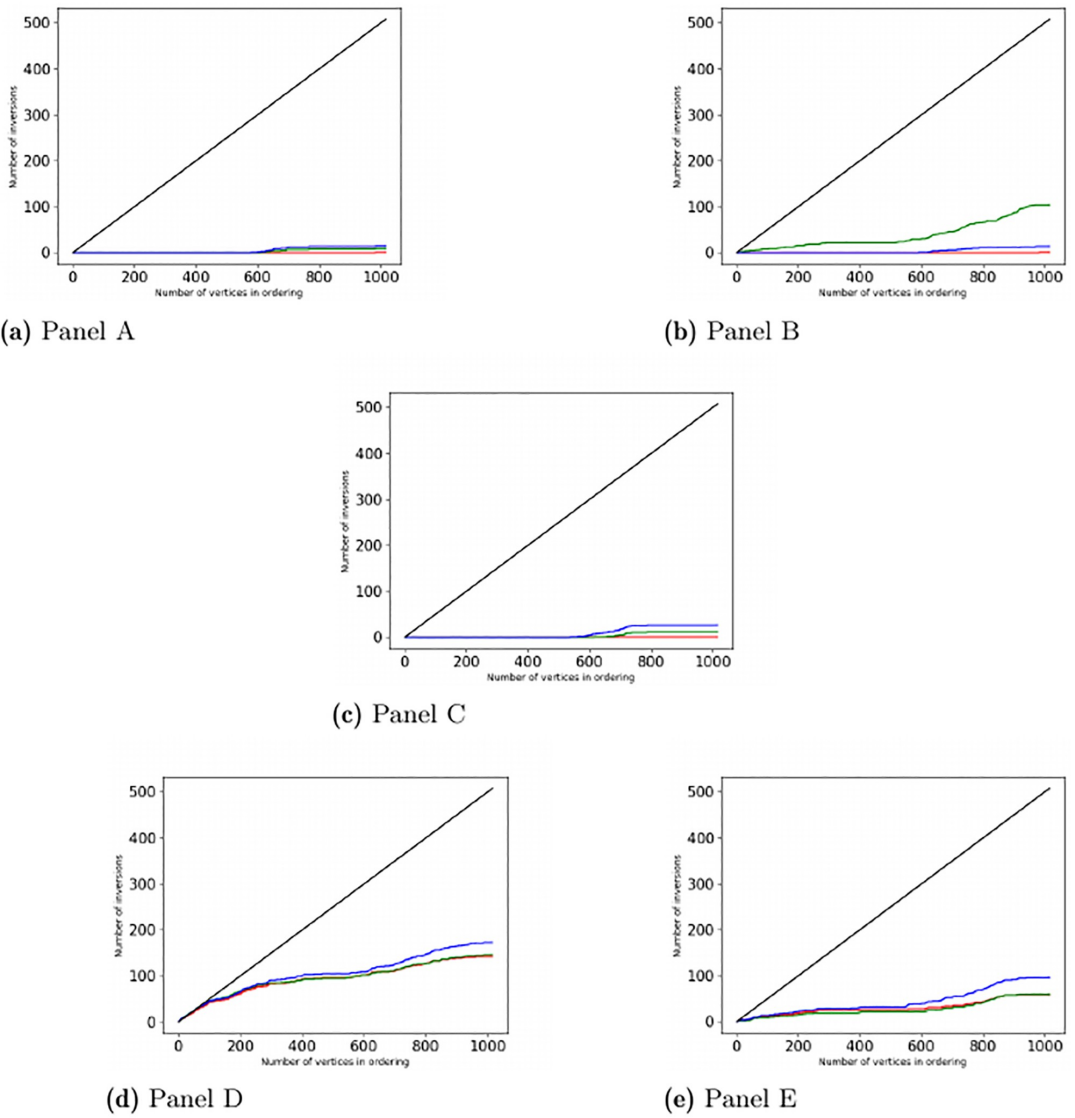
The number of vertices in the graph with higher-than-expectation inversion-numbers, visualized for distinct parameter-pairs in panels A, B, C, D and E. We denote the number of vertices by N, N = 1015. Axis x has labels from 0 through N, while axis y from 0 through (N-1)/2. On each panel, we compare three pairs of orders in a way that the first order is fixed, the second member of the pair changes. Namely, Panel A depicts the (Degree, SUM-Weight) comparison in red, (Degree, MAX-weight) comparison in green, (Degree, AVG-weight) comparison in blue color. Panel B contains the (Degree, SUM-length) comparison in red, the (Degree, MAX-length) comparison in green, the (Degree, AVG-length) comparison in blue color. Panel C demonstrates the (SUM-weight, SUM-length) comparison in red, the (SUM-weight, MAX-length) comparison in green, the (SUM-weight, AVG-length) comparison in blue. Panel D shows the (MAX-weight, SUM-length) comparison in red, the (MAX-weight, MAX-length) comparison in green, the (MAX-weight, AVG-length) comparison in blue. Panel E contains the (AVG-weight, SUM-length) comparison in red, the (AVG-weight, MAX-length) comparison in green, and the (AVG-weight, AVG-length) comparison in blue. On each panel, the vertices are ordered by their value according to the first (fixed) order. On each panel, point n on axis x, for any 0 < *n* ≤ *N* corresponds to the most important n vertices from the first ordering of the defined pair. The y coordinate of point n corresponds to the number with higher-than-expectation inversion values in the previously mentioned most important n pairs of vertices. On each panel the black line shows the *y* = *x*/2 expectation. It is easy to see on all panels that the higher-than-expectation inversion numbers appear in very few pairs, almost independently from the examined pairs of orderings.

For two orders, for example, the Degree and the SUM-weight, for each *n* = 1, 2, …, *N*, we first identified the *n* vertices with the highest ranks in the first order (i.e., the *n* vertices with the largest Degree). Next, we count the inversion numbers among these *n* vertices with respect to the second order. For each pairs of orders, we depicted these counts for each *n* = 1, 2, …, *N* on Fig 1 in the “Discussion and Results” section.

The scripts used for computations are enclosed as a supporting material.

## Discussion and results

### Spearman-correlations of different orderings

Table 1 shows that all seven orders are correlated strongly, with a very strong significance (*p* < 0^−41^).

**Table 1:** Spearman-correlations (*ϱ*) between the orders. We note that the weakest correlation 0.37, between the AVG-weight and MAX-length, is still far from value *ϱ* = 0, meaning no correlation. The significance analysis was done with null hypothesis that the orders are independent. For all pairs in the table, the *p* values are less than 10^−41^.

	SUM-weight	MAX-weight	AVG-weight	SUM-length	MAX-length	AVG-length
Degree	0.88	0.84	0.79	0.98	0.51	0.76
SUM-weight	1	0.97	0.98	0.86	0.42	0.63
MAX-weight	-	1	0.96	0.83	0.41	0.62
AVG-weight	-	-	1	0.77	0.37	0.54
SUM-length	-	-	-	1	0.58	0.86
MAX-length	-	-	-	-	1	0.70

### Three controls

In this section, we present three controls for our results in Table 1. Control 1 computes the Spearman-correlation and the related *p*-value for two unrelated orders, Control 2 computes the Kendall-*τ* coefficients [36], instead of the Spearman-coefficient, and in Control 3 we study the uniformity of the correlation: we sub-sample 100 connectomes randomly from the whole dataset three times, compute the three averaged braingraphs, and repeat the Spearman-correlation computation.

**Control 1**: For a simple control, we have computed the Spearman-correlation between two obviously unrelated orders. Namely, we have taken the ordinal numbers of the vertices assigned by the parcellation software and the AVG-weight-defined order. The ordinal numbers are assigned in the way that around the first 500 numbered vertices are situated in the left and the second 500 vertices in the right hemisphere of the brain, in the same order. For these two orders *ϱ* = 0.01 and *p* = 0.65, therefore, our results in Table 1 present a significant property of the human brain.

**Control 2**: Beside Spearman-correlation, we have computed the correlation coefficients [36] (also called Kendall’s *τ*) for the orders of Table 1, listed in Table 2. Similarly to Table 1, strong correlations between the orders with very low *p*-values are detected.

**Table 2:** Kendall-correlations (*τ*) between the orders. We note that the weakest correlation 0.25, between the AVG-weight and MAX-length, is still far from value *τ* = 0, meaning no correlation. The significance analysis was done with null hypothesis that the orders are independent. For all pairs in the table, the *p* value is less than 10^−32^.

	SUM-weight	MAX-weight	AVG-weight	SUM-length	MAX-length	AVG-length
Degree	0.72	0.67	0.62	0.89	0.36	0.56
SUM-weight	1	0.85	0.89	0.69	0.30	0.45
MAX-weight	-	1	0.84	0.66	0.29	0.44
AVG-weight	-	-	1	0.59	0.25	0.38
SUM-length	-	-	-	1	0.41	0.67
MAX-length	-	-	-	-	1	0.52

**Control 3**: In this control, we have verified that the results of Table 1 are not due to the averaging or “smoothing” on a very large set of connectomes. Here we have computed three average connectomes for three randomly chosen sub-samples of the connectomes of 100 subjects each, then re-computed the seven orders, and also re-computed the Spearman-correlations for each of these three subsamples, similarly as in Table 1. The range of the off-diagonal *ϱ* Spearman-coefficients and the maximum p-values are listed as follows: For sample 1: 0.61 ≤ *ϱ* ≤ 0.98 with *p*-values less than 10^−106^. For sample 2: 0.57 ≤ *ϱ* ≤ 0.98 with *p*-values less than 10^−88^. For sample 3: 0.62 ≤ *ϱ* ≤ 0.99 with *p*-values less than 10^−111^. These values show that the results in Table 1 are not due to the very large, smoothed dataset but rather the property of the braingraphs studied.

### Analysis of the order-similarity by inversion numbers

In the Methods section, we have defined the inversion numbers and listed some of their fundamental properties. Here, in Fig 1, we present a graphical evaluation of the inversion numbers between the pairs of the seven orders.

## Conclusions

We have analyzed the order of vertex-importance in the anatomically labeled consensus graph of the human brain, defined by circuitry-based parameters of the vertices: the degree (Degree), and the following parameters, computed on the incident edges for the vertex: Sum of fiber counts (SUM-weight), Max of fiber counts (MAX-weight), Average of fiber counts (AVG-weight), Sum of fiber lengths (SUM-length), Max of fiber lengths (MAX-length) and the Average of fiber lengths (AVG-length). For the analysis, we have used the Spearman correlation coefficient and the inversion numbers between the orders. We have found that the seven orders of vertex importance, defined by these seven circuitry-based parameters of the vertices, have a great similarity: i.e., the most important vertices—statistically—have many neighbors, connected with long and numerous fibers. We also have shown that orders defined by the maximum weight or length of the incident edges or the orders defined by the average weight or length of the incident edges do not differ very much from the orders defined by the sum of these parameters.

The results show the robustness of the orders by these seven parameters and also shows that vertex-importance in the human brain can be characterized by numerous parameters, but the list of the important vertices (or anatomical brain areas) will not be changed much.

## Supporting information

S1 Table This table lists the orderings of the 1015 vertices of our consensus braingraph by the examined weight parameters. The nodes are denoted by the cerebral areas, which they are corresponded to. The columns contain the orderings by different parameters, denoted in the column header.
(PDF)

S1 File Contains the scripts applied in the computation of our results. (PDF)
