# Supplementary material for: Robust circuitry-based scores of structural importance of human brain areas
Source: PLoS One. 2024 Jan 17;19(1):e0292613. doi: 10.1371/journal.pone.0292613 (PMC10793925; doi:10.1371/journal.pone.0292613)
Supplement: S1 Table — The nodes are denoted by the cerebral areas, which they are corresponded to. The columns contain the orderings by different parameters, denoted in the column header. (PDF) [file pone.0292613.s001.pdf]

# Supporting Material for the Robust Circuitry-Based Scores of Structural Importance of Human Brain Areas

Dániel Hegedűs[a], Vince Grolmusz[a,b,*]

[a] *PIT Bioinformatics Group, Eötvös University, H-1117 Budapest, Hungary*
[b] *Uratim Ltd., H-1118 Budapest, Hungary*

**Supporting Table 1**

We list in this table the orderings of the 1015 vertices of our consensus braingraph by the examined weight parameters. The nodes are denoted by the cerebral areas, which they are corresponded to. The columns contain the orderings by different parameters, denoted in the column header.

*Corresponding author
*Email addresses:* hegedus@pitgroup.org (Dániel Hegedűs), grolmusz@pitgroup.org (Vince Grolmusz)

| | Degree | Sum of fiber counts (SUM-weight) | Max of fiber counts (MAX-weight) | Average of fiber counts (AVG-weight) | Sum of fiber lengths (SUM-length) | Max of fiber lengths (MAX-length) | Average of fiber lengths (AVG-length) |
|---|---|---|---|---|---|---|---|
| 1 | Left-Thalamus-Proper | Left-Caudate | Left-Caudate | Left-Caudate | Left-Thalamus-Proper | lh.superiorfrontal_11 | rh.rostralmiddlefrontal_14 |
| 2 | Right-Thalamus-Proper | Left-Thalamus-Proper | Left-Putamen | Left-Thalamus-Proper | Right-Thalamus-Proper | lh.superiorparietal_16 | lh.rostralmiddlefrontal_5 |
| 3 | Right-Caudate | Right-Caudate | Right-Caudate | Right-Caudate | Right-Caudate | lh.lateralorbitofrontal_1 | lh.lateralorbitofrontal_5 |
| 4 | Left-Caudate | Right-Thalamus-Proper | Right-Putamen | Left-Putamen | Left-Caudate | lh.superiorparietal_4 | rh.superiorfrontal_2 |
| 5 | Right-Putamen | Left-Putamen | Right-Thalamus-Proper | Right-Putamen | Right-Putamen | lh.medialorbitofrontal_5 | lh.precuneus_20 |
| 6 | Left-Putamen | Right-Putamen | Left-Thalamus-Proper | Right-Thalamus-Proper | Left-Putamen | lh.rostralmiddlefrontal_1 | lh.superiorfrontal_32 |
| 7 | Right-Hippocampus | Left-Hippocampus | lh.posteriorcingulate_6 | Left-Hippocampus | Right-Hippocampus | lh.precuneus_17 | lh.rostralmiddlefrontal_23 |
| 8 | Left-Hippocampus | Right-Hippocampus | Left-Pallidum | Right-Hippocampus | lh.superiorparietal_25 | lh.rostralmiddlefrontal_3 | rh.insula_14 |
| 9 | lh.superiorparietal_25 | rh.caudalmiddlefrontal_11 | Left-Hippocampus | rh.caudalmiddlefrontal_11 | Right-Pallidum | lh.superiorparietal_19 | rh.rostralmiddlefrontal_2 |
| 10 | rh.insula_16 | lh.superiorparietal_25 | Right-Hippocampus | lh.lateraloccipital_3 | Left-Hippocampus | rh.lateralorbitofrontal_14 | lh.rostralmiddlefrontal_19 |
| 11 | rh.posteriorcingulate_1 | rh.superiorparietal_13 | rh.posteriorcingulate_7 | rh.lateraloccipital_3 | Left-Pallidum | rh.superiorparietal_14 | rh.caudalmiddlefrontal_6 |
| 12 | rh.supramarginal_17 | lh.caudalmiddlefrontal_13 | lh.caudalmiddlefrontal_13 | lh.pericalcarine_6 | rh.posteriorcingulate_1 | lh.superiorfrontal_8 | rh.superiorfrontal_11 |
| 13 | lh.insula_5 | rh.supramarginal_17 | Right-Pallidum | rh.lingual_6 | rh.caudalmiddlefrontal_11 | lh.precuneus_4 | rh.rostralmiddlefrontal_24 |
| 14 | Left-Pallidum | lh.superiorparietal_27 | rh.caudalmiddlefrontal_11 | rh.superiorparietal_13 | lh.posteriorcingulate_5 | rh.superiorparietal_25 | rh.rostralmiddlefrontal_10 |
| 15 | Right-Pallidum | lh.isthmuscingulate_2 | lh.posteriorcingulate_4 | rh.fusiform_2 | lh.caudalmiddlefrontal_13 | rh.rostralmiddlefrontal_24 | lh.superiorfrontal_29 |
| 16 | rh.posteriorcingulate_5 | lh.precentral_6 | rh.posteriorcingulate_1 | lh.caudalmiddlefrontal_13 | rh.posteriorcingulate_5 | rh.fusiform_9 | rh.caudalmiddlefrontal_10 |
| 17 | lh.posteriorcingulate_5 | lh.insula_5 | lh.posteriorcingulate_2 | lh.superiorparietal_25 | rh.caudalanteriorcingulate_3 | lh.medialorbitofrontal_4 | rh.rostralmiddlefrontal_21 |
| 18 | lh.posteriorcingulate_6 | lh.supramarginal_16 | rh.isthmuscingulate_3 | lh.lateraloccipital_18 | lh.superiorparietal_27 | lh.superiorparietal_10 | rh.superiorparietal_22 |
| 19 | rh.posteriorcingulate_6 | rh.lingual_6 | rh.isthmuscingulate_5 | lh.isthmuscingulate_2 | rh.insula_10 | lh.precuneus_13 | lh.superiorfrontal_23 |
| 20 | lh.superiorparietal_27 | lh.posteriorcingulate_6 | lh.supramarginal_16 | lh.superiorparietal_27 | rh.insula_16 | rh.lateraloccipital_17 | rh.caudalmiddlefrontal_9 |
| 21 | rh.caudalanteriorcingulate_3 | rh.isthmuscingulate_5 | lh.caudalanteriorcingulate_1 | rh.lateraloccipital_5 | lh.posteriorcingulate_6 | lh.precuneus_20 | lh.parsopercularis_7 |
| 22 | lh.isthmuscingulate_4 | rh.superiorparietal_24 | lh.superiorparietal_27 | rh.supramarginal_17 | rh.superiorparietal_24 | lh.rostralmiddlefrontal_7 | lh.superiorfrontal_44 |
| 23 | rh.superiorparietal_13 | rh.insula_13 | lh.isthmuscingulate_2 | rh.lateraloccipital_15 | rh.superiorparietal_22 | lh.lateralorbitofrontal_13 | rh.caudalmiddlefrontal_1 |
| 24 | rh.superiorparietal_24 | rh.lateraloccipital_3 | lh.isthmuscingulate_4 | lh.precuneus_15 | rh.caudalmiddlefrontal_10 | lh.precuneus_19 | lh.parsopercularis_6 |
| 25 | rh.caudalmiddlefrontal_11 | lh.caudalmiddlefrontal_5 | lh.posteriorcingulate_1 | lh.precentral_6 | rh.posteriorcingulate_6 | rh.rostralmiddlefrontal_12 | lh.caudalmiddlefrontal_9 |
| 26 | rh.insula_13 | lh.precuneus_15 | rh.posteriorcingulate_2 | rh.pericalcarine_1 | rh.superiorparietal_13 | rh.superiorparietal_10 | rh.insula_10 |
| 27 | lh.supramarginal_16 | rh.inferiorparietal_20 | rh.posteriorcingulate_5 | rh.superiorfrontal_28 | lh.posteriorcingulate_8 | rh.pericalcarine_2 | rh.insula_8 |
| 28 | rh.inferiorparietal_18 | lh.pericalcarine_6 | lh.precentral_6 | lh.pericalcarine_4 | rh.supramarginal_17 | rh.precuneus_16 | rh.rostralmiddlefrontal_18 |
| 29 | lh.caudalmiddlefrontal_13 | Right-Pallidum | lh.posteriorcingulate_5 | lh.inferiortemporal_7 | rh.insula_11 | rh.parsorbitalis_3 | rh.rostralmiddlefrontal_23 |
| 30 | lh.posteriorcingulate_8 | rh.supramarginal_11 | lh.superiorparietal_25 | lh.supramarginal_16 | rh.inferiorparietal_13 | rh.superiorparietal_15 | lh.precuneus_4 |
| 31 | lh.posteriorcingulate_2 | rh.precentral_7 | lh.caudalanteriorcingulate_3 | lh.fusiform_17 | rh.superiorparietal_14 | rh.lingual_4 | rh.caudalmiddlefrontal_7 |
| 32 | rh.inferiorparietal_13 | Left-Pallidum | lh.posteriorcingulate_8 | lh.lingual_2 | lh.superiorparietal_16 | rh.rostralmiddlefrontal_3 | lh.superiorparietal_19 |
| 33 | lh.posteriorcingulate_1 | lh.precuneus_12 | rh.superiorparietal_13 | rh.precentral_7 | lh.posteriorcingulate_2 | rh.rostralmiddlefrontal_22 | rh.superiorparietal_25 |
| 34 | rh.supramarginal_19 | lh.lateraloccipital_3 | rh.posteriorcingulate_6 | lh.insula_5 | lh.supramarginal_16 | rh.fusiform_2 | Right-Pallidum |
| 35 | lh.precuneus_12 | lh.inferiorparietal_21 | rh.posteriorcingulate_9 | rh.isthmuscingulate_5 | lh.isthmuscingulate_4 | rh.superiorparietal_9 | rh.parsopercularis_9 |
| 36 | rh.isthmuscingulate_5 | rh.supramarginal_19 | lh.pericalcarine_6 | rh.inferiorparietal_20 | lh.caudalanteriorcingulate_2 | lh.lateralorbitofrontal_5 | Right-Thalamus-Proper |
| 37 | lh.inferiorparietal_20 | rh.lateraloccipital_5 | lh.lateraloccipital_18 | rh.fusiform_17 | lh.precuneus_12 | lh.medialorbitofrontal_7 | rh.insula_9 |
| 38 | lh.isthmuscingulate_2 | rh.bankssts_1 | Brain-Stem | lh.caudalmiddlefrontal_5 | rh.caudalmiddlefrontal_6 | rh.rostralmiddlefrontal_2 | rh.caudalmiddlefrontal_11 |
| 39 | rh.isthmuscingulate_1 | lh.isthmuscingulate_3 | lh.caudalanteriorcingulate_2 | rh.supramarginal_11 | lh.caudalmiddlefrontal_5 | rh.superiorparietal_11 | lh.caudalmiddlefrontal_4 |
| 40 | rh.insula_10 | rh.insula_11 | lh.isthmuscingulate_3 | lh.lingual_7 | lh.insula_5 | rh.rostralmiddlefrontal_6 | lh.superiorfrontal_21 |
| 41 | lh.inferiorparietal_21 | rh.precentral_20 | rh.insula_16 | rh.superiorparietal_24 | lh.posteriorcingulate_1 | rh.superiorparietal_22 | rh.superiorfrontal_14 |
| 42 | rh.insula_11 | rh.supramarginal_5 | rh.caudalanteriorcingulate_3 | lh.posteriorcingulate_6 | lh.inferiorparietal_20 | lh.superiorfrontal_21 | rh.rostralmiddlefrontal_22 |
| 43 | lh.caudalanteriorcingulate_2 | rh.precentral_19 | lh.insula_5 | rh.pericalcarine_2 | rh.isthmuscingulate_5 | lh.precuneus_6 | lh.rostralmiddlefrontal_4 |
| 44 | lh.insula_16 | lh.lateraloccipital_18 | lh.inferiorparietal_21 | rh.precentral_20 | rh.parsopercularis_9 | rh.parsorbitalis_1 | lh.rostralmiddlefrontal_26 |

| | | | | | | | |
|---|---|---|---|---|---|---|---|
| 45 | lh.posteriorcingulate_9 | rh.inferiorparietal_18 | rh.caudalanteriorcingulate_2 | lh.lateraloccipital_23 | rh.caudalmiddlefrontal_1 | rh.caudalmiddlefrontal_6 | lh.rostralmiddlefrontal_3 |
| 46 | lh.precentral_6 | rh.posteriorcingulate_1 | rh.isthmuscingulate_1 | lh.lingual_14 | rh.insula_13 | lh.rostralmiddlefrontal_14 | lh.superiorparietal_10 |
| 47 | rh.precuneus_20 | lh.insula_2 | rh.parsopercularis_2 | rh.lateraloccipital_7 | rh.inferiorparietal_18 | rh.rostralmiddlefrontal_14 | rh.parsopercularis_6 |
| 48 | lh.isthmuscingulate_6 | rh.posteriorcingulate_6 | rh.posteriorcingulate_3 | lh.isthmuscingulate_3 | rh.insula_1 | rh.lingual_5 | rh.inferiorparietal_16 |
| 49 | lh.superiorparietal_16 | lh.inferiorparietal_20 | lh.isthmuscingulate_6 | rh.insula_13 | rh.rostralmiddlefrontal_21 | lh.precuneus_7 | rh.superiorparietal_15 |
| 50 | lh.isthmuscingulate_4 | rh.caudalmiddlefrontal_10 | rh.caudalanteriorcingulate_5 | rh.lateraloccipital_23 | lh.insula_15 | Left-Hippocampus | Left-Thalamus-Proper |
| 51 | rh.superiorparietal_22 | rh.inferiorparietal_13 | rh.supramarginal_11 | rh.precentral_19 | rh.precuneus_20 | rh.rostralmiddlefrontal_5 | lh.rostralmiddlefrontal_9 |
| 52 | rh.bankssts_1 | rh.precuneus_20 | rh.precuneus_6 | rh.supramarginal_5 | rh.inferiorparietal_21 | lh.precuneus_11 | lh.superiorfrontal_8 |
| 53 | rh.isthmuscingulate_3 | lh.posteriorcingulate_5 | rh.supramarginal_19 | lh.superiorfrontal_22 | rh.inferiorparietal_8 | rh.rostralmiddlefrontal_26 | lh.superiorfrontal_11 |
| 54 | lh.caudalmiddlefrontal_5 | rh.insula_16 | rh.lateraloccipital_3 | rh.inferiortemporal_13 | lh.superiorparietal_13 | rh.superiorparietal_13 | lh.caudalmiddlefrontal_3 |
| 55 | lh.superiorparietal_13 | lh.inferiortemporal_7 | rh.lingual_6 | rh.lingual_4 | lh.caudalanteriorcingulate_3 | lh.lateralorbitofrontal_7 | rh.inferiorparietal_1 |
| 56 | rh.caudalmiddlefrontal_10 | lh.isthmuscingulate_4 | lh.superiorfrontal_44 | rh.pericalcarine_7 | rh.parsopercularis_2 | lh.precuneus_5 | rh.rostralmiddlefrontal_3 |
| 57 | rh.superiorparietal_14 | lh.precentral_15 | rh.caudalmiddlefrontal_10 | rh.inferiorparietal_22 | lh.precentral_15 | lh.lateralorbitofrontal_8 | rh.rostralmiddlefrontal_13 |
| 58 | lh.supramarginal_15 | rh.superiorfrontal_28 | rh.inferiorparietal_20 | rh.bankssts_1 | lh.inferiorparietal_12 | lh.precuneus_21 | rh.rostralmiddlefrontal_20 |
| 59 | rh.inferiorparietal_21 | rh.posteriorcingulate_7 | rh.posteriorcingulate_8 | lh.pericalcarine_5 | lh.parsopercularis_6 | lh.rostralmiddlefrontal_10 | lh.caudalmiddlefrontal_8 |
| 60 | rh.inferiorparietal_12 | lh.superiortemporal_24 | lh.insula_2 | lh.precuneus_12 | rh.inferiorparietal_17 | lh.precuneus_22 | lh.caudalmiddlefrontal_13 |
| 61 | lh.caudalanteriorcingulate_3 | lh.lingual_2 | lh.insula_16 | lh.inferiorparietal_21 | rh.superiorparietal_9 | lh.lateralorbitofrontal_2 | rh.inferiorparietal_3 |
| 62 | lh.inferiorparietal_12 | lh.inferiorparietal_18 | rh.isthmuscingulate_6 | rh.precuneus_3 | lh.precuneus_4 | rh.lateraloccipital_3 | lh.caudalmiddlefrontal_6 |
| 63 | lh.precuneus_6 | lh.superiorparietal_13 | rh.insula_10 | lh.insula_2 | lh.isthmuscingulate_2 | rh.inferiorparietal_15 | rh.superiorfrontal_17 |
| 64 | lh.precentral_15 | lh.bankssts_4 | rh.paracentral_8 | rh.insula_11 | rh.parsopercularis_6 | rh.superiorparietal_25 | lh.paracentral_7 |
| 65 | lh.supramarginal_2 | rh.insula_10 | lh.caudalmiddlefrontal_9 | rh.supramarginal_19 | rh.inferiorparietal_12 | lh.lateralorbitofrontal_3 | lh.superiorparietal_24 |
| 66 | rh.precuneus_10 | rh.inferiorparietal_22 | rh.insula_13 | rh.bankssts_2 | lh.posteriorcingulate_9 | lh.superiorfrontal_10 | lh.superiorfrontal_41 |
| 67 | rh.superiorparietal_9 | lh.supramarginal_15 | lh.caudalanteriorcingulate_4 | lh.lateraloccipital_5 | lh.precuneus_6 | rh.precuneus_20 | lh.parsopercularis_1 |
| 68 | rh.inferiorparietal_8 | rh.fusiform_2 | rh.lateraloccipital_5 | lh.superiorfrontal_2 | rh.precuneus_10 | lh.rostralanteriorcingulate_3 | lh.rostralmiddlefrontal_22 |
| 69 | rh.caudalanteriorcingulate_5 | rh.caudalmiddlefrontal_6 | lh.postcentral_31 | rh.superiorfrontal_22 | rh.caudalanteriorcingulate_5 | rh.superiorfrontal_23 | lh.parstriangularis_2 |
| 70 | rh.inferiorparietal_20 | rh.isthmuscingulate_3 | rh.precentral_20 | lh.inferiorparietal_18 | rh.supramarginal_19 | lh.rostralmiddlefrontal_25 | Left-Pallidum |
| 71 | lh.isthmuscingulate_5 | lh.insula_14 | rh.superiorparietal_24 | Right-Pallidum | lh.caudalmiddlefrontal_9 | lh.superiorparietal_12 | lh.lateralorbitofrontal_1 |
| 72 | lh.superiortemporal_24 | rh.precuneus_6 | rh.caudalmiddlefrontal_1 | lh.lingual_8 | rh.inferiorparietal_14 | rh.superiorfrontal_2 | rh.precentral_29 |
| 73 | lh.insula_2 | rh.precuneus_3 | lh.inferiorparietal_18 | lh.lingual_1 | lh.rostralmiddlefrontal_4 | rh.rostralmiddlefrontal_21 | rh.insula_1 |
| 74 | rh.parsopercularis_2 | lh.caudalmiddlefrontal_10 | lh.superiorfrontal_27 | lh.lingual_6 | rh.posteriorcingulate_7 | rh.rostralmiddlefrontal_27 | rh.superiorparietal_14 |
| 75 | lh.insula_15 | lh.precuneus_6 | lh.caudalmiddlefrontal_5 | lh.bankssts_2 | rh.isthmuscingulate_1 | rh.precuneus_10 | lh.superiorfrontal_14 |
| 76 | rh.insula_1 | lh.lingual_7 | rh.caudalmiddlefrontal_6 | lh.bankssts_4 | rh.rostralmiddlefrontal_10 | lh.parstriangularis_2 | lh.insula_15 |
| 77 | rh.posteriorcingulate_7 | lh.caudalanteriorcingulate_3 | rh.superiorfrontal_38 | lh.postcentral_7 | rh.precentral_12 | lh.superiorparietal_1 | rh.paracentral_2 |
| 78 | rh.posteriorcingulate_2 | lh.superiorfrontal_44 | rh.superiorfrontal_28 | rh.caudalmiddlefrontal_10 | rh.isthmuscingulate_4 | lh.superiorparietal_24 | lh.parsopercularis_9 |
| 79 | rh.superiorparietal_11 | rh.parsopercularis_2 | lh.precentral_15 | lh.superiorfrontal_20 | lh.inferiorparietal_21 | lh.rostralmiddlefrontal_8 | rh.insula_7 |
| 80 | rh.supramarginal_11 | lh.lateraloccipital_23 | rh.supramarginal_17 | lh.superiorfrontal_44 | rh.rostralmiddlefrontal_18 | rh.precuneus_2 | lh.rostralmiddlefrontal_10 |
| 81 | rh.inferiorparietal_17 | rh.precentral_12 | rh.inferiorparietal_12 | Left-Pallidum | lh.caudalmiddlefrontal_3 | rh.isthmuscingulate_4 | lh.caudalmiddlefrontal_1 |
| 82 | rh.precuneus_6 | lh.postcentral_7 | rh.pericalcarine_1 | lh.inferiorparietal_20 | lh.precuneus_13 | rh.lateralorbitofrontal_15 | lh.superiorparietal_16 |
| 83 | lh.inferiorparietal_2 | rh.paracentral_8 | lh.rostralmiddlefrontal_4 | rh.posteriorcingulate_7 | lh.insula_4 | rh.rostralmiddlefrontal_9 | lh.paracentral_4 |
| 84 | rh.supramarginal_5 | lh.postcentral_31 | rh.bankssts_1 | rh.superiorfrontal_11 | lh.precentral_6 | lh.rostralmiddlefrontal_26 | lh.caudalmiddlefrontal_11 |
| 85 | rh.inferiorparietal_14 | rh.pericalcarine_1 | lh.precentral_25 | lh.caudalmiddlefrontal_10 | rh.rostralmiddlefrontal_24 | rh.precuneus_1 | rh.parsopercularis_5 |
| 86 | rh.insula_6 | rh.postcentral_24 | rh.precuneus_4 | lh.precentral_15 | lh.supramarginal_15 | lh.rostralanteriorcingulate_4 | lh.superiorparietal_25 |
| 87 | lh.insula_14 | lh.posteriorcingulate_1 | rh.inferiorparietal_13 | lh.lateraloccipital_7 | rh.isthmuscingulate_3 | rh.superiorparietal_18 | rh.posteriorcingulate_1 |
| 88 | rh.parsopercularis_9 | rh.precuneus_10 | lh.supramarginal_15 | rh.inferiorparietal_18 | lh.isthmuscingulate_6 | lh.superiorfrontal_32 | rh.inferiorparietal_17 |
| 89 | rh.precentral_19 | lh.bankssts_2 | rh.insula_11 | lh.superiorfrontal_27 | lh.insula_14 | lh.rostralmiddlefrontal_5 | rh.insula_11 |
| 90 | lh.precuneus_13 | rh.superiorfrontal_11 | rh.superiorfrontal_18 | lh.superiortemporal_24 | lh.superiorfrontal_44 | rh.superiorfrontal_14 | rh.superiorfrontal_19 |

| | | | | | | | |
|---|---|---|---|---|---|---|---|
| 91 | rh.precentral_12 | lh.inferiorparietal_2 | lh.lateraloccipital_3 | rh.precuneus_20 | lh.posteriorcingulate_4 | rh.superiorparietal_2 | rh.parsopercularis_2 |
| 92 | rh.supramarginal_10 | lh.superiorfrontal_22 | lh.lingual_1 | rh.caudalmiddlefrontal_6 | rh.inferiorparietal_20 | rh.rostralmiddlefrontal_1 | lh.rostralmiddlefrontal_21 |
| 93 | rh.caudalmiddlefrontal_1 | rh.bankssts_2 | lh.precuneus_15 | lh.precentral_21 | lh.caudalmiddlefrontal_4 | rh.paracentral_10 | lh.insula_4 |
| 94 | rh.caudalmiddlefrontal_6 | lh.precentral_23 | lh.bankssts_4 | rh.lateraloccipital_17 | rh.superiorparietal_11 | lh.superiorfrontal_14 | rh.inferiorparietal_2 |
| 95 | lh.posteriorcingulate_4 | lh.posteriorcingulate_8 | rh.caudalanteriorcingulate_4 | rh.lingual_3 | rh.posteriorcingulate_2 | rh.lateralorbitofrontal_1 | lh.posteriorcingulate_5 |
| 96 | lh.isthmuscingulate_7 | rh.inferiorparietal_14 | lh.inferiorparietal_17 | rh.lateraloccipital_22 | lh.caudalmiddlefrontal_10 | lh.precuneus_9 | rh.inferiorparietal_19 |
| 97 | lh.isthmuscingulate_3 | rh.precuneus_1 | lh.pericalcarine_4 | rh.postcentral_24 | rh.bankssts_1 | lh.rostralmiddlefrontal_23 | lh.precentral_23 |
| 98 | lh.insula_8 | lh.superiorfrontal_27 | rh.rostralmiddlefrontal_10 | rh.lingual_10 | lh.inferiorparietal_2 | lh.rostralmiddlefrontal_18 | lh.caudalmiddlefrontal_7 |
| 99 | rh.parsopercularis_6 | lh.posteriorcingulate_4 | rh.inferiorparietal_18 | rh.inferiorparietal_13 | rh.supramarginal_10 | rh.lateralorbitofrontal_3 | rh.superiorfrontal_27 |
| 100 | rh.posteriorcingulate_3 | lh.caudalmiddlefrontal_3 | lh.superiortemporal_24 | lh.insula_14 | rh.caudalmiddlefrontal_5 | lh.superiorparietal_21 | Right-Putamen |
| 101 | lh.postcentral_31 | lh.supramarginal_2 | lh.lingual_14 | rh.superiorparietal_3 | rh.insula_8 | rh.precuneus_9 | rh.rostralmiddlefrontal_27 |
| 102 | rh.rostralmiddlefrontal_21 | rh.isthmuscingulate_6 | lh.posteriorcingulate_9 | rh.posteriorcingulate_6 | lh.insula_16 | lh.superiorparietal_20 | lh.caudalmiddlefrontal_10 |
| 103 | lh.insula_4 | rh.inferiortemporal_13 | rh.lateraloccipital_17 | lh.superiorparietal_13 | lh.superiorparietal_24 | rh.precuneus_3 | lh.parsopercularis_8 |
| 104 | lh.bankssts_4 | rh.precuneus_4 | rh.fusiform_2 | rh.precuneus_6 | lh.isthmuscingulate_5 | rh.superiorfrontal_11 | lh.caudalmiddlefrontal_5 |
| 105 | rh.posteriorcingulate_9 | rh.superiorparietal_22 | lh.insula_4 | lh.superiorfrontal_45 | rh.precuneus_16 | lh.precentral_33 | rh.precentral_13 |
| 106 | rh.supramarginal_6 | rh.precentral_22 | lh.lateraloccipital_7 | rh.paracentral_8 | rh.superiorfrontal_11 | lh.rostralmiddlefrontal_19 | rh.caudalmiddlefrontal_5 |
| 107 | lh.parsopercularis_6 | rh.inferiorparietal_12 | rh.superiorfrontal_11 | lh.lateraloccipital_17 | rh.precentral_20 | rh.medialorbitofrontal_10 | lh.lateralorbitofrontal_13 |
| 108 | lh.precuneus_11 | lh.precentral_21 | lh.precuneus_9 | lh.inferiorparietal_16 | rh.insula_6 | rh.precuneus_19 | rh.parstriangularis_8 |
| 109 | rh.precentral_20 | rh.superiorparietal_11 | lh.lingual_8 | lh.supramarginal_15 | lh.precentral_23 | lh.superiorfrontal_29 | rh.inferiorparietal_8 |
| 110 | rh.paracentral_8 | rh.precentral_15 | rh.insula_1 | rh.posteriorcingulate_1 | lh.parsopercularis_8 | lh.superiorfrontal_35 | lh.precuneus_17 |
| 111 | rh.precuneus_1 | rh.superiorfrontal_22 | lh.superiorfrontal_28 | rh.superiorfrontal_25 | rh.inferiorparietal_2 | lh.postcentral_18 | rh.precentral_12 |
| 112 | rh.precuneus_4 | lh.superiorfrontal_2 | lh.superiorfrontal_2 | rh.superiorfrontal_24 | rh.precuneus_6 | lh.paracentral_3 | rh.inferiorparietal_14 |
| 113 | lh.rostralmiddlefrontal_4 | rh.fusiform_17 | lh.insula_8 | lh.postcentral_31 | lh.rostralmiddlefrontal_19 | lh.rostralanteriorcingulate_2 | rh.paracentral_3 |
| 114 | lh.supramarginal_3 | lh.caudalmiddlefrontal_9 | rh.isthmuscingulate_4 | rh.precentral_12 | rh.inferiorparietal_19 | rh.superiorparietal_24 | lh.rostralmiddlefrontal_15 |
| 115 | lh.superiorparietal_11 | rh.posteriorcingulate_9 | lh.inferiorparietal_12 | lh.superiorfrontal_43 | rh.supramarginal_11 | rh.rostralmiddlefrontal_4 | rh.rostralmiddlefrontal_4 |
| 116 | lh.precuneus_4 | lh.precuneus_9 | rh.precentral_19 | lh.precentral_23 | lh.posteriorcingulate_7 | lh.superiorparietal_22 | lh.superiorfrontal_42 |
| 117 | lh.caudalmiddlefrontal_3 | lh.caudalmiddlefrontal_4 | rh.inferiorparietal_22 | lh.pericalcarine_2 | lh.caudalanteriorcingulate_4 | rh.lingual_6 | rh.superiorparietal_10 |
| 118 | lh.superiorparietal_20 | lh.precuneus_13 | rh.precuneus_3 | rh.parsopercularis_2 | lh.superiorparietal_11 | lh.isthmuscingulate_3 | rh.precentral_33 |
| 119 | lh.caudalmiddlefrontal_10 | rh.lateraloccipital_15 | lh.precuneus_12 | lh.superiorparietal_1 | lh.supramarginal_2 | lh.superiorparietal_13 | rh.precentral_14 |
| 120 | lh.inferiorparietal_18 | rh.insula_6 | lh.lateraloccipital_23 | rh.insula_10 | lh.insula_2 | lh.rostralmiddlefrontal_21 | lh.caudalanteriorcingulate_2 |
| 121 | rh.caudalmiddlefrontal_5 | rh.caudalmiddlefrontal_1 | lh.postcentral_7 | rh.isthmuscingulate_3 | lh.isthmuscingulate_3 | lh.rostralanteriorcingulate_1 | rh.caudalanteriorcingulate_3 |
| 122 | lh.caudalmiddlefrontal_9 | rh.postcentral_26 | lh.insula_14 | lh.precuneus_6 | rh.superiorparietal_10 | rh.rostralmiddlefrontal_23 | lh.rostralanteriorcingulate_3 |
| 123 | lh.caudalanteriorcingulate_4 | rh.posteriorcingulate_5 | lh.caudalmiddlefrontal_3 | rh.pericalcarine_5 | rh.precuneus_4 | lh.parstriangularis_6 | lh.lateralorbitofrontal_8 |
| 124 | rh.precuneus_16 | rh.lateraloccipital_7 | lh.lingual_7 | lh.lateraloccipital_21 | rh.posteriorcingulate_3 | rh.precuneus_6 | rh.precuneus_16 |
| 125 | rh.precentral_7 | rh.superiorfrontal_31 | lh.inferiortemporal_7 | rh.superiorfrontal_31 | lh.isthmuscingulate_7 | lh.inferiorparietal_20 | rh.superiorparietal_24 |
| 126 | lh.precentral_4 | lh.fusiform_17 | rh.superiorfrontal_25 | lh.isthmuscingulate_4 | lh.insula_8 | rh.inferiorparietal_13 | lh.precentral_15 |
| 127 | rh.isthmuscingulate_6 | lh.superiorfrontal_20 | lh.lingual_2 | rh.precentral_15 | lh.rostralmiddlefrontal_13 | rh.superiorparietal_5 | lh.posteriorcingulate_8 |
| 128 | rh.supramarginal_12 | lh.insula_8 | lh.superiorfrontal_45 | lh.posteriorcingulate_5 | rh.supramarginal_5 | rh.inferiorparietal_24 | lh.precentral_33 |
| 129 | rh.precuneus_9 | lh.rostralmiddlefrontal_4 | rh.pericalcarine_7 | rh.superiorfrontal_41 | lh.rostralmiddlefrontal_26 | lh.paracentral_4 | lh.superiorparietal_27 |
| 130 | lh.posteriorcingulate_7 | rh.superiorfrontal_24 | rh.lateraloccipital_23 | lh.caudalanteriorcingulate_3 | lh.precuneus_20 | lh.insula_4 | rh.rostralmiddlefrontal_19 |
| 131 | rh.rostralmiddlefrontal_18 | lh.precentral_16 | rh.inferiorparietal_8 | lh.precentral_16 | rh.supramarginal_8 | lh.rostralanteriorcingulate_5 | rh.posteriorcingulate_7 |
| 132 | rh.precentral_9 | lh.isthmuscingulate_5 | lh.rostralmiddlefrontal_9 | rh.inferiorparietal_23 | lh.superiorparietal_20 | lh.inferiorparietal_17 | lh.precentral_19 |
| 133 | lh.precuneus_9 | rh.supramarginal_12 | lh.superiorfrontal_37 | rh.postcentral_26 | lh.precentral_4 | rh.caudalmiddlefrontal_8 | lh.precuneus_13 |
| 134 | rh.posteriorcingulate_8 | lh.precentral_4 | lh.superiorfrontal_43 | rh.isthmuscingulate_6 | lh.parsopercularis_7 | lh.superiorparietal_11 | lh.superiorparietal_15 |
| 135 | rh.precentral_22 | lh.inferiorparietal_12 | lh.inferiorparietal_6 | rh.lingual_12 | rh.insula_7 | lh.lingual_2 | rh.inferiorparietal_21 |
| 136 | rh.supramarginal_8 | lh.inferiorparietal_17 | lh.caudalanteriorcingulate_5 | rh.precentral_22 | rh.precentral_19 | Right-Hippocampus | lh.caudalanteriorcingulate_3 |

| | | | | | | | |
|---|---|---|---|---|---|---|---|
| 137 | rh.inferiorparietal_9 | lh.precuneus_4 | rh.caudalanteriorcingulate_1 | lh.caudalmiddlefrontal_3 | rh.inferiorparietal_15 | lh.superiorparietal_15 | lh.superiorfrontal_10 |
| 138 | rh.parsopercularis_8 | Brain-Stem | lh.pericalcarine_5 | rh.precuneus_1 | rh.precentral_9 | lh.inferiorparietal_21 | rh.rostralmiddlefrontal_13 |
| 139 | lh.precentral_23 | lh.lateraloccipital_5 | lh.precuneus_11 | lh.fusiform_11 | rh.parstriangularis_8 | rh.isthmuscingulate_2 | rh.parsopercularis_4 |
| 140 | rh.rostralmiddlefrontal_10 | lh.superiorparietal_20 | rh.superiorfrontal_13 | lh.lateraloccipital_11 | lh.precuneus_11 | lh.inferiorparietal_15 | rh.superiorparietal_18 |
| 141 | rh.inferiorparietal_2 | rh.caudalmiddlefrontal_5 | rh.precentral_7 | rh.middletemporal_12 | rh.precentral_7 | lh.paracentral_10 | rh.superiorfrontal_34 |
| 142 | lh.caudalmiddlefrontal_4 | rh.inferiorparietal_21 | rh.lateraloccipital_6 | rh.lateraloccipital_6 | lh.superiortemporal_24 | lh.parstriangularis_4 | rh.superiorparietal_13 |
| 143 | rh.postcentral_24 | rh.middletemporal_12 | rh.precuneus_19 | lh.inferiorparietal_2 | rh.posteriorcingulate_8 | lh.precuneus_10 | rh.superiorparietal_9 |
| 144 | rh.postcentral_30 | lh.inferiorparietal_16 | rh.inferiorparietal_23 | rh.precuneus_4 | lh.rostralmiddlefrontal_9 | lh.precuneus_8 | Right-Caudate |
| 145 | rh.superiorparietal_10 | rh.isthmuscingulate_1 | lh.bankssts_2 | lh.superiorfrontal_28 | lh.inferiorparietal_18 | lh.superiorfrontal_42 | lh.paracentral_3 |
| 146 | rh.inferiorparietal_19 | lh.supramarginal_1 | lh.rostralmiddlefrontal_19 | rh.insula_16 | rh.precuneus_9 | lh.superiorparietal_2 | lh.superiorfrontal_35 |
| 147 | rh.inferiorparietal_15 | lh.precuneus_11 | rh.insula_5 | lh.posteriorcingulate_4 | lh.parstriangularis_2 | rh.superiorfrontal_8 | Right-Amygdala |
| 148 | lh.superiorparietal_24 | rh.superiorparietal_14 | rh.pericalcarine_2 | rh.precuneus_10 | rh.inferiorparietal_16 | rh.inferiorparietal_12 | lh.posteriorcingulate_4 |
| 149 | lh.precuneus_8 | lh.inferiorparietal_15 | rh.lateraloccipital_7 | lh.inferiorparietal_17 | rh.rostralmiddlefrontal_22 | rh.lingual_15 | rh.parsopercularis_7 |
| 150 | lh.superiorfrontal_44 | rh.parsopercularis_9 | rh.inferiorparietal_21 | lh.caudalmiddlefrontal_4 | rh.superiorparietal_18 | rh.rostralmiddlefrontal_7 | rh.inferiorparietal_26 |
| 151 | rh.rostralmiddlefrontal_24 | rh.inferiorparietal_23 | rh.precentral_22 | rh.inferiorparietal_14 | lh.supramarginal_3 | lh.superiorparietal_6 | lh.caudalmiddlefrontal_2 |
| 152 | rh.insula_5 | lh.isthmuscingulate_6 | rh.superiorfrontal_24 | Brain-Stem | lh.caudalmiddlefrontal_1 | rh.bankssts_1 | rh.posteriorcingulate_5 |
| 153 | rh.insula_4 | lh.pericalcarine_4 | lh.inferiorparietal_20 | lh.superiorfrontal_38 | rh.paracentral_8 | lh.inferiorparietal_12 | lh.insula_10 |
| 154 | lh.superiorparietal_6 | lh.superiorparietal_24 | lh.inferiorparietal_16 | rh.superiorfrontal_42 | rh.supramarginal_6 | rh.caudalmiddlefrontal_11 | lh.superiorfrontal_31 |
| 155 | lh.precuneus_15 | rh.precuneus_16 | lh.supramarginal_17 | lh.precuneus_9 | lh.precuneus_9 | rh.lateraloccipital_19 | rh.parstriangularis_3 |
| 156 | rh.inferiorparietal_22 | lh.caudalanteriorcingulate_1 | rh.superiorparietal_14 | lh.caudalmiddlefrontal_9 | rh.inferiorparietal_3 | rh.rostralanteriorcingulate_1 | rh.superiorfrontal_26 |
| 157 | lh.precentral_25 | rh.superiorparietal_10 | rh.precuneus_20 | lh.superiorfrontal_1 | rh.insula_4 | lh.precuneus_12 | rh.caudalanteriorcingulate_5 |
| 158 | lh.paracentral_6 | lh.insula_15 | lh.superiorparietal_13 | lh.superiorfrontal_31 | rh.precuneus_1 | rh.parsopercularis_6 | rh.caudalmiddlefrontal_4 |
| 159 | lh.supramarginal_1 | rh.superiorfrontal_42 | lh.insula_15 | rh.posteriorcingulate_9 | lh.postcentral_31 | lh.posteriorcingulate_7 | lh.superiorparietal_13 |
| 160 | rh.insula_8 | lh.precentral_25 | lh.caudalmiddlefrontal_1 | lh.inferiorparietal_6 | lh.superiorfrontal_29 | rh.lateraloccipital_5 | lh.inferiorparietal_12 |
| 161 | lh.paracentral_5 | rh.precentral_23 | lh.superiorfrontal_31 | rh.superiorfrontal_27 | rh.superiorparietal_25 | rh.caudalmiddlefrontal_10 | lh.superiorfrontal_25 |
| 162 | lh.precentral_8 | rh.paracentral_7 | lh.fusiform_17 | lh.supramarginal_2 | rh.posteriorcingulate_9 | rh.lateraloccipital_4 | lh.paracentral_1 |
| 163 | rh.precentral_15 | lh.posteriorcingulate_2 | rh.superiorfrontal_31 | lh.precuneus_18 | lh.precuneus_8 | lh.superiorfrontal_44 | lh.posteriorcingulate_7 |
| 164 | lh.postcentral_7 | lh.superiorfrontal_43 | rh.bankssts_2 | lh.posteriorcingulate_1 | lh.caudalanteriorcingulate_1 | lh.superiorparietal_14 | lh.posteriorcingulate_6 |
| 165 | lh.inferiorparietal_15 | rh.rostralmiddlefrontal_10 | lh.rostralmiddlefrontal_13 | rh.lateraloccipital_8 | lh.bankssts_4 | lh.parsopercularis_1 | rh.posteriorcingulate_6 |
| 166 | rh.parstriangularis_8 | rh.supramarginal_6 | lh.lateraloccipital_21 | rh.superiorfrontal_2 | rh.supramarginal_12 | lh.parahippocampal_1 | rh.parsopercularis_1 |
| 167 | rh.paracentral_7 | rh.isthmuscingulate_4 | lh.lingual_15 | lh.supramarginal_1 | rh.rostralmiddlefrontal_20 | lh.precentral_17 | rh.paracentral_5 |
| 168 | rh.postcentral_29 | rh.inferiorparietal_8 | rh.supramarginal_5 | lh.superiorfrontal_37 | rh.isthmuscingulate_6 | lh.superiorparietal_27 | Left-Putamen |
| 169 | rh.superiorparietal_18 | lh.posteriorcingulate_9 | rh.lateraloccipital_15 | rh.superiorparietal_11 | rh.inferiorparietal_9 | rh.precuneus_5 | rh.precuneus_10 |
| 170 | lh.supramarginal_21 | rh.lateraloccipital_23 | rh.lingual_10 | rh.fusiform_14 | lh.paracentral_6 | Right-Accumbens-area | rh.precuneus_20 |
| 171 | rh.caudalanteriorcingulate_1 | lh.fusiform_11 | rh.superiorfrontal_41 | lh.inferiorparietal_15 | lh.rostralmiddlefrontal_22 | rh.superiorfrontal_19 | lh.rostralanteriorcingulate_2 |
| 172 | rh.precuneus_3 | rh.caudalanteriorcingulate_5 | rh.superiorfrontal_17 | lh.caudalanteriorcingulate_1 | lh.supramarginal_21 | rh.caudalmiddlefrontal_9 | rh.superiorfrontal_22 |
| 173 | rh.precuneus_19 | lh.caudalanteriorcingulate_2 | rh.parsopercularis_6 | rh.supramarginal_12 | rh.postcentral_24 | rh.inferiorparietal_1 | lh.caudalanteriorcingulate_1 |
| 174 | rh.insula_7 | lh.insula_16 | rh.superiorparietal_3 | rh.superiorparietal_22 | lh.paracentral_5 | lh.postcentral_14 | rh.precentral_20 |
| 175 | lh.paracentral_11 | rh.superiorfrontal_25 | rh.inferiorparietal_24 | rh.precentral_23 | lh.inferiorparietal_22 | lh.superiorfrontal_9 | lh.superiorparietal_21 |
| 176 | lh.superiorparietal_2 | rh.postcentral_29 | rh.insula_6 | lh.superiorfrontal_42 | lh.precentral_8 | rh.lateralorbitofrontal_12 | lh.inferiorparietal_7 |
| 177 | lh.inferiorparietal_17 | lh.superiorparietal_1 | rh.precuneus_1 | rh.superiorfrontal_38 | lh.superiorparietal_6 | rh.isthmuscingulate_5 | lh.precuneus_12 |
| 178 | lh.precuneus_22 | rh.superiorfrontal_41 | rh.lingual_5 | lh.precentral_4 | lh.caudalmiddlefrontal_8 | rh.paracentral_8 | lh.parsopercularis_4 |
| 179 | rh.superiorfrontal_11 | lh.precuneus_18 | lh.superiorparietal_1 | rh.caudalmiddlefrontal_1 | rh.caudalanteriorcingulate_4 | rh.rostralmiddlefrontal_17 | lh.superiorparietal_12 |
| 180 | rh.supramarginal_13 | rh.posteriorcingulate_2 | lh.pericalcarine_3 | lh.precuneus_13 | rh.rostralmiddlefrontal_19 | rh.superiorparietal_29 | rh.superiorfrontal_31 |
| 181 | lh.rostralmiddlefrontal_13 | rh.lateraloccipital_22 | rh.inferiortemporal_13 | rh.inferiorparietal_12 | rh.precentral_33 | rh.superiorfrontal_4 | rh.inferiorparietal_11 |
| 182 | lh.caudalanteriorcingulate_1 | lh.parsopercularis_6 | rh.lingual_4 | lh.rostralmiddlefrontal_4 | rh.caudalanteriorcingulate_1 | rh.supramarginal_11 | rh.caudalanteriorcingulate_4 |

| | | | | | | | |
|---|---|---|---|---|---|---|---|
| 183 | rh.inferiorparietal_25 | lh.superiorparietal_2 | rh.middletemporal_12 | rh.superiorparietal_8 | lh.inferiorparietal_5 | rh.lateraloccipital_10 | lh.inferiorparietal_20 |
| 184 | rh.precuneus_5 | lh.lingual_6 | rh.precuneus_10 | rh.superiorfrontal_18 | rh.caudalmiddlefrontal_9 | rh.superiorfrontal_37 | rh.inferiorparietal_13 |
| 185 | lh.inferiorparietal_22 | rh.pericalcarine_2 | rh.supramarginal_10 | rh.lateraloccipital_9 | rh.inferiorparietal_1 | rh.rostralmiddlefrontal_13 | lh.superiorfrontal_16 |
| 186 | rh.precentral_3 | lh.pericalcarine_5 | rh.postcentral_24 | lh.superiorfrontal_29 | lh.precuneus_22 | lh.isthmuscingulate_5 | lh.caudalanteriorcingulate_4 |
| 187 | rh.superiortemporal_14 | lh.lateraloccipital_21 | lh.supramarginal_1 | rh.fusiform_9 | rh.precentral_22 | lh.superiorfrontal_24 | rh.insula_2 |
| 188 | lh.rostralmiddlefrontal_26 | rh.caudalanteriorcingulate_3 | lh.superiorfrontal_22 | rh.insula_6 | lh.parsopercularis_4 | rh.supramarginal_10 | lh.insula_14 |
| 189 | lh.inferiorparietal_5 | lh.superiorfrontal_31 | lh.paracentral_5 | lh.precuneus_4 | rh.precuneus_19 | rh.insula_2 | lh.precentral_12 |
| 190 | rh.precentral_23 | rh.superiorparietal_9 | rh.lingual_12 | lh.insula_8 | rh.inferiorparietal_22 | rh.insula_10 | lh.superiorfrontal_3 |
| 191 | rh.postcentral_26 | rh.superiorfrontal_27 | lh.inferiorparietal_2 | rh.paracentral_7 | rh.superiorfrontal_31 | rh.superiorfrontal_3 | rh.isthmuscingulate_5 |
| 192 | lh.rostralmiddlefrontal_19 | lh.superiorfrontal_38 | rh.rostralmiddlefrontal_21 | lh.fusiform_7 | lh.superiorparietal_10 | rh.inferiorparietal_8 | lh.precuneus_6 |
| 193 | rh.caudalanteriorcingulate_4 | rh.inferiorparietal_15 | rh.precentral_12 | rh.caudalmiddlefrontal_5 | rh.postcentral_29 | lh.insula_5 | rh.paracentral_4 |
| 194 | lh.parstriangularis_2 | lh.supramarginal_3 | rh.precentral_23 | lh.superiorparietal_20 | rh.inferiorparietal_25 | lh.postcentral_23 | lh.supramarginal_18 |
| 195 | lh.caudalmiddlefrontal_1 | lh.lingual_14 | lh.posteriorcingulate_3 | lh.posteriorcingulate_8 | rh.precentral_3 | rh.rostralmiddlefrontal_19 | rh.inferiorparietal_15 |
| 196 | rh.transversetemporal_3 | lh.superiorfrontal_29 | rh.rostralmiddlefrontal_24 | lh.precentral_25 | rh.insula_15 | rh.inferiorparietal_7 | rh.inferiorparietal_12 |
| 197 | lh.superiorfrontal_27 | rh.postcentral_30 | rh.supramarginal_6 | lh.paracentral_3 | rh.precentral_15 | rh.precuneus_4 | lh.inferiorparietal_22 |
| 198 | rh.insula_15 | lh.paracentral_5 | lh.superiorfrontal_29 | lh.superiorparietal_24 | lh.caudalmiddlefrontal_6 | rh.lateraloccipital_15 | lh.rostralmiddlefrontal_14 |
| 199 | rh.isthmuscingulate_2 | rh.precuneus_19 | lh.lateraloccipital_5 | lh.lateraloccipital_4 | rh.precentral_29 | lh.parsopercularis_7 | lh.superiorparietal_4 |
| 200 | lh.rostralmiddlefrontal_9 | rh.precentral_9 | rh.precentral_15 | lh.paracentral_4 | lh.posteriorcingulate_3 | lh.parahippocampal_5 | lh.posteriorcingulate_2 |
| 201 | lh.posteriorcingulate_3 | lh.lateraloccipital_7 | lh.isthmuscingulate_5 | rh.inferiorparietal_24 | rh.superiorparietal_15 | rh.rostralanteriorcingulate_3 | rh.supramarginal_8 |
| 202 | lh.isthmuscingulate_1 | rh.superiorparietal_3 | lh.caudalmiddlefrontal_10 | rh.superiorparietal_10 | lh.inferiorparietal_15 | rh.superiorparietal_12 | lh.precentral_17 |
| 203 | rh.postcentral_21 | rh.lingual_3 | lh.fusiform_11 | lh.lingual_10 | lh.postcentral_7 | lh.lateralorbitofrontal_10 | rh.superiorfrontal_35 |
| 204 | lh.supramarginal_4 | lh.superiorparietal_11 | rh.superiorfrontal_22 | rh.postcentral_29 | rh.insula_5 | lh.lateraloccipital_18 | lh.superiorfrontal_38 |
| 205 | lh.inferiorparietal_1 | lh.inferiorparietal_6 | lh.caudalmiddlefrontal_4 | lh.precuneus_11 | rh.caudalmiddlefrontal_7 | rh.precentral_23 | lh.rostralmiddlefrontal_1 |
| 206 | lh.bankssts_2 | rh.rostralmiddlefrontal_21 | lh.precuneus_4 | lh.superiorfrontal_3 | lh.precentral_25 | rh.parstriangularis_2 | rh.supramarginal_10 |
| 207 | rh.rostralmiddlefrontal_19 | lh.superiorparietal_6 | lh.superiorfrontal_3 | lh.isthmuscingulate_5 | lh.superiorparietal_19 | rh.rostralanteriorcingulate_2 | rh.superiorfrontal_13 |
| 208 | rh.inferiorparietal_3 | rh.parsopercularis_6 | lh.precentral_21 | rh.precuneus_16 | rh.rostralmiddlefrontal_23 | rh.lateralorbitofrontal_8 | Left-Caudate |
| 209 | rh.inferiorparietal_16 | lh.paracentral_4 | rh.supramarginal_12 | lh.superiorparietal_2 | rh.postcentral_21 | rh.inferiorparietal_21 | rh.rostralanteriorcingulate_3 |
| 210 | lh.parsopercularis_7 | rh.precentral_33 | rh.fusiform_17 | lh.lingual_15 | rh.insula_14 | rh.rostralanteriorcingulate_4 | lh.parsopercularis_3 |
| 211 | lh.parsopercularis_4 | rh.superiorparietal_8 | rh.lingual_3 | rh.pericalcarine_4 | rh.paracentral_7 | rh.inferiorparietal_22 | lh.paracentral_9 |
| 212 | rh.superiorfrontal_31 | lh.lingual_15 | lh.lateraloccipital_17 | lh.superiorfrontal_41 | lh.rostralmiddlefrontal_5 | rh.insula_1 | rh.superiorparietal_16 |
| 213 | rh.rostralmiddlefrontal_22 | rh.supramarginal_10 | lh.supramarginal_2 | rh.rostralmiddlefrontal_10 | rh.rostralmiddlefrontal_14 | rh.precuneus_8 | lh.inferiorparietal_5 |
| 214 | rh.lingual_6 | rh.pericalcarine_7 | rh.superiorfrontal_42 | lh.inferiortemporal_16 | lh.precentral_33 | rh.parstriangularis_7 | rh.superiorparietal_5 |
| 215 | lh.precentral_16 | lh.supramarginal_17 | lh.precuneus_6 | lh.superiorfrontal_21 | lh.paracentral_4 | rh.isthmuscingulate_1 | lh.rostralmiddlefrontal_7 |
| 216 | lh.supramarginal_17 | rh.lingual_4 | lh.superiorfrontal_20 | lh.inferiorparietal_12 | lh.precuneus_17 | lh.supramarginal_3 | rh.parstriangularis_6 |
| 217 | rh.precentral_33 | lh.paracentral_3 | lh.paracentral_4 | rh.lingual_9 | rh.isthmuscingulate_2 | lh.rostralmiddlefrontal_13 | Right-Hippocampus |
| 218 | rh.precuneus_23 | rh.inferiorparietal_24 | lh.paracentral_6 | rh.superiorfrontal_30 | lh.paracentral_11 | rh.parsopercularis_8 | lh.rostralanteriorcingulate_5 |
| 219 | rh.middletemporal_12 | lh.paracentral_11 | lh.parsopercularis_8 | rh.lingual_5 | lh.parsopercularis_1 | rh.precuneus_14 | lh.superiorfrontal_22 |
| 220 | rh.postcentral_27 | rh.superiorparietal_18 | rh.postcentral_21 | rh.precentral_33 | lh.caudalmiddlefrontal_11 | lh.inferiorparietal_16 | rh.superiorparietal_11 |
| 221 | rh.paracentral_10 | lh.lateraloccipital_17 | rh.superiorparietal_8 | rh.parsopercularis_9 | lh.superiorfrontal_41 | lh.inferiorparietal_22 | lh.supramarginal_16 |
| 222 | lh.superiortemporal_18 | lh.superiorfrontal_42 | rh.lateraloccipital_22 | rh.precuneus_19 | lh.superiorfrontal_27 | rh.lateralorbitofrontal_9 | lh.superiorparietal_11 |
| 223 | rh.caudalanteriorcingulate_2 | rh.superiortemporal_14 | rh.inferiorparietal_14 | lh.lateraloccipital_19 | lh.rostralmiddlefrontal_23 | lh.superiorfrontal_38 | rh.superiorparietal_12 |
| 224 | rh.supramarginal_7 | rh.lateraloccipital_17 | rh.postcentral_26 | lh.cuneus_1 | rh.parsopercularis_5 | rh.precentral_13 | rh.insula_4 |
| 225 | rh.rostralmiddlefrontal_20 | rh.postcentral_21 | lh.lateraloccipital_16 | lh.lingual_11 | lh.inferiorparietal_7 | lh.paracentral_9 | lh.medialorbitofrontal_4 |
| 226 | lh.precentral_13 | lh.superiorfrontal_41 | rh.pericalcarine_4 | rh.inferiorparietal_21 | rh.precentral_23 | rh.rostralmiddlefrontal_20 | rh.superiorfrontal_24 |
| 227 | lh.precuneus_20 | lh.insula_4 | rh.lateraloccipital_19 | lh.pericalcarine_3 | rh.caudalanteriorcingulate_2 | rh.inferiorparietal_6 | rh.paracentral_11 |
| 228 | lh.rostralmiddlefrontal_22 | lh.precentral_33 | rh.insula_9 | rh.supramarginal_6 | rh.parsopercularis_7 | rh.superiorfrontal_25 | lh.posteriorcingulate_1 |

| | | | | | | | |
|---|---|---|---|---|---|---|---|
| 229 | rh.caudalmiddlefrontal_8 | lh.superiorparietal_16 | lh.precuneus_13 | lh.paracentral_5 | rh.inferiorparietal_11 | rh.inferiorparietal_3 | lh.posteriorcingulate_3 |
| 230 | lh.precentral_21 | rh.insula_1 | rh.pericalcarine_5 | lh.precentral_33 | lh.parsopercularis_9 | lh.precentral_6 | rh.rostralmiddlefrontal_16 |
| 231 | rh.superiorfrontal_42 | rh.supramarginal_13 | lh.lateraloccipital_11 | lh.lateraloccipital_16 | lh.inferiorparietal_1 | rh.lateralorbitofrontal_17 | lh.caudalmiddlefrontal_12 |
| 232 | rh.precuneus_8 | rh.superiorfrontal_2 | rh.superiorparietal_10 | rh.precentral_29 | lh.caudalmiddlefrontal_7 | rh.rostralmiddlefrontal_10 | rh.insula_15 |
| 233 | rh.bankssts_2 | rh.posteriorcingulate_8 | rh.rostralmiddlefrontal_23 | rh.inferiorparietal_15 | lh.rostralmiddlefrontal_15 | rh.rostralmiddlefrontal_18 | lh.superiorfrontal_24 |
| 234 | lh.caudalmiddlefrontal_8 | lh.paracentral_6 | lh.pericalcarine_2 | lh.supramarginal_17 | rh.insula_9 | rh.precuneus_22 | rh.parsopercularis_8 |
| 235 | rh.superiorfrontal_24 | rh.precentral_29 | rh.lingual_15 | rh.inferiortemporal_15 | rh.superiorfrontal_27 | lh.supramarginal_16 | rh.posteriorcingulate_2 |
| 236 | rh.posteriorcingulate_4 | rh.caudalmiddlefrontal_8 | lh.lingual_6 | rh.superiorfrontal_13 | rh.parstriangularis_3 | rh.precentral_12 | lh.postcentral_18 |
| 237 | rh.superiorparietal_25 | lh.isthmuscingulate_7 | lh.superiorfrontal_38 | rh.superiorparietal_14 | rh.superiorfrontal_24 | lh.precentral_12 | Left-Hippocampus |
| 238 | lh.precentral_33 | rh.lateraloccipital_6 | rh.superiorfrontal_27 | rh.postcentral_30 | rh.caudalmiddlefrontal_4 | rh.rostralmiddlefrontal_25 | lh.supramarginal_21 |
| 239 | lh.postcentral_21 | lh.superiorfrontal_45 | rh.postcentral_30 | lh.insula_15 | rh.postcentral_30 | lh.caudalanteriorcingulate_5 | lh.superiorfrontal_2 |
| 240 | rh.inferiorparietal_10 | rh.fusiform_14 | lh.precentral_16 | rh.paracentral_3 | rh.supramarginal_13 | lh.inferiorparietal_2 | lh.inferiorparietal_2 |
| 241 | lh.inferiorparietal_7 | rh.lingual_5 | rh.superiorfrontal_2 | rh.isthmuscingulate_1 | rh.superiorfrontal_14 | lh.precentral_15 | rh.caudalanteriorcingulate_2 |
| 242 | rh.inferiorparietal_11 | lh.precuneus_8 | rh.precuneus_16 | lh.parsopercularis_6 | rh.precuneus_5 | lh.superiorparietal_3 | rh.precuneus_11 |
| 243 | rh.inferiorparietal_1 | lh.caudalmiddlefrontal_1 | lh.inferiorparietal_15 | lh.superiorparietal_6 | rh.caudalmiddlefrontal_8 | rh.postcentral_27 | lh.precentral_4 |
| 244 | rh.lateraloccipital_5 | lh.precuneus_22 | rh.rostralmiddlefrontal_19 | rh.posteriorcingulate_5 | lh.supramarginal_18 | Right-Pallidum | rh.postcentral_21 |
| 245 | rh.precentral_26 | rh.paracentral_3 | lh.precentral_33 | rh.bankssts_3 | rh.paracentral_3 | lh.insula_15 | rh.precentral_9 |
| 246 | rh.precentral_16 | lh.lingual_8 | lh.paracentral_1 | rh.caudalmiddlefrontal_8 | lh.superiorparietal_2 | rh.parsopercularis_2 | rh.paracentral_12 |
| 247 | rh.postcentral_9 | rh.bankssts_3 | lh.paracentral_11 | rh.postcentral_21 | rh.superiorfrontal_17 | lh.precentral_23 | lh.precentral_26 |
| 248 | lh.precuneus_17 | rh.paracentral_10 | rh.superiortemporal_14 | lh.isthmuscingulate_6 | rh.superiorfrontal_42 | rh.caudalanteriorcingulate_4 | rh.precuneus_4 |
| 249 | lh.precuneus_18 | rh.inferiorparietal_17 | rh.postcentral_29 | lh.superiorfrontal_7 | lh.inferiorparietal_17 | rh.parahippocampal_1 | rh.superiorfrontal_37 |
| 250 | lh.lingual_15 | rh.posteriorcingulate_3 | rh.superiorfrontal_37 | rh.precentral_9 | lh.supramarginal_1 | lh.caudalmiddlefrontal_3 | lh.posteriorcingulate_9 |
| 251 | rh.parsopercularis_7 | lh.rostralmiddlefrontal_19 | lh.isthmuscingulate_7 | rh.lateraloccipital_18 | Brain-Stem | rh.isthmuscingulate_3 | rh.fusiform_16 |
| 252 | lh.superiorfrontal_29 | rh.pericalcarine_5 | rh.superiorfrontal_30 | rh.lateraloccipital_16 | rh.superiorfrontal_2 | rh.medialorbitofrontal_11 | lh.rostralmiddlefrontal_6 |
| 253 | rh.precentral_29 | lh.inferiortemporal_16 | lh.superiorfrontal_1 | lh.lateraloccipital_8 | rh.precuneus_23 | rh.medialorbitofrontal_4 | rh.caudalmiddlefrontal_3 |
| 254 | rh.caudalmiddlefrontal_3 | lh.lingual_1 | lh.precentral_4 | lh.middletemporal_6 | lh.precentral_13 | rh.inferiorparietal_16 | lh.caudalanteriorcingulate_5 |
| 255 | lh.caudalmiddlefrontal_6 | lh.superiorfrontal_3 | lh.superiorfrontal_41 | lh.supramarginal_3 | rh.precuneus_3 | rh.superiorfrontal_10 | rh.superiorfrontal_25 |
| 256 | rh.parstriangularis_3 | rh.rostralmiddlefrontal_24 | rh.inferiorparietal_17 | rh.superiortemporal_14 | lh.supramarginal_4 | rh.postcentral_29 | rh.posteriorcingulate_8 |
| 257 | rh.caudalmiddlefrontal_4 | rh.inferiortemporal_15 | lh.lingual_10 | rh.inferiorparietal_8 | lh.superiorfrontal_31 | rh.parstriangularis_3 | rh.inferiorparietal_20 |
| 258 | rh.precentral_34 | lh.superiorfrontal_28 | lh.superiorparietal_6 | lh.precentral_26 | lh.precuneus_15 | rh.inferiorparietal_26 | lh.superiorparietal_18 |
| 259 | lh.paracentral_4 | lh.precentral_26 | lh.superiortemporal_18 | lh.paracentral_11 | rh.postcentral_26 | lh.caudalmiddlefrontal_4 | rh.caudalanteriorcingulate_1 |
| 260 | lh.supramarginal_18 | rh.superiorfrontal_13 | lh.precuneus_20 | rh.paracentral_11 | lh.superiorparietal_15 | lh.paracentral_11 | rh.rostralanteriorcingulate_2 |
| 261 | lh.lateraloccipital_23 | lh.parsopercularis_8 | rh.lingual_9 | lh.fusiform_12 | rh.caudalmiddlefrontal_3 | lh.superiorfrontal_22 | lh.precuneus_5 |
| 262 | lh.inferiortemporal_7 | lh.fusiform_7 | lh.lingual_11 | rh.caudalanteriorcingulate_5 | lh.precentral_16 | lh.inferiorparietal_5 | rh.superiorparietal_26 |
| 263 | rh.inferiorparietal_23 | rh.supramarginal_8 | lh.rostralanteriorcingulate_5 | rh.superiorparietal_18 | rh.paracentral_10 | rh.superiorfrontal_27 | lh.superiorfrontal_17 |
| 264 | lh.superiorparietal_10 | lh.middletemporal_6 | rh.caudalmiddlefrontal_5 | rh.posteriorcingulate_2 | rh.supramarginal_7 | rh.postcentral_21 | rh.superiorparietal_17 |
| 265 | lh.lingual_2 | rh.paracentral_11 | rh.superiorparietal_22 | rh.supramarginal_13 | rh.middletemporal_12 | rh.insula_11 | lh.rostralanteriorcingulate_4 |
| 266 | lh.bankssts_3 | rh.rostralmiddlefrontal_18 | rh.parsopercularis_9 | rh.isthmuscingulate_4 | lh.isthmuscingulate_1 | rh.inferiorparietal_11 | rh.precentral_3 |
| 267 | rh.superiorparietal_15 | rh.supramarginal_7 | lh.supramarginal_4 | lh.superiorparietal_11 | lh.supramarginal_17 | rh.superiorfrontal_15 | lh.isthmuscingulate_2 |
| 268 | rh.bankssts_5 | rh.postcentral_27 | rh.supramarginal_13 | lh.posteriorcingulate_9 | lh.superiorfrontal_22 | lh.caudalanteriorcingulate_4 | lh.precuneus_22 |
| 269 | lh.caudalmiddlefrontal_11 | lh.pericalcarine_2 | rh.superiorfrontal_19 | rh.rostralmiddlefrontal_21 | rh.superiorfrontal_22 | lh.precuneus_1 | rh.inferiorparietal_25 |
| 270 | rh.caudalmiddlefrontal_9 | lh.cuneus_1 | rh.insula_4 | lh.cuneus_2 | lh.caudalmiddlefrontal_2 | lh.isthmuscingulate_7 | lh.paracentral_6 |
| 271 | rh.postcentral_13 | lh.supramarginal_21 | rh.insula_8 | lh.caudalanteriorcingulate_2 | lh.paracentral_3 | rh.parsopercularis_9 | lh.isthmuscingulate_4 |
| 272 | rh.lingual_15 | lh.superiorfrontal_37 | rh.insula_14 | lh.insula_16 | rh.precuneus_8 | rh.precentral_20 | rh.isthmuscingulate_4 |
| 273 | lh.lingual_7 | lh.supramarginal_4 | lh.superiorparietal_11 | rh.paracentral_10 | lh.bankssts_2 | rh.inferiorparietal_23 | lh.precentral_8 |
| 274 | lh.bankssts_5 | lh.rostralmiddlefrontal_9 | lh.parsopercularis_6 | rh.superiorparietal_9 | rh.postcentral_27 | lh.superiorparietal_18 | rh.precuneus_7 |

| | | | | | | | |
|---|---|---|---|---|---|---|---|
| 275 | lh.rostralmiddlefrontal_15 | rh.lingual_10 | lh.precentral_23 | rh.parsopercularis_6 | rh.precentral_34 | Left-Accumbens-area | rh.parsopercularis_3 |
| 276 | lh.postcentral_8 | lh.superiorfrontal_21 | lh.rostralmiddlefrontal_22 | lh.paracentral_6 | rh.rostralmiddlefrontal_2 | lh.inferiorparietal_3 | lh.paracentral_5 |
| 277 | lh.transversetemporal_2 | rh.lingual_12 | rh.superiorparietal_9 | lh.caudalmiddlefrontal_1 | rh.precuneus_7 | rh.precentral_14 | lh.rostralmiddlefrontal_17 |
| 278 | lh.insula_12 | rh.superiorfrontal_18 | lh.isthmuscingulate_1 | lh.posteriorcingulate_2 | lh.caudalanteriorcingulate_5 | rh.inferiorparietal_20 | rh.isthmuscingulate_2 |
| 279 | rh.parsopercularis_5 | lh.lateraloccipital_11 | lh.inferiorparietal_3 | lh.superiorfrontal_23 | lh.precentral_21 | rh.supramarginal_6 | rh.precentral_7 |
| 280 | rh.postcentral_14 | rh.rostralmiddlefrontal_23 | lh.precentral_26 | lh.precuneus_22 | rh.paracentral_2 | rh.precentral_33 | lh.superiorparietal_20 |
| 281 | lh.parsopercularis_1 | rh.precentral_26 | lh.precuneus_22 | lh.postcentral_19 | lh.superiorfrontal_38 | lh.rostralmiddlefrontal_9 | rh.insula_16 |
| 282 | lh.superiorfrontal_22 | lh.superiortemporal_18 | rh.insula_7 | lh.paracentral_7 | lh.superiorfrontal_2 | lh.supramarginal_18 | rh.insula_6 |
| 283 | lh.supramarginal_7 | lh.supramarginal_7 | lh.superiorparietal_24 | lh.superiorparietal_23 | rh.inferiorparietal_23 | rh.caudalanteriorcingulate_6 | lh.superiorfrontal_13 |
| 284 | lh.inferiorparietal_16 | rh.fusiform_9 | lh.inferiortemporal_16 | rh.posteriorcingulate_8 | rh.superiortemporal_14 | lh.superiorfrontal_19 | rh.insula_12 |
| 285 | rh.precuneus_7 | lh.superiorfrontal_1 | lh.superiorparietal_2 | lh.fusiform_6 | lh.parsopercularis_3 | rh.inferiorparietal_2 | rh.posteriorcingulate_3 |
| 286 | lh.superiorfrontal_41 | rh.lingual_15 | lh.postcentral_19 | lh.superiorfrontal_24 | rh.precentral_26 | rh.superiortemporal_8 | rh.caudalmiddlefrontal_8 |
| 287 | lh.caudalmiddlefrontal_7 | lh.precuneus_20 | lh.precuneus_18 | rh.supramarginal_10 | rh.posteriorcingulate_4 | rh.lateraloccipital_6 | rh.inferiorparietal_6 |
| 288 | lh.postcentral_30 | rh.superiorfrontal_17 | lh.cuneus_1 | rh.rostralmiddlefrontal_23 | lh.superiorfrontal_42 | rh.insula_16 | lh.parsopercularis_5 |
| 289 | rh.superiorfrontal_27 | lh.inferiorparietal_5 | lh.lateraloccipital_4 | lh.paracentral_9 | rh.lateraloccipital_5 | rh.lateralorbitofrontal_4 | rh.inferiorparietal_18 |
| 290 | rh.caudalmiddlefrontal_7 | rh.precuneus_8 | lh.caudalmiddlefrontal_8 | rh.superiorfrontal_17 | lh.rostralanteriorcingulate_2 | rh.caudalanteriorcingulate_1 | lh.isthmuscingulate_3 |
| 291 | rh.bankssts_3 | rh.precentral_16 | Right-Amygdala | lh.rostralmiddlefrontal_19 | lh.paracentral_7 | rh.parahippocampal_3 | rh.precuneus_9 |
| 292 | lh.postcentral_13 | rh.precuneus_9 | rh.paracentral_3 | lh.insula_4 | lh.precentral_26 | rh.supramarginal_2 | lh.parstriangularis_6 |
| 293 | lh.postcentral_10 | rh.caudalanteriorcingulate_2 | rh.paracentral_11 | rh.superiorfrontal_37 | rh.inferiorparietal_10 | rh.rostralmiddlefrontal_11 | rh.precuneus_19 |
| 294 | lh.parsopercularis_9 | rh.lateraloccipital_8 | lh.superiorparietal_20 | rh.postcentral_6 | lh.postcentral_21 | lh.fusiform_7 | lh.supramarginal_15 |
| 295 | lh.caudalanteriorcingulate_5 | lh.postcentral_21 | rh.supramarginal_8 | rh.superiorfrontal_19 | rh.inferiorparietal_24 | rh.caudalmiddlefrontal_1 | rh.superiorfrontal_42 |
| 296 | lh.superiorparietal_19 | lh.precentral_8 | lh.middletemporal_6 | lh.precuneus_8 | rh.precentral_36 | lh.precuneus_2 | rh.superiorfrontal_28 |
| 297 | rh.rostralmiddlefrontal_23 | lh.rostralmiddlefrontal_13 | lh.superiorfrontal_21 | lh.precentral_28 | rh.paracentral_11 | rh.middletemporal_2 | rh.inferiorparietal_4 |
| 298 | rh.paracentral_3 | rh.caudalmiddlefrontal_3 | rh.rostralmiddlefrontal_20 | rh.superiorfrontal_1 | lh.precuneus_10 | lh.caudalmiddlefrontal_8 | lh.superiorparietal_22 |
| 299 | lh.superiorfrontal_31 | lh.paracentral_7 | rh.lateraloccipital_2 | lh.supramarginal_7 | rh.inferiorparietal_6 | rh.parstriangularis_8 | lh.superiorparietal_6 |
| 300 | lh.precentral_11 | rh.insula_5 | lh.superiorfrontal_42 | rh.supramarginal_7 | rh.superiorfrontal_28 | rh.inferiorparietal_19 | rh.caudalmiddlefrontal_2 |
| 301 | rh.precentral_36 | rh.superiorfrontal_38 | lh.superiorfrontal_23 | lh.superiorfrontal_34 | rh.postcentral_8 | rh.precentral_27 | rh.rostralmiddlefrontal_7 |
| 302 | rh.superiorfrontal_22 | lh.inferiorparietal_1 | lh.superiorfrontal_26 | rh.lingual_15 | lh.precuneus_3 | lh.parsopercularis_6 | rh.postcentral_8 |
| 303 | rh.supramarginal_9 | lh.precentral_28 | rh.superiorfrontal_6 | rh.postcentral_27 | Right-Amygdala | lh.lateraloccipital_21 | lh.precuneus_8 |
| 304 | rh.inferiortemporal_13 | rh.rostralmiddlefrontal_19 | lh.superiorfrontal_7 | rh.precentral_26 | rh.transversetemporal_3 | lh.precentral_24 | lh.inferiorparietal_10 |
| 305 | lh.precentral_20 | lh.lingual_10 | rh.superiorparietal_11 | lh.isthmuscingulate_7 | lh.precuneus_21 | lh.lateraloccipital_5 | rh.isthmuscingulate_1 |
| 306 | lh.precuneus_3 | lh.lateraloccipital_4 | lh.precuneus_3 | rh.caudalanteriorcingulate_3 | rh.inferiorparietal_4 | rh.supramarginal_19 | rh.precentral_34 |
| 307 | rh.inferiorparietal_24 | rh.superiorfrontal_30 | rh.paracentral_10 | rh.lateraloccipital_19 | lh.postcentral_30 | lh.superiorfrontal_1 | rh.insula_13 |
| 308 | lh.superiorfrontal_2 | lh.fusiform_12 | rh.lateraloccipital_11 | rh.insula_1 | lh.superiorfrontal_20 | rh.inferiorparietal_25 | rh.precentral_25 |
| 309 | lh.supramarginal_12 | rh.precuneus_23 | rh.bankssts_3 | lh.superiorfrontal_26 | rh.postcentral_9 | lh.postcentral_30 | rh.supramarginal_17 |
| 310 | lh.superiorparietal_15 | rh.fusiform_15 | rh.caudalanteriorcingulate_6 | rh.fusiform_12 | rh.lingual_6 | rh.superiorfrontal_38 | rh.isthmuscingulate_3 |
| 311 | lh.precuneus_10 | lh.precuneus_3 | rh.precentral_26 | rh.fusiform_15 | lh.postcentral_10 | lh.precentral_35 | lh.inferiorparietal_13 |
| 312 | rh.superiorparietal_8 | lh.superiorfrontal_7 | lh.paracentral_3 | lh.fusiform_15 | lh.precentral_20 | lh.isthmuscingulate_2 | lh.precuneus_9 |
| 313 | lh.caudalmiddlefrontal_2 | lh.pericalcarine_3 | rh.superiorfrontal_34 | lh.supramarginal_4 | lh.inferiorparietal_16 | lh.caudalmiddlefrontal_1 | lh.superiorparietal_28 |
| 314 | lh.fusiform_11 | rh.inferiorparietal_9 | rh.paracentral_7 | lh.rostralmiddlefrontal_9 | lh.superiortemporal_18 | rh.postcentral_2 | lh.precentral_3 |
| 315 | lh.paracentral_3 | rh.inferiorparietal_25 | rh.fusiform_14 | rh.rostralmiddlefrontal_24 | lh.bankssts_5 | lh.superiorfrontal_41 | rh.precuneus_6 |
| 316 | lh.precuneus_21 | lh.caudalanteriorcingulate_4 | rh.superiorfrontal_36 | lh.superiortemporal_18 | rh.bankssts_2 | rh.precentral_8 | rh.supramarginal_3 |
| 317 | lh.superiorfrontal_38 | rh.precuneus_7 | lh.paracentral_7 | lh.superiorparietal_16 | rh.superiorfrontal_13 | lh.superiorparietal_7 | lh.inferiorparietal_18 |
| 318 | rh.inferiorparietal_6 | rh.postcentral_6 | rh.supramarginal_7 | lh.precuneus_20 | rh.rostralmiddlefrontal_16 | lh.caudalmiddlefrontal_9 | lh.superiorfrontal_27 |
| 319 | lh.parsopercularis_3 | lh.lateraloccipital_16 | rh.superiorparietal_18 | rh.precentral_16 | lh.precentral_12 | Right-Thalamus-Proper | rh.postcentral_29 |
| 320 | lh.precentral_26 | rh.superiorparietal_25 | rh.precuneus_11 | rh.lateraloccipital_11 | lh.superiorfrontal_32 | rh.superiorfrontal_1 | lh.insula_8 |

| | | | | | | | |
|---|---|---|---|---|---|---|---|
| 321 | lh.lateraloccipital_18 | lh.superiorfrontal_26 | lh.superiorparietal_16 | lh.supramarginal_21 | lh.precentral_11 | lh.fusiform_17 | lh.isthmuscingulate_7 |
| 322 | rh.superiorfrontal_28 | rh.lateraloccipital_9 | lh.lateraloccipital_19 | rh.precuneus_8 | rh.bankssts_5 | rh.precentral_29 | rh.inferiorparietal_24 |
| 323 | rh.superiorfrontal_17 | rh.inferiorparietal_19 | lh.postcentral_10 | rh.posteriorcingulate_3 | lh.supramarginal_7 | lh.rostralmiddlefrontal_4 | rh.superiorfrontal_3 |
| 324 | rh.postcentral_8 | lh.paracentral_9 | rh.lateraloccipital_9 | rh.caudalmiddlefrontal_3 | rh.rostralanteriorcingulate_3 | rh.postcentral_14 | lh.inferiorparietal_11 |
| 325 | rh.lingual_5 | rh.rostralmiddlefrontal_20 | rh.precuneus_23 | lh.superiorfrontal_18 | rh.superiorfrontal_26 | lh.precentral_26 | lh.precentral_6 |
| 326 | rh.insula_14 | lh.superiorfrontal_23 | lh.supramarginal_3 | rh.paracentral_5 | lh.postcentral_18 | rh.superiorparietal_26 | lh.inferiorparietal_21 |
| 327 | rh.insula_9 | rh.inferiorparietal_10 | rh.precuneus_5 | lh.postcentral_21 | rh.superiorfrontal_25 | Left-Thalamus-Proper | lh.precuneus_16 |
| 328 | lh.superiorfrontal_20 | lh.postcentral_19 | rh.rostralmiddlefrontal_18 | lh.parsopercularis_8 | rh.inferiorparietal_26 | lh.fusiform_3 | lh.isthmuscingulate_5 |
| 329 | rh.precentral_10 | lh.fusiform_6 | lh.inferiorparietal_5 | rh.supramarginal_1 | rh.precentral_16 | lh.isthmuscingulate_6 | lh.precuneus_10 |
| 330 | rh.superiortemporal_21 | lh.postcentral_10 | rh.precuneus_18 | rh.caudalanteriorcingulate_2 | rh.supramarginal_9 | lh.insula_12 | rh.supramarginal_19 |
| 331 | rh.inferiorparietal_4 | rh.supramarginal_1 | rh.precuneus_7 | rh.inferiorparietal_17 | lh.inferiortemporal_7 | rh.superiorparietal_8 | rh.precentral_35 |
| 332 | lh.postcentral_9 | lh.caudalmiddlefrontal_11 | lh.precentral_19 | rh.supramarginal_8 | rh.lateralorbitofrontal_6 | lh.lateraloccipital_23 | lh.precuneus_11 |
| 333 | lh.inferiorparietal_6 | lh.parstriangularis_2 | Left-Amygdala | rh.lateraloccipital_2 | lh.superiorfrontal_21 | rh.inferiorparietal_5 | lh.inferiorparietal_1 |
| 334 | rh.superiorfrontal_14 | lh.precuneus_17 | rh.lateraloccipital_8 | lh.superiorfrontal_35 | lh.lateraloccipital_23 | lh.isthmuscingulate_1 | lh.superiorfrontal_20 |
| 335 | rh.supramarginal_14 | lh.supramarginal_18 | rh.precentral_29 | lh.precuneus_3 | rh.postcentral_14 | lh.parsopercularis_4 | rh.superiorfrontal_6 |
| 336 | rh.lateraloccipital_3 | rh.paracentral_5 | rh.precuneus_8 | rh.rostralmiddlefrontal_18 | lh.lateraloccipital_18 | lh.inferiorparietal_9 | lh.parsopercularis_10 |
| 337 | lh.rostralmiddlefrontal_5 | rh.precentral_27 | lh.paracentral_9 | rh.cuneus_6 | rh.precentral_10 | lh.superiortemporal_24 | rh.precuneus_14 |
| 338 | rh.superiorfrontal_41 | rh.precentral_3 | rh.superiorfrontal_26 | lh.inferiorparietal_5 | lh.superiorparietal_22 | Right-Putamen | rh.supramarginal_11 |
| 339 | rh.paracentral_11 | rh.paracentral_2 | rh.inferiortemporal_15 | rh.precentral_14 | rh.superiorfrontal_41 | rh.insula_9 | rh.precentral_15 |
| 340 | lh.rostralmiddlefrontal_23 | rh.inferiorparietal_6 | rh.fusiform_9 | rh.superiorfrontal_16 | rh.lateraloccipital_3 | lh.caudalmiddlefrontal_10 | lh.insula_3 |
| 341 | rh.lateralorbitofrontal_6 | rh.pericalcarine_4 | lh.superiorparietal_23 | lh.precentral_3 | lh.inferiorparietal_9 | rh.superiorfrontal_20 | lh.paracentral_10 |
| 342 | rh.rostralmiddlefrontal_14 | lh.lateraloccipital_19 | lh.rostralmiddlefrontal_15 | rh.precentral_27 | lh.insula_12 | lh.caudalmiddlefrontal_5 | lh.inferiorparietal_15 |
| 343 | lh.superiorfrontal_43 | lh.posteriorcingulate_7 | rh.superiorfrontal_1 | lh.fusiform_10 | rh.precuneus_11 | rh.posteriorcingulate_4 | rh.postcentral_24 |
| 344 | lh.rostralanteriorcingulate_2 | lh.superiorfrontal_24 | lh.postcentral_13 | lh.paracentral_1 | rh.paracentral_5 | lh.rostralmiddlefrontal_15 | lh.inferiorparietal_8 |
| 345 | lh.inferiorparietal_9 | rh.lateraloccipital_18 | rh.cuneus_6 | rh.lateraloccipital_4 | lh.postcentral_13 | lh.postcentral_21 | lh.insula_11 |
| 346 | lh.middletemporal_6 | rh.superiorfrontal_37 | rh.precentral_9 | rh.paracentral_2 | rh.superiorparietal_5 | lh.medialorbitofrontal_6 | lh.precuneus_21 |
| 347 | lh.precentral_5 | lh.superiorfrontal_35 | lh.lateraloccipital_9 | rh.superiorfrontal_36 | lh.bankssts_3 | lh.posteriorcingulate_6 | lh.isthmuscingulate_6 |
| 348 | lh.superiorparietal_1 | lh.lateraloccipital_8 | rh.inferiorparietal_25 | lh.inferiorparietal_1 | rh.supramarginal_3 | rh.parsopercularis_4 | lh.postcentral_7 |
| 349 | lh.superiorfrontal_42 | rh.lingual_9 | lh.parsopercularis_4 | rh.precuneus_7 | rh.supramarginal_14 | lh.parsopercularis_9 | lh.superiorfrontal_19 |
| 350 | rh.paracentral_2 | lh.caudalmiddlefrontal_2 | rh.lateraloccipital_16 | lh.paracentral_10 | lh.superiorfrontal_3 | lh.superiorfrontal_16 | rh.inferiorparietal_22 |
| 351 | rh.inferiortemporal_15 | lh.caudalmiddlefrontal_6 | lh.fusiform_7 | lh.rostralmiddlefrontal_13 | lh.superiorfrontal_23 | lh.lingual_7 | rh.precentral_36 |
| 352 | lh.supramarginal_19 | rh.middletemporal_16 | rh.supramarginal_1 | lh.fusiform_16 | lh.inferiorparietal_4 | rh.supramarginal_8 | lh.superiorfrontal_9 |
| 353 | lh.inferiorparietal_4 | rh.lateraloccipital_2 | lh.lingual_17 | rh.paracentral_12 | rh.parsopercularis_1 | lh.posteriorcingulate_1 | lh.superiorfrontal_1 |
| 354 | rh.rostralmiddlefrontal_16 | lh.parsopercularis_7 | rh.precentral_14 | rh.rostralmiddlefrontal_19 | lh.precentral_32 | lh.precuneus_16 | lh.precentral_13 |
| 355 | rh.superiorfrontal_25 | rh.precentral_36 | lh.precentral_8 | rh.superiorfrontal_21 | lh.superiorfrontal_35 | rh.lateraloccipital_22 | rh.inferiorparietal_23 |
| 356 | rh.precentral_27 | rh.inferiorparietal_16 | lh.transversetemporal_2 | rh.middletemporal_16 | lh.precentral_28 | lh.caudalanteriorcingulate_1 | rh.lateralorbitofrontal_6 |
| 357 | rh.fusiform_15 | lh.paracentral_10 | rh.caudalmiddlefrontal_8 | lh.precentral_8 | lh.parstriangularis_6 | rh.insula_12 | lh.superiorfrontal_4 |
| 358 | lh.precentral_32 | rh.inferiorparietal_1 | rh.lateraloccipital_18 | rh.lateraloccipital_10 | rh.postcentral_13 | lh.inferiorparietal_7 | rh.bankssts_1 |
| 359 | lh.precentral_28 | lh.caudalmiddlefrontal_8 | rh.postcentral_27 | lh.postcentral_10 | lh.postcentral_8 | lh.precentral_3 | rh.precuneus_18 |
| 360 | lh.superiorparietal_22 | rh.parsopercularis_7 | rh.rostralmiddlefrontal_22 | rh.paracentral_4 | lh.rostralmiddlefrontal_17 | rh.superiorparietal_16 | lh.precentral_32 |
| 361 | rh.pericalcarine_6 | lh.precentral_3 | rh.rostralmiddlefrontal_27 | lh.cuneus_6 | rh.bankssts_3 | lh.inferiorparietal_13 | rh.precentral_23 |
| 362 | rh.superiorfrontal_13 | lh.rostralmiddlefrontal_26 | lh.precentral_3 | rh.precuneus_23 | rh.precuneus_18 | rh.lateralorbitofrontal_16 | lh.paracentral_11 |
| 363 | rh.supramarginal_3 | rh.caudalmiddlefrontal_9 | rh.inferiorparietal_6 | rh.inferiorparietal_6 | lh.superiorfrontal_43 | rh.superiorfrontal_23 | rh.paracentral_7 |
| 364 | rh.middletemporal_16 | rh.superiorfrontal_19 | lh.fusiform_15 | lh.inferiorparietal_3 | lh.superiorparietal_18 | rh.precentral_22 | rh.superiorparietal_2 |
| 365 | lh.postcentral_18 | lh.inferiorparietal_3 | lh.insula_7 | lh.pericalcarine_1 | rh.superiorparietal_8 | lh.paracentral_6 | rh.inferiorparietal_9 |
| 366 | rh.precuneus_18 | rh.rostralmiddlefrontal_14 | rh.rostralmiddlefrontal_14 | rh.superiorfrontal_5 | lh.rostralanteriorcingulate_5 | lh.lingual_17 | lh.supramarginal_3 |

| | | | | | | | |
|---|---|---|---|---|---|---|---|
| 367 | Right-Amygdala | rh.superiorfrontal_14 | rh.precentral_33 | rh.superiorparietal_25 | lh.inferiorparietal_13 | rh.posteriorcingulate_2 | rh.precuneus_8 |
| 368 | lh.insula_7 | rh.supramarginal_9 | rh.paracentral_4 | rh.superiorfrontal_26 | lh.lingual_15 | lh.caudalmiddlefrontal_13 | lh.precuneus_3 |
| 369 | lh.precentral_12 | rh.transversetemporal_3 | lh.precuneus_8 | rh.insula_5 | rh.superiorparietal_12 | rh.isthmuscingulate_6 | lh.precentral_28 |
| 370 | lh.lateraloccipital_21 | rh.superiorfrontal_26 | lh.inferiorparietal_1 | lh.postcentral_5 | rh.inferiortemporal_13 | lh.precentral_8 | lh.insula_6 |
| 371 | lh.lateraloccipital_5 | lh.fusiform_10 | rh.inferiorparietal_15 | rh.precuneus_9 | rh.superiorfrontal_34 | lh.paracentral_7 | lh.insula_1 |
| 372 | rh.rostralanteriorcingulate_3 | lh.fusiform_16 | lh.supramarginal_21 | rh.superiorfrontal_6 | rh.parsopercularis_8 | rh.precuneus_7 | lh.rostralmiddlefrontal_8 |
| 373 | lh.precentral_35 | lh.superiorparietal_23 | lh.supramarginal_7 | rh.inferiorparietal_10 | lh.insula_10 | rh.inferiorparietal_17 | rh.precentral_24 |
| 374 | lh.parahippocampal_5 | rh.fusiform_12 | lh.parsopercularis_7 | lh.caudalmiddlefrontal_11 | lh.insula_7 | lh.lingual_15 | lh.inferiorparietal_4 |
| 375 | rh.superiorfrontal_2 | lh.rostralmiddlefrontal_5 | rh.pericalcarine_3 | rh.superiorfrontal_33 | lh.lingual_7 | rh.precentral_15 | rh.paracentral_8 |
| 376 | rh.precentral_8 | rh.precuneus_11 | rh.superiorparietal_25 | rh.precuneus_11 | rh.precentral_13 | rh.superiorparietal_28 | Left-Amygdala |
| 377 | rh.precuneus_2 | lh.parsopercularis_4 | rh.parsopercularis_7 | rh.rostralmiddlefrontal_20 | lh.precuneus_18 | lh.rostralmiddlefrontal_6 | rh.precentral_11 |
| 378 | lh.paracentral_7 | rh.insula_8 | rh.rostralanteriorcingulate_3 | lh.supramarginal_18 | lh.rostralanteriorcingulate_3 | rh.supramarginal_12 | lh.rostralanteriorcingulate_1 |
| 379 | rh.superiorparietal_2 | rh.inferiorparietal_3 | lh.rostralmiddlefrontal_26 | lh.caudalmiddlefrontal_2 | rh.middletemporal_16 | rh.temporalpole_2 | lh.supramarginal_10 |
| 380 | rh.precuneus_21 | lh.isthmuscingulate_1 | rh.superiorparietal_2 | lh.superiorfrontal_25 | lh.inferiorparietal_6 | lh.posteriorcingulate_9 | lh.precentral_25 |
| 381 | rh.precuneus_11 | lh.postcentral_5 | lh.cuneus_2 | lh.lateraloccipital_20 | rh.superiorparietal_16 | lh.paracentral_5 | rh.supramarginal_6 |
| 382 | lh.postcentral_12 | rh.insula_4 | rh.inferiorparietal_19 | lh.bankssts_6 | rh.superiorparietal_2 | lh.superiorfrontal_4 | lh.insula_2 |
| 383 | lh.inferiorparietal_3 | rh.precuneus_2 | lh.fusiform_10 | lh.precuneus_17 | rh.precentral_35 | rh.medialorbitofrontal_8 | rh.superiorfrontal_18 |
| 384 | lh.inferiorparietal_13 | lh.bankssts_6 | rh.superiorfrontal_33 | rh.rostralmiddlefrontal_14 | lh.supramarginal_12 | rh.lateralorbitofrontal_13 | lh.insula_7 |
| 385 | rh.supramarginal_1 | lh.paracentral_1 | rh.precuneus_13 | lh.lateraloccipital_9 | lh.superiorparietal_21 | lh.precentral_4 | rh.supramarginal_4 |
| 386 | lh.parstriangularis_6 | rh.paracentral_4 | rh.supramarginal_9 | rh.precuneus_2 | lh.supramarginal_19 | lh.inferiorparietal_14 | rh.precuneus_23 |
| 387 | lh.inferiortemporal_16 | rh.precentral_14 | lh.precentral_11 | lh.superiorfrontal_19 | lh.postcentral_9 | rh.superiorfrontal_5 | rh.supramarginal_12 |
| 388 | lh.bankssts_6 | rh.lateraloccipital_16 | lh.postcentral_21 | rh.lingual_16 | lh.lingual_2 | lh.supramarginal_10 | lh.inferiorparietal_9 |
| 389 | rh.superiorfrontal_26 | lh.rostralmiddlefrontal_22 | lh.rostralanteriorcingulate_2 | rh.postcentral_11 | lh.rostralmiddlefrontal_6 | lh.inferiorparietal_18 | rh.supramarginal_7 |
| 390 | rh.precentral_32 | rh.postcentral_14 | rh.paracentral_12 | rh.cuneus_5 | rh.precentral_27 | lh.precentral_21 | rh.superiorfrontal_23 |
| 391 | rh.inferiorparietal_7 | lh.fusiform_15 | lh.fusiform_12 | rh.inferiorparietal_25 | lh.precentral_5 | lh.precentral_7 | rh.superiorfrontal_41 |
| 392 | rh.middletemporal_4 | rh.lateraloccipital_11 | rh.transversetemporal_3 | lh.superiorparietal_17 | lh.paracentral_10 | lh.postcentral_8 | rh.isthmuscingulate_6 |
| 393 | lh.lingual_17 | rh.rostralmiddlefrontal_22 | rh.superiorfrontal_5 | rh.precentral_36 | rh.paracentral_4 | rh.caudalanteriorcingulate_2 | lh.superiorfrontal_37 |
| 394 | rh.supramarginal_20 | rh.superiorfrontal_16 | lh.cuneus_4 | rh.rostralmiddlefrontal_27 | rh.parsopercularis_4 | lh.insula_14 | lh.insula_5 |
| 395 | rh.inferiorparietal_26 | lh.cuneus_2 | rh.superiorparietal_5 | rh.rostralmiddlefrontal_4 | rh.superiorparietal_26 | rh.lingual_17 | rh.supramarginal_5 |
| 396 | lh.rostralmiddlefrontal_17 | rh.lateraloccipital_19 | rh.superiorfrontal_12 | rh.superiorfrontal_14 | lh.inferiorparietal_10 | lh.insula_11 | rh.medialorbitofrontal_4 |
| 397 | rh.postcentral_7 | lh.inferiorparietal_22 | rh.supramarginal_3 | rh.middletemporal_15 | rh.precentral_8 | lh.inferiortemporal_15 | rh.superiorparietal_29 |
| 398 | rh.superiorparietal_5 | lh.lingual_11 | lh.fusiform_16 | lh.superiorfrontal_6 | lh.transversetemporal_2 | lh.superiortemporal_11 | Left-Accumbens-area |
| 399 | lh.superiortemporal_9 | lh.inferiorparietal_7 | rh.paracentral_5 | rh.precentral_30 | rh.inferiorparietal_7 | rh.paracentral_12 | rh.paracentral_10 |
| 400 | rh.precentral_35 | rh.postcentral_11 | lh.precuneus_17 | lh.rostralmiddlefrontal_5 | rh.lingual_15 | Left-Putamen | lh.precuneus_2 |
| 401 | rh.fusiform_14 | lh.postcentral_30 | rh.precentral_27 | rh.pericalcarine_3 | rh.supramarginal_20 | lh.superiorfrontal_2 | rh.precentral_26 |
| 402 | lh.supramarginal_6 | rh.parstriangularis_8 | lh.precuneus_16 | lh.caudalmiddlefrontal_6 | lh.rostralmiddlefrontal_21 | lh.inferiorparietal_8 | rh.lateralorbitofrontal_15 |
| 403 | lh.superiorparietal_18 | rh.paracentral_12 | rh.fusiform_12 | rh.cuneus_8 | lh.middletemporal_6 | rh.lateralorbitofrontal_5 | rh.middletemporal_16 |
| 404 | rh.rostralmiddlefrontal_2 | rh.supramarginal_3 | lh.parsopercularis_1 | rh.superiorparietal_5 | lh.rostralmiddlefrontal_10 | rh.precentral_24 | rh.middletemporal_2 |
| 405 | rh.postcentral_2 | rh.postcentral_9 | lh.parstriangularis_2 | rh.supramarginal_9 | lh.supramarginal_6 | rh.superiorparietal_17 | rh.rostralmiddlefrontal_11 |
| 406 | lh.paracentral_10 | rh.superiorparietal_5 | rh.inferiorparietal_3 | lh.superiorfrontal_4 | lh.fusiform_11 | rh.precentral_9 | rh.insula_5 |
| 407 | lh.superiortemporal_20 | rh.isthmuscingulate_2 | lh.precentral_28 | lh.superiorfrontal_40 | rh.superiortemporal_21 | rh.inferiortemporal_5 | lh.precentral_2 |
| 408 | rh.paracentral_5 | lh.superiorfrontal_34 | lh.posteriorcingulate_7 | rh.caudalmiddlefrontal_9 | rh.rostralmiddlefrontal_7 | rh.inferiorparietal_14 | rh.lateraloccipital_3 |
| 409 | lh.superiorfrontal_3 | lh.precentral_12 | lh.superiorfrontal_25 | lh.parstriangularis_2 | lh.paracentral_9 | rh.caudalmiddlefrontal_5 | lh.precuneus_1 |
| 410 | lh.supramarginal_13 | lh.caudalanteriorcingulate_5 | lh.rostralmiddlefrontal_23 | lh.precentral_19 | lh.precentral_3 | rh.paracentral_2 | rh.supramarginal_20 |
| 411 | rh.parsopercularis_8 | rh.postcentral_8 | lh.caudalmiddlefrontal_11 | lh.lingual_5 | lh.paracentral_1 | lh.supramarginal_15 | lh.precentral_20 |
| 412 | rh.postcentral_11 | lh.precentral_13 | rh.precentral_30 | lh.lateraloccipital_2 | rh.lingual_5 | lh.precentral_13 | lh.supramarginal_6 |

| | | | | | | | |
|---|---|---|---|---|---|---|---|
| 413 | rh.superiorparietal_12 | lh.precentral_35 | lh.postcentral_5 | lh.rostralmiddlefrontal_10 | rh.postcentral_2 | rh.posteriorcingulate_6 | rh.precentral_19 |
| 414 | lh.superiorfrontal_26 | lh.inferiorparietal_9 | lh.inferiorparietal_4 | rh.inferiorparietal_9 | lh.superiorparietal_12 | lh.superiorparietal_28 | rh.superiorfrontal_16 |
| 415 | lh.rostralanteriorcingulate_5 | rh.precuneus_5 | lh.rostralmiddlefrontal_5 | rh.parsopercularis_7 | rh.precentral_14 | rh.supramarginal_17 | rh.precuneus_1 |
| 416 | rh.rostralanteriorcingulate_4 | rh.caudalanteriorcingulate_4 | rh.inferiorparietal_16 | lh.precentral_12 | rh.rostralmiddlefrontal_3 | rh.precuneus_18 | rh.rostralmiddlefrontal_15 |
| 417 | rh.lateraloccipital_7 | lh.posteriorcingulate_3 | rh.parstriangularis_8 | rh.supramarginal_3 | lh.caudalmiddlefrontal_12 | lh.caudalmiddlefrontal_12 | rh.rostralmiddlefrontal_6 |
| 418 | rh.lateraloccipital_22 | lh.precentral_11 | rh.precentral_16 | rh.inferiorparietal_19 | lh.precentral_19 | lh.paracentral_1 | rh.inferiorparietal_7 |
| 419 | lh.supramarginal_20 | lh.caudalmiddlefrontal_7 | lh.caudalmiddlefrontal_6 | rh.inferiorparietal_1 | lh.superiorparietal_4 | rh.parstriangularis_4 | lh.precentral_16 |
| 420 | lh.cuneus_1 | rh.parsopercularis_5 | lh.insula_12 | rh.precentral_3 | rh.superiorfrontal_35 | lh.precuneus_18 | lh.supramarginal_2 |
| 421 | rh.postcentral_5 | lh.postcentral_13 | lh.superiorfrontal_34 | lh.precentral_35 | rh.supramarginal_1 | lh.rostralmiddlefrontal_12 | rh.middletemporal_12 |
| 422 | rh.fusiform_17 | rh.superiorfrontal_1 | rh.superiortemporal_21 | rh.precentral_24 | rh.rostralmiddlefrontal_4 | rh.superiorparietal_20 | rh.inferiorparietal_5 |
| 423 | lh.postcentral_5 | rh.precentral_32 | rh.caudalmiddlefrontal_9 | lh.superiorfrontal_30 | lh.superiorfrontal_26 | rh.superiorfrontal_30 | rh.postcentral_2 |
| 424 | rh.parsopercularis_1 | rh.middletemporal_15 | lh.precentral_5 | lh.cuneus_4 | lh.superiorparietal_1 | lh.supramarginal_2 | rh.precentral_30 |
| 425 | rh.caudalanteriorcingulate_6 | lh.cuneus_6 | rh.superiorfrontal_21 | rh.precentral_35 | rh.postcentral_11 | lh.precentral_19 | lh.superiorfrontal_7 |
| 426 | lh.inferiorparietal_10 | rh.precentral_35 | rh.paracentral_2 | lh.caudalmiddlefrontal_8 | lh.lateraloccipital_5 | rh.precuneus_23 | lh.postcentral_30 |
| 427 | rh.superiorparietal_21 | rh.caudalmiddlefrontal_7 | lh.supramarginal_18 | rh.precentral_32 | rh.caudalanteriorcingulate_6 | rh.precuneus_12 | rh.precuneus_13 |
| 428 | rh.bankssts_4 | lh.superiorparietal_10 | rh.precentral_32 | lh.caudalanteriorcingulate_4 | rh.precentral_24 | lh.supramarginal_21 | rh.precentral_10 |
| 429 | rh.superiortemporal_16 | lh.parsopercularis_1 | rh.precuneus_9 | rh.precentral_5 | lh.precuneus_5 | lh.rostralmiddlefrontal_24 | Right-Accumbens-area |
| 430 | lh.rostralmiddlefrontal_6 | lh.superiorfrontal_18 | rh.middletemporal_16 | lh.parsopercularis_7 | lh.parahippocampal_5 | lh.rostralmiddlefrontal_16 | rh.superiorparietal_28 |
| 431 | lh.postcentral_14 | lh.superiorfrontal_25 | lh.bankssts_6 | lh.inferiorparietal_9 | rh.inferiortemporal_15 | rh.superiorfrontal_22 | lh.parsopercularis_2 |
| 432 | lh.middletemporal_1 | rh.precentral_24 | rh.superiorfrontal_14 | rh.inferiorparietal_16 | rh.precentral_32 | lh.caudalmiddlefrontal_6 | lh.supramarginal_17 |
| 433 | rh.supramarginal_16 | rh.inferiorparietal_2 | lh.superiorparietal_18 | lh.inferiortemporal_3 | lh.precuneus_2 | rh.precuneus_11 | rh.lateraloccipital_5 |
| 434 | rh.superiorparietal_16 | lh.precentral_5 | rh.isthmuscingulate_2 | rh.fusiform_8 | rh.parstriangularis_6 | rh.precuneus_15 | lh.supramarginal_4 |
| 435 | rh.pericalcarine_1 | rh.rostralmiddlefrontal_4 | rh.fusiform_15 | rh.postcentral_14 | rh.rostralmiddlefrontal_15 | rh.lateraloccipital_7 | lh.superiorfrontal_45 |
| 436 | rh.lingual_7 | lh.superiorparietal_18 | lh.postcentral_15 | rh.superiorparietal_1 | lh.inferiorparietal_3 | lh.inferiorparietal_1 | rh.supramarginal_13 |
| 437 | lh.superiorfrontal_35 | rh.supramarginal_6 | rh.superiorfrontal_16 | rh.postcentral_8 | lh.precuneus_16 | rh.inferiorparietal_9 | rh.rostralanteriorcingulate_1 |
| 438 | lh.fusiform_16 | lh.precuneus_10 | lh.postcentral_30 | lh.superiorfrontal_33 | lh.superiorfrontal_25 | rh.middletemporal_19 | rh.precentral_22 |
| 439 | rh.parahippocampal_2 | lh.precentral_32 | lh.supramarginal_5 | rh.lateraloccipital_14 | lh.supramarginal_13 | lh.medialorbitofrontal_2 | lh.precentral_21 |
| 440 | rh.rostralmiddlefrontal_7 | lh.postcentral_18 | lh.superiorfrontal_24 | lh.superiorparietal_18 | rh.rostralanteriorcingulate_4 | rh.superiorparietal_7 | rh.caudalanteriorcingulate_6 |
| 441 | rh.superiorparietal_26 | rh.precuneus_18 | rh.precentral_36 | lh.supramarginal_6 | lh.precentral_35 | lh.fusiform_11 | lh.superiorfrontal_26 |
| 442 | rh.lateraloccipital_2 | rh.superiorparietal_2 | rh.postcentral_6 | rh.superiorfrontal_8 | rh.rostralmiddlefrontal_27 | lh.inferiorparietal_4 | lh.postcentral_10 |
| 443 | lh.precentral_3 | rh.lateraloccipital_4 | lh.fusiform_6 | lh.postcentral_30 | lh.postcentral_12 | lh.parsopercularis_2 | rh.insula_3 |
| 444 | lh.supramarginal_5 | lh.precentral_19 | lh.precentral_35 | rh.fusiform_5 | lh.parsopercularis_5 | lh.supramarginal_4 | rh.posteriorcingulate_9 |
| 445 | rh.rostralmiddlefrontal_15 | lh.rostralmiddlefrontal_23 | lh.superiorfrontal_40 | lh.rostralmiddlefrontal_26 | rh.precentral_11 | rh.precentral_30 | rh.precuneus_5 |
| 446 | rh.superiorfrontal_34 | rh.superiorfrontal_33 | lh.superiorfrontal_18 | lh.precuneus_2 | lh.inferiorparietal_8 | lh.rostralmiddlefrontal_22 | lh.insula_16 |
| 447 | lh.insula_10 | rh.rostralmiddlefrontal_27 | lh.insula_3 | lh.superiorfrontal_36 | rh.paracentral_12 | rh.postcentral_8 | rh.precentral_8 |
| 448 | rh.precentral_24 | lh.rostralmiddlefrontal_10 | rh.precentral_8 | lh.lingual_4 | lh.superiorfrontal_17 | rh.inferiorparietal_18 | rh.superiorfrontal_5 |
| 449 | lh.precuneus_2 | lh.inferiortemporal_3 | rh.inferiorparietal_9 | rh.transversetemporal_3 | rh.superiorparietal_17 | rh.paracentral_5 | rh.postcentral_27 |
| 450 | lh.lateraloccipital_3 | rh.superiorfrontal_6 | lh.cuneus_6 | lh.caudalanteriorcingulate_5 | rh.supramarginal_4 | rh.caudalanteriorcingulate_3 | rh.posteriorcingulate_4 |
| 451 | rh.paracentral_4 | rh.superiorfrontal_21 | rh.inferiorparietal_10 | rh.precentral_11 | lh.bankssts_6 | rh.lateraloccipital_23 | rh.inferiortemporal_16 |
| 452 | rh.postcentral_6 | lh.transversetemporal_2 | rh.insula_15 | lh.parsopercularis_4 | lh.lateraloccipital_21 | rh.inferiorparietal_4 | lh.lateraloccipital_18 |
| 453 | rh.superiorparietal_3 | lh.superiorparietal_15 | rh.middletemporal_15 | lh.precentral_11 | lh.inferiorparietal_11 | lh.middletemporal_6 | lh.inferiorparietal_14 |
| 454 | rh.bankssts_6 | rh.superiorfrontal_5 | rh.postcentral_8 | rh.inferiorparietal_3 | lh.postcentral_5 | lh.precuneus_15 | rh.paracentral_6 |
| 455 | lh.superiorparietal_21 | lh.precuneus_2 | rh.precentral_24 | lh.cuneus_3 | lh.inferiortemporal_16 | lh.precentral_16 | lh.supramarginal_11 |
| 456 | lh.precuneus_7 | lh.superiorfrontal_6 | rh.precuneus_2 | lh.rostralmiddlefrontal_22 | lh.precuneus_1 | rh.precentral_19 | rh.supramarginal_14 |
| 457 | lh.fusiform_10 | rh.precentral_30 | lh.parsopercularis_9 | rh.superiorparietal_2 | rh.caudalmiddlefrontal_2 | rh.parstriangularis_6 | lh.precentral_11 |
| 458 | rh.fusiform_2 | lh.lingual_17 | rh.lingual_16 | lh.posteriorcingulate_7 | lh.lateralorbitofrontal_5 | lh.lingual_6 | lh.superiorfrontal_43 |

| | | | | | | | |
|---|---|---|---|---|---|---|---|
| 459 | lh.lateralorbitofrontal_10 | lh.postcentral_8 | rh.postcentral_14 | lh.inferiorparietal_7 | lh.precentral_17 | rh.posteriorcingulate_3 | lh.postcentral_31 |
| 460 | lh.superiorfrontal_21 | rh.superiorparietal_15 | lh.superiorfrontal_9 | rh.precuneus_18 | lh.precuneus_7 | lh.insula_17 | rh.postcentral_11 |
| 461 | lh.rostralanteriorcingulate_3 | lh.rostralanteriorcingulate_5 | lh.lingual_5 | lh.caudalmiddlefrontal_7 | rh.superiorfrontal_19 | rh.paracentral_7 | lh.superiorfrontal_28 |
| 462 | lh.inferiortemporal_3 | rh.cuneus_6 | lh.supramarginal_6 | lh.postcentral_18 | rh.precuneus_21 | lh.supramarginal_6 | lh.bankssts_4 |
| 463 | rh.precentral_13 | rh.precentral_34 | lh.caudalmiddlefrontal_2 | lh.precentral_32 | rh.precuneus_2 | rh.lateraloccipital_13 | lh.precentral_7 |
| 464 | rh.middletemporal_15 | lh.superiorfrontal_19 | lh.inferiorparietal_9 | lh.isthmuscingulate_1 | rh.precuneus_14 | rh.precentral_7 | rh.superiorfrontal_8 |
| 465 | rh.precentral_11 | lh.superiorparietal_22 | rh.parsopercularis_5 | rh.superiorfrontal_15 | lh.postcentral_14 | rh.fusiform_16 | lh.inferiorparietal_16 |
| 466 | rh.supramarginal_4 | rh.precentral_11 | rh.caudalmiddlefrontal_3 | rh.postcentral_9 | Left-Amygdala | lh.rostralmiddlefrontal_11 | rh.supramarginal_9 |
| 467 | rh.parsopercularis_4 | rh.caudalanteriorcingulate_1 | rh.cuneus_8 | lh.precentral_5 | lh.supramarginal_5 | rh.precuneus_21 | lh.precuneus_7 |
| 468 | lh.precentral_29 | lh.rostralmiddlefrontal_15 | lh.superiorfrontal_19 | lh.postcentral_13 | rh.fusiform_15 | rh.precentral_16 | lh.isthmuscingulate_1 |
| 469 | lh.paracentral_9 | rh.middletemporal_4 | rh.inferiorparietal_1 | rh.parsopercularis_5 | rh.postcentral_6 | rh.rostralmiddlefrontal_15 | rh.precentral_27 |
| 470 | lh.inferiorparietal_19 | rh.superiortemporal_21 | rh.caudalmiddlefrontal_7 | rh.rostralmiddlefrontal_22 | rh.precuneus_13 | lh.parsopercularis_8 | lh.supramarginal_19 |
| 471 | lh.precuneus_1 | rh.precentral_5 | rh.superiorfrontal_32 | lh.fusiform_1 | rh.lateraloccipital_7 | Left-Caudate | lh.bankssts_2 |
| 472 | lh.inferiortemporal_12 | rh.cuneus_8 | lh.lingual_4 | lh.rostralanteriorcingulate_5 | lh.rostralmiddlefrontal_3 | lh.superiorfrontal_17 | rh.precentral_5 |
| 473 | rh.lateraloccipital_6 | rh.superiorfrontal_34 | lh.superiorfrontal_35 | rh.caudalmiddlefrontal_7 | lh.supramarginal_10 | rh.posteriorcingulate_9 | lh.superiorfrontal_36 |
| 474 | lh.superiorfrontal_32 | lh.supramarginal_13 | rh.rostralmiddlefrontal_2 | rh.lingual_14 | rh.superiorparietal_28 | lh.lateralorbitofrontal_11 | rh.superiorfrontal_33 |
| 475 | lh.caudalmiddlefrontal_12 | rh.precentral_8 | lh.lateraloccipital_22 | lh.inferiortemporal_12 | lh.rostralanteriorcingulate_1 | rh.superiorparietal_21 | rh.inferiorparietal_10 |
| 476 | lh.superiorparietal_26 | lh.inferiortemporal_12 | lh.precentral_2 | lh.precuneus_16 | lh.rostralmiddlefrontal_7 | lh.posteriorcingulate_2 | lh.superiorparietal_2 |
| 477 | lh.precuneus_16 | lh.precuneus_16 | rh.superiorparietal_12 | rh.insula_8 | rh.superiorfrontal_37 | lh.precuneus_3 | lh.insula_17 |
| 478 | rh.precuneus_22 | lh.lateraloccipital_9 | lh.supramarginal_13 | lh.lingual_17 | lh.rostralanteriorcingulate_4 | lh.insula_2 | rh.parstriangularis_4 |
| 479 | rh.precuneus_13 | lh.supramarginal_5 | rh.cuneus_5 | lh.precentral_13 | rh.supramarginal_16 | lh.superiorfrontal_28 | lh.inferiorparietal_17 |
| 480 | rh.lingual_3 | rh.caudalmiddlefrontal_4 | rh.inferiorparietal_2 | lh.parsopercularis_1 | rh.bankssts_6 | lh.inferiorparietal_10 | rh.postcentral_26 |
| 481 | lh.postcentral_3 | rh.insula_7 | rh.lateraloccipital_4 | lh.superiorparietal_10 | lh.lingual_17 | lh.isthmuscingulate_4 | rh.superiorparietal_7 |
| 482 | lh.inferiorparietal_8 | rh.fusiform_8 | rh.lateralorbitofrontal_6 | rh.superiorfrontal_12 | lh.insula_1 | Right-Caudate | lh.bankssts_5 |
| 483 | lh.lingual_12 | lh.middletemporal_1 | rh.postcentral_11 | rh.superiorfrontal_34 | rh.middletemporal_4 | rh.lingual_7 | rh.precentral_31 |
| 484 | rh.precuneus_15 | lh.precuneus_21 | lh.precentral_29 | lh.rostralmiddlefrontal_23 | rh.middletemporal_15 | rh.lateraloccipital_7 | lh.superiortemporal_24 |
| 485 | lh.precuneus_5 | rh.precentral_13 | rh.lateraloccipital_10 | lh.inferiorparietal_22 | rh.parsopercularis_3 | lh.inferiorparietal_11 | lh.superiorparietal_14 |
| 486 | lh.lingual_6 | lh.bankssts_3 | rh.insula_2 | rh.fusiform_13 | lh.superiorparietal_26 | rh.parsopercularis_7 | Brain-Stem |
| 487 | lh.fusiform_7 | rh.postcentral_13 | lh.inferiortemporal_3 | rh.insula_4 | rh.postcentral_5 | rh.insula_5 | lh.superiorfrontal_39 |
| 488 | lh.middletemporal_10 | lh.inferiorparietal_4 | rh.lingual_7 | lh.precuneus_10 | lh.superiorfrontal_24 | rh.superiorparietal_3 | lh.postcentral_21 |
| 489 | rh.superiorfrontal_35 | lh.lingual_5 | lh.superiorfrontal_13 | rh.isthmuscingulate_2 | rh.superiorfrontal_3 | rh.precentral_25 | lh.superiorfrontal_15 |
| 490 | rh.superiorparietal_28 | lh.supramarginal_19 | lh.superiorfrontal_10 | rh.inferiortemporal_14 | lh.superiorfrontal_14 | rh.caudalmiddlefrontal_7 | lh.supramarginal_7 |
| 491 | lh.superiorparietal_4 | rh.superiorfrontal_35 | lh.precentral_32 | lh.superiorfrontal_5 | lh.supramarginal_20 | rh.lateralorbitofrontal_6 | rh.middletemporal_13 |
| 492 | lh.lateraloccipital_7 | rh.lateraloccipital_10 | rh.middletemporal_4 | lh.supramarginal_5 | lh.precentral_29 | rh.insula_4 | lh.superiorparietal_8 |
| 493 | lh.middletemporal_14 | rh.fusiform_13 | rh.cuneus_7 | rh.precentral_21 | rh.lateraloccipital_6 | lh.parstriangularis_1 | rh.superiorfrontal_15 |
| 494 | rh.lingual_17 | rh.lingual_7 | rh.rostralanteriorcingulate_4 | rh.superiorfrontal_35 | Right-Accumbens-area | Left-Pallidum | rh.postcentral_6 |
| 495 | lh.paracentral_1 | rh.inferiorparietal_4 | lh.lateraloccipital_2 | rh.precentral_13 | lh.superiorfrontal_13 | lh.insula_8 | lh.superiorparietal_26 |
| 496 | lh.superiorparietal_12 | lh.superiorfrontal_4 | lh.lateraloccipital_8 | lh.posteriorcingulate_3 | rh.lateraloccipital_2 | lh.superiorfrontal_45 | rh.supramarginal_1 |
| 497 | lh.inferiorparietal_11 | lh.superiorparietal_19 | lh.rostralmiddlefrontal_10 | lh.superiorfrontal_9 | lh.middletemporal_1 | lh.caudalmiddlefrontal_11 | rh.superiorfrontal_10 |
| 498 | lh.lateraloccipital_22 | lh.superiorfrontal_17 | lh.superiorfrontal_33 | rh.middletemporal_4 | lh.superiortemporal_9 | rh.parsopercularis_5 | lh.precentral_5 |
| 499 | rh.precuneus_12 | rh.postcentral_2 | lh.precentral_12 | lh.superiorfrontal_17 | rh.fusiform_14 | rh.superiorfrontal_12 | rh.rostralanteriorcingulate_4 |
| 500 | lh.parsopercularis_5 | lh.lateraloccipital_20 | lh.superiorparietal_17 | lh.supramarginal_13 | lh.superiortemporal_20 | lh.insula_7 | lh.superiorfrontal_5 |
| 501 | lh.superiorfrontal_23 | rh.parstriangularis_3 | lh.superiorfrontal_30 | rh.caudalanteriorcingulate_4 | lh.inferiorparietal_19 | rh.paracentral_11 | lh.paracentral_8 |
| 502 | lh.fusiform_6 | rh.inferiortemporal_14 | rh.superiorfrontal_20 | rh.parstriangularis_8 | lh.middletemporal_10 | rh.middletemporal_18 | rh.lateraloccipital_6 |
| 503 | lh.superiortemporal_25 | rh.superiorfrontal_36 | rh.precentral_3 | lh.superiorparietal_22 | lh.superiorfrontal_10 | lh.lingual_12 | rh.lateralorbitofrontal_8 |
| 504 | Left-Amygdala | rh.inferiorparietal_11 | lh.precuneus_2 | rh.precuneus_5 | rh.superiorfrontal_16 | lh.caudalanteriorcingulate_2 | lh.medialorbitofrontal_5 |

| | | | | | | | |
|---|---|---|---|---|---|---|---|
| 505 | lh.supramarginal_10 | lh.supramarginal_10 | rh.postcentral_3 | lh.middletemporal_1 | rh.superiorparietal_21 | rh.caudalmiddlefrontal_4 | rh.lateralorbitofrontal_14 |
| 506 | lh.postcentral_19 | rh.cuneus_5 | lh.insula_17 | lh.postcentral_15 | rh.superiortemporal_16 | lh.postcentral_28 | rh.bankssts_5 |
| 507 | lh.transversetemporal_3 | lh.parsopercularis_9 | rh.superiorfrontal_35 | lh.superiorfrontal_10 | lh.lateralorbitofrontal_10 | rh.parsopercularis_3 | lh.supramarginal_13 |
| 508 | rh.parstriangularis_6 | lh.parahippocampal_5 | lh.lateraloccipital_20 | lh.superiorfrontal_15 | lh.pericalcarine_6 | lh.caudalmiddlefrontal_7 | lh.supramarginal_8 |
| 509 | rh.caudalmiddlefrontal_2 | lh.superiorfrontal_36 | lh.superiorfrontal_5 | lh.supramarginal_10 | rh.lateraloccipital_22 | rh.insula_8 | lh.postcentral_19 |
| 510 | rh.superiorparietal_17 | rh.superiorparietal_21 | rh.superiorparietal_26 | lh.superiorparietal_15 | rh.precentral_25 | lh.parsorbitalis_1 | rh.middletemporal_15 |
| 511 | rh.precuneus_14 | rh.rostralmiddlefrontal_2 | rh.rostralmiddlefrontal_13 | rh.precentral_8 | lh.superiorfrontal_37 | rh.supramarginal_13 | lh.precentral_14 |
| 512 | rh.fusiform_13 | rh.precentral_21 | lh.postcentral_18 | lh.rostralmiddlefrontal_21 | rh.rostralmiddlefrontal_13 | rh.superiorfrontal_41 | lh.middletemporal_10 |
| 513 | rh.superiortemporal_11 | lh.pericalcarine_1 | lh.superiortemporal_9 | rh.superiorfrontal_32 | rh.superiorfrontal_18 | lh.superiorparietal_5 | lh.precentral_29 |
| 514 | lh.rostralmiddlefrontal_21 | lh.rostralmiddlefrontal_21 | rh.inferiorparietal_11 | lh.transversetemporal_2 | lh.lateralorbitofrontal_8 | lh.parsopercularis_10 | rh.postcentral_9 |
| 515 | lh.superiorfrontal_17 | lh.middletemporal_10 | rh.caudalmiddlefrontal_4 | rh.lingual_7 | rh.insula_3 | rh.inferiortemporal_9 | rh.superiorfrontal_30 |
| 516 | lh.precentral_19 | lh.inferiorparietal_10 | lh.insula_10 | rh.paracentral_6 | lh.lateraloccipital_3 | rh.middletemporal_13 | rh.precuneus_3 |
| 517 | lh.rostralanteriorcingulate_1 | lh.parsopercularis_3 | lh.superiorparietal_10 | lh.middletemporal_10 | lh.postcentral_19 | rh.pericalcarine_8 | lh.inferiorparietal_6 |
| 518 | lh.supramarginal_9 | rh.parahippocampal_2 | lh.rostralmiddlefrontal_21 | rh.lingual_1 | rh.inferiorparietal_5 | rh.superiortemporal_11 | lh.postcentral_5 |
| 519 | lh.precuneus_19 | lh.lateraloccipital_2 | lh.superiorfrontal_17 | lh.postcentral_8 | lh.superiorfrontal_7 | rh.middletemporal_9 | lh.temporalpole_2 |
| 520 | lh.lateraloccipital_17 | lh.precentral_29 | lh.fusiform_5 | rh.superiortemporal_21 | rh.rostralanteriorcingulate_2 | rh.paracentral_3 | lh.supramarginal_5 |
| 521 | rh.precentral_14 | lh.postcentral_9 | rh.postcentral_9 | rh.cuneus_1 | lh.inferiortemporal_12 | rh.medialorbitofrontal_6 | rh.precentral_21 |
| 522 | rh.paracentral_12 | lh.superiorparietal_17 | rh.precentral_11 | lh.superiorfrontal_13 | lh.parsopercularis_10 | rh.insula_13 | lh.inferiortemporal_15 |
| 523 | Right-Accumbens-area | lh.cuneus_4 | rh.rostralmiddlefrontal_4 | lh.superiorfrontal_12 | lh.supramarginal_9 | lh.precentral_34 | lh.superiorfrontal_6 |
| 524 | lh.fusiform_12 | rh.pericalcarine_3 | lh.inferiortemporal_12 | rh.superiorparietal_15 | lh.lateraloccipital_17 | rh.superiorfrontal_24 | rh.superiorfrontal_4 |
| 525 | lh.rostralmiddlefrontal_10 | rh.superiorparietal_26 | rh.precuneus_14 | lh.pericalcarine_7 | rh.middletemporal_13 | rh.insula_7 | rh.precentral_32 |
| 526 | lh.insula_1 | lh.precentral_20 | lh.superiorparietal_4 | rh.superiorfrontal_20 | rh.bankssts_4 | lh.temporalpole_2 | lh.supramarginal_9 |
| 527 | lh.fusiform_8 | rh.supramarginal_14 | rh.superiorfrontal_15 | rh.rostralmiddlefrontal_13 | lh.precuneus_19 | lh.bankssts_5 | lh.lateraloccipital_17 |
| 528 | rh.rostralmiddlefrontal_4 | rh.fusiform_5 | lh.inferiorparietal_22 | lh.fusiform_5 | rh.precuneus_15 | lh.precentral_30 | lh.superiorfrontal_34 |
| 529 | rh.supramarginal_2 | lh.precuneus_7 | rh.insula_12 | lh.precentral_29 | lh.inferiortemporal_3 | rh.middletemporal_16 | rh.lateraloccipital_17 |
| 530 | rh.parahippocampal_3 | rh.superiorparietal_1 | rh.superiorparietal_16 | rh.precentral_34 | Left-Accumbens-area | rh.precuneus_13 | lh.precuneus_19 |
| 531 | rh.middletemporal_13 | lh.fusiform_1 | rh.precentral_13 | lh.inferiorparietal_4 | rh.rostralanteriorcingulate_1 | rh.insula_14 | rh.bankssts_6 |
| 532 | lh.precentral_14 | lh.postcentral_14 | lh.supramarginal_10 | lh.lateraloccipital_14 | lh.supramarginal_11 | rh.rostralmiddlefrontal_16 | lh.precentral_24 |
| 533 | lh.fusiform_17 | Right-Amygdala | lh.superiorfrontal_4 | rh.superiorparietal_21 | rh.insula_2 | lh.inferiorparietal_6 | lh.postcentral_13 |
| 534 | lh.middletemporal_3 | lh.inferiorparietal_13 | rh.supramarginal_14 | rh.postcentral_2 | lh.precentral_14 | rh.caudalmiddlefrontal_2 | rh.superiorparietal_8 |
| 535 | rh.superiorfrontal_16 | rh.posteriorcingulate_4 | rh.lateraloccipital_14 | lh.rostralmiddlefrontal_15 | lh.superiorfrontal_45 | lh.postcentral_31 | lh.inferiorparietal_19 |
| 536 | lh.middletemporal_13 | lh.superiorfrontal_10 | lh.middletemporal_1 | lh.supramarginal_19 | lh.fusiform_16 | rh.paracentral_4 | lh.supramarginal_1 |
| 537 | rh.insula_3 | rh.cuneus_1 | lh.parsopercularis_3 | rh.parahippocampal_2 | rh.parahippocampal_2 | lh.rostralmiddlefrontal_2 | rh.postcentral_14 |
| 538 | lh.superiorfrontal_25 | lh.superiorfrontal_13 | lh.inferiorparietal_13 | rh.cuneus_7 | rh.fusiform_2 | lh.supramarginal_12 | rh.superiorfrontal_9 |
| 539 | rh.inferiortemporal_14 | rh.superiorfrontal_8 | lh.precentral_22 | rh.postcentral_3 | lh.precentral_24 | lh.superiortemporal_7 | rh.temporalpole_2 |
| 540 | lh.pericalcarine_5 | rh.precuneus_15 | lh.inferiorparietal_8 | lh.inferiorparietal_10 | rh.superiorparietal_3 | lh.entorhinal_3 | rh.parstriangularis_7 |
| 541 | rh.superiorfrontal_3 | lh.lateraloccipital_22 | lh.superiorfrontal_6 | rh.rostralmiddlefrontal_2 | rh.precentral_30 | lh.superiorfrontal_7 | rh.postcentral_30 |
| 542 | rh.inferiorparietal_5 | lh.fusiform_5 | rh.parahippocampal_2 | lh.precentral_2 | lh.lateralorbitofrontal_1 | lh.posteriorcingulate_8 | lh.postcentral_14 |
| 543 | rh.lateraloccipital_23 | lh.postcentral_12 | lh.bankssts_5 | lh.precuneus_7 | lh.superiorfrontal_28 | Right-Amygdala | rh.superiorfrontal_32 |
| 544 | lh.superiorfrontal_7 | rh.lateraloccipital_14 | lh.superiorfrontal_36 | lh.precuneus_21 | rh.postcentral_7 | rh.insula_6 | rh.precentral_6 |
| 545 | lh.precentral_24 | rh.paracentral_6 | rh.inferiorparietal_4 | rh.superiorparietal_26 | rh.superiorfrontal_6 | rh.superiorfrontal_33 | lh.postcentral_9 |
| 546 | rh.rostralmiddlefrontal_3 | lh.superiorfrontal_30 | lh.precuneus_10 | rh.inferiorparietal_2 | rh.lateraloccipital_17 | lh.lateraloccipital_22 | rh.superiorfrontal_1 |
| 547 | lh.superiorfrontal_37 | rh.lingual_1 | lh.precentral_13 | lh.parahippocampal_5 | rh.superiorparietal_29 | rh.superiorfrontal_21 | lh.middletemporal_12 |
| 548 | lh.precentral_17 | rh.superiorparietal_16 | rh.precentral_21 | rh.caudalmiddlefrontal_4 | lh.fusiform_10 | rh.superiorfrontal_18 | lh.inferiortemporal_7 |
| 549 | lh.cuneus_6 | rh.precuneus_14 | rh.superiorfrontal_8 | rh.lingual_2 | rh.precentral_5 | lh.superiorfrontal_34 | lh.middletemporal_6 |
| 550 | rh.superiorfrontal_18 | rh.insula_14 | rh.precentral_18 | lh.lateraloccipital_22 | lh.middletemporal_3 | rh.caudalanteriorcingulate_5 | lh.insula_12 |

| | | | | | | | |
|---|---|---|---|---|---|---|---|
| 551 | rh.rostralanteriorcingulate_1 | rh.inferiorparietal_7 | rh.precentral_34 | lh.paracentral_8 | lh.superiorfrontal_1 | rh.superiorfrontal_6 | rh.superiorfrontal_21 |
| 552 | rh.lateraloccipital_17 | rh.lingual_16 | rh.posteriorcingulate_4 | rh.inferiorparietal_4 | lh.middletemporal_13 | lh.superiorfrontal_13 | rh.precentral_18 |
| 553 | lh.rostralanteriorcingulate_4 | lh.superiorfrontal_15 | lh.inferiorparietal_7 | rh.precuneus_15 | rh.precentral_21 | rh.inferiortemporal_16 | rh.inferiortemporal_13 |
| 554 | lh.postcentral_25 | lh.middletemporal_14 | lh.caudalmiddlefrontal_7 | lh.postcentral_14 | rh.precuneus_12 | lh.caudalmiddlefrontal_2 | lh.lateraloccipital_23 |
| 555 | rh.supramarginal_11 | rh.bankssts_5 | lh.rostralmiddlefrontal_17 | rh.precuneus_14 | lh.lateraloccipital_22 | lh.postcentral_7 | rh.superiorfrontal_38 |
| 556 | lh.superiorparietal_5 | lh.superiorparietal_4 | rh.postcentral_2 | lh.lingual_9 | rh.parstriangularis_7 | rh.parsopercularis_1 | rh.bankssts_2 |
| 557 | lh.fusiform_3 | lh.insula_12 | rh.paracentral_6 | rh.precentral_18 | lh.lateraloccipital_7 | lh.parsopercularis_3 | lh.rostralmiddlefrontal_2 |
| 558 | rh.parstriangularis_7 | rh.lingual_2 | rh.precentral_5 | rh.superiorfrontal_7 | lh.superiorfrontal_19 | lh.paracentral_2 | lh.precuneus_15 |
| 559 | rh.parsopercularis_3 | rh.rostralmiddlefrontal_13 | rh.supramarginal_4 | lh.cuneus_5 | rh.inferiortemporal_14 | rh.superiorfrontal_39 | lh.parahippocampal_5 |
| 560 | rh.superiorfrontal_37 | rh.precuneus_13 | lh.postcentral_9 | rh.postcentral_13 | lh.postcentral_25 | rh.superiorfrontal_28 | lh.lateraloccipital_5 |
| 561 | rh.lingual_2 | rh.superiorparietal_12 | lh.superiorfrontal_14 | rh.lateraloccipital_13 | lh.medialorbitofrontal_4 | lh.precentral_28 | rh.precuneus_17 |
| 562 | rh.fusiform_4 | rh.superiorfrontal_15 | lh.superiorparietal_15 | lh.bankssts_3 | lh.cuneus_1 | rh.postcentral_10 | lh.inferiorparietal_3 |
| 563 | rh.precentral_21 | lh.bankssts_5 | lh.precuneus_7 | lh.precentral_22 | rh.fusiform_17 | rh.precuneus_17 | rh.precentral_16 |
| 564 | rh.fusiform_9 | lh.inferiorparietal_19 | lh.parahippocampal_5 | lh.lingual_3 | rh.superiorfrontal_33 | rh.posteriorcingulate_1 | lh.paracentral_2 |
| 565 | lh.superiorfrontal_45 | lh.superiorfrontal_40 | lh.paracentral_10 | lh.superiorparietal_19 | rh.fusiform_13 | lh.fusiform_13 | rh.middletemporal_18 |
| 566 | rh.rostralmiddlefrontal_27 | rh.supramarginal_4 | rh.superiorparietal_21 | rh.caudalanteriorcingulate_1 | rh.parahippocampal_3 | lh.superiorfrontal_30 | lh.postcentral_25 |
| 567 | rh.cuneus_1 | lh.superiortemporal_9 | lh.pericalcarine_1 | lh.middletemporal_14 | rh.superiortemporal_11 | lh.supramarginal_19 | lh.fusiform_18 |
| 568 | rh.superiortemporal_6 | lh.superiorfrontal_33 | lh.lateralorbitofrontal_10 | lh.superiorparietal_4 | rh.paracentral_6 | lh.posteriorcingulate_5 | rh.superiortemporal_14 |
| 569 | lh.rostralmiddlefrontal_7 | rh.postcentral_3 | lh.supramarginal_19 | rh.lateraloccipital_12 | lh.parsopercularis_2 | rh.insula_15 | lh.precentral_30 |
| 570 | lh.superiorfrontal_13 | lh.postcentral_15 | rh.lingual_1 | rh.precentral_31 | lh.superiorfrontal_6 | lh.superiorparietal_9 | rh.inferiortemporal_5 |
| 571 | Left-Accumbens-area | lh.superiorfrontal_9 | rh.postcentral_13 | rh.fusiform_6 | lh.middletemporal_14 | rh.postcentral_28 | rh.bankssts_3 |
| 572 | rh.fusiform_12 | lh.inferiorparietal_8 | rh.superiorparietal_1 | lh.precentral_10 | lh.fusiform_6 | lh.lateraloccipital_11 | lh.superiorfrontal_30 |
| 573 | rh.precentral_4 | Left-Amygdala | rh.fusiform_8 | lh.parsopercularis_9 | lh.inferiorparietal_14 | lh.caudalanteriorcingulate_3 | rh.lateraloccipital_7 |
| 574 | rh.precentral_25 | lh.lingual_9 | lh.inferiorparietal_19 | rh.precuneus_13 | lh.superiorparietal_28 | lh.superiorfrontal_36 | rh.supramarginal_16 |
| 575 | rh.superiorfrontal_24 | rh.insula_15 | lh.supramarginal_12 | lh.parsopercularis_3 | lh.precentral_7 | rh.superiorparietal_4 | rh.inferiortemporal_14 |
| 576 | lh.fusiform_5 | lh.cuneus_3 | lh.pericalcarine_7 | rh.pericalcarine_8 | lh.supramarginal_8 | rh.postcentral_30 | lh.postcentral_12 |
| 577 | rh.precentral_5 | rh.caudalanteriorcingulate_6 | rh.precentral_25 | Right-Amygdala | rh.supramarginal_2 | lh.fusiform_18 | lh.rostralmiddlefrontal_24 |
| 578 | lh.superiorfrontal_28 | lh.precuneus_5 | lh.precentral_10 | lh.inferiorparietal_13 | lh.fusiform_7 | lh.lateralorbitofrontal_15 | rh.supramarginal_15 |
| 579 | rh.lateraloccipital_15 | lh.superiorfrontal_5 | lh.inferiorparietal_10 | lh.superiorparietal_9 | rh.precuneus_22 | lh.postcentral_2 | rh.middletemporal_13 |
| 580 | rh.inferiortemporal_6 | lh.supramarginal_12 | lh.precentral_20 | rh.superiorparietal_16 | lh.transversetemporal_3 | rh.inferiortemporal_13 | rh.precuneus_15 |
| 581 | lh.parsopercularis_10 | rh.precentral_10 | rh.insula_3 | lh.postcentral_9 | lh.postcentral_3 | lh.paracentral_8 | lh.bankssts_3 |
| 582 | lh.middletemporal_7 | rh.precuneus_21 | lh.insula_1 | rh.postcentral_20 | lh.superiortemporal_25 | rh.superiorparietal_6 | lh.rostralmiddlefrontal_12 |
| 583 | rh.precentral_30 | rh.insula_9 | lh.inferiorparietal_11 | rh.parstriangularis_3 | rh.superiorfrontal_30 | lh.superiorfrontal_37 | lh.middletemporal_3 |
| 584 | rh.lateraloccipital_11 | lh.precentral_17 | rh.lingual_14 | rh.supramarginal_14 | rh.pericalcarine_1 | lh.insula_3 | rh.inferiortemporal_12 |
| 585 | rh.fusiform_8 | lh.inferiorparietal_11 | lh.cuneus_3 | lh.precentral_17 | lh.superiorfrontal_8 | rh.paracentral_6 | lh.bankssts_6 |
| 586 | lh.superiorfrontal_6 | rh.inferiorparietal_26 | rh.fusiform_13 | lh.inferiorparietal_19 | rh.lingual_17 | rh.precentral_36 | lh.inferiortemporal_12 |
| 587 | rh.transversetemporal_2 | rh.superiortemporal_16 | rh.superiorparietal_15 | rh.insula_7 | rh.superiorfrontal_5 | lh.middletemporal_12 | lh.lateralorbitofrontal_10 |
| 588 | lh.rostralmiddlefrontal_24 | rh.precentral_18 | rh.fusiform_5 | rh.precentral_25 | rh.precentral_4 | lh.postcentral_13 | lh.precentral_10 |
| 589 | lh.inferiortemporal_4 | lh.precuneus_1 | rh.superiorfrontal_9 | lh.superiorfrontal_14 | lh.middletemporal_7 | lh.superiorfrontal_27 | lh.superiorfrontal_33 |
| 590 | lh.middletemporal_16 | rh.precentral_25 | lh.superiorfrontal_15 | rh.supramarginal_4 | lh.fusiform_17 | rh.precentral_11 | lh.middletemporal_7 |
| 591 | rh.superiorfrontal_19 | rh.rostralanteriorcingulate_3 | rh.precentral_35 | rh.inferiorparietal_11 | lh.rostralmiddlefrontal_24 | lh.superiorfrontal_26 | rh.superiorfrontal_40 |
| 592 | rh.pericalcarine_5 | rh.rostralmiddlefrontal_7 | lh.middletemporal_14 | rh.pericalcarine_6 | rh.lateraloccipital_11 | lh.posteriorcingulate_4 | rh.superiorparietal_20 |
| 593 | rh.superiorfrontal_33 | rh.superiorparietal_28 | lh.precuneus_21 | lh.postcentral_12 | lh.lingual_12 | lh.parsorbitalis_2 | lh.supramarginal_12 |
| 594 | rh.rostralanteriorcingulate_2 | rh.supramarginal_20 | lh.insula_11 | Left-Amygdala | rh.precentral_6 | rh.superiorparietal_23 | lh.precuneus_14 |
| 595 | lh.supramarginal_8 | lh.lingual_4 | lh.superiorparietal_22 | lh.precentral_20 | rh.fusiform_4 | lh.parsorbitalis_3 | lh.precentral_35 |
| 596 | lh.pericalcarine_3 | lh.parstriangularis_6 | lh.precuneus_5 | rh.superiorfrontal_9 | rh.fusiform_9 | rh.precentral_32 | rh.lateraloccipital_11 |

| | | | | | | | |
|---|---|---|---|---|---|---|---|
| 597 | lh.inferiortemporal_10 | lh.superiorparietal_21 | lh.postcentral_8 | lh.inferiorparietal_8 | lh.fusiform_8 | rh.superiorfrontal_17 | rh.temporalpole_3 |
| 598 | rh.superiorparietal_29 | rh.superiorfrontal_32 | lh.bankssts_3 | rh.inferiortemporal_6 | lh.lingual_6 | lh.lateralorbitofrontal_6 | lh.postcentral_8 |
| 599 | rh.superiorparietal_6 | rh.inferiortemporal_6 | rh.cuneus_1 | rh.postcentral_25 | lh.superiorparietal_8 | lh.supramarginal_9 | lh.superiortemporal_18 |
| 600 | rh.superiorfrontal_30 | lh.precentral_10 | lh.cuneus_5 | lh.lateraloccipital_15 | lh.superiorfrontal_11 | rh.inferiortemporal_14 | lh.postcentral_23 |
| 601 | rh.pericalcarine_2 | lh.precentral_2 | lh.rostralmiddlefrontal_6 | lh.precuneus_5 | rh.lingual_7 | rh.lateraloccipital_21 | lh.superiorparietal_1 |
| 602 | rh.lingual_1 | lh.precentral_22 | lh.paracentral_8 | lh.fusiform_4 | lh.superiorfrontal_36 | rh.supramarginal_3 | rh.lateraloccipital_2 |
| 603 | lh.superiorparietal_7 | rh.superiorfrontal_12 | lh.superiorparietal_19 | rh.lateraloccipital_21 | lh.rostralmiddlefrontal_14 | rh.medialorbitofrontal_3 | rh.postcentral_3 |
| 604 | lh.lingual_9 | rh.rostralmiddlefrontal_16 | rh.inferiortemporal_14 | rh.inferiorparietal_7 | rh.insula_12 | lh.parsopercularis_5 | rh.inferiortemporal_15 |
| 605 | lh.inferiortemporal_1 | rh.precentral_31 | lh.postcentral_14 | rh.superiorparietal_12 | rh.superiorparietal_7 | lh.precentral_2 | rh.postcentral_13 |
| 606 | rh.paracentral_6 | lh.insula_7 | rh.cuneus_3 | rh.cuneus_4 | rh.precentral_31 | lh.postcentral_25 | lh.inferiortemporal_16 |
| 607 | lh.postcentral_26 | lh.superiorfrontal_14 | lh.fusiform_1 | rh.precentral_2 | rh.lateraloccipital_15 | rh.superiorfrontal_34 | lh.superiorfrontal_18 |
| 608 | lh.fusiform_15 | lh.middletemporal_13 | rh.parahippocampal_3 | lh.inferiorparietal_11 | lh.superiorfrontal_4 | rh.superiorfrontal_31 | rh.medialorbitofrontal_11 |
| 609 | rh.precentral_6 | lh.rostralmiddlefrontal_17 | lh.lingual_9 | lh.superiortemporal_9 | rh.lateraloccipital_23 | rh.superiorfrontal_40 | lh.precentral_22 |
| 610 | lh.lateraloccipital_16 | rh.parsopercularis_1 | lh.rostralanteriorcingulate_3 | rh.caudalanteriorcingulate_6 | rh.inferiortemporal_6 | rh.insula_3 | lh.postcentral_2 |
| 611 | lh.lingual_14 | lh.rostralanteriorcingulate_2 | lh.precentral_24 | lh.inferiortemporal_4 | rh.superiortemporal_6 | rh.superiorfrontal_13 | rh.precentral_4 |
| 612 | rh.superiorfrontal_6 | lh.cuneus_5 | lh.superiorparietal_9 | lh.precuneus_1 | lh.inferiortemporal_10 | rh.precentral_28 | rh.lingual_6 |
| 613 | rh.supramarginal_18 | rh.bankssts_6 | lh.parstriangularis_6 | rh.superiorparietal_28 | rh.fusiform_12 | rh.supramarginal_20 | rh.superiortemporal_21 |
| 614 | lh.parsopercularis_2 | lh.superiorparietal_5 | rh.inferiorparietal_26 | rh.insula_14 | rh.postcentral_3 | rh.temporalpole_3 | rh.medialorbitofrontal_10 |
| 615 | lh.superiorfrontal_10 | lh.rostralmiddlefrontal_6 | rh.postcentral_25 | lh.middletemporal_13 | lh.fusiform_3 | rh.middletemporal_12 | rh.postcentral_5 |
| 616 | lh.superiorfrontal_1 | lh.superiorparietal_12 | rh.supramarginal_20 | lh.superiorparietal_28 | rh.superiorfrontal_38 | rh.supramarginal_5 | rh.inferiortemporal_9 |
| 617 | lh.precentral_7 | lh.precuneus_19 | lh.postcentral_2 | rh.cuneus_3 | lh.superiorfrontal_15 | lh.superiorfrontal_43 | lh.parstriangularis_1 |
| 618 | lh.inferiorparietal_14 | lh.inferiortemporal_4 | rh.precentral_10 | lh.superiorparietal_5 | lh.precentral_10 | lh.parstriangularis_3 | lh.lateraloccipital_21 |
| 619 | lh.fusiform_9 | rh.lateralorbitofrontal_6 | lh.supramarginal_20 | rh.superiortemporal_16 | lh.middletemporal_16 | rh.precentral_18 | rh.precentral_2 |
| 620 | lh.parahippocampal_2 | lh.precentral_24 | rh.inferiorparietal_7 | rh.posteriorcingulate_4 | lh.lateraloccipital_16 | rh.superiorfrontal_35 | lh.lingual_7 |
| 621 | rh.postcentral_3 | lh.superiorparietal_9 | rh.paracentral_9 | lh.precentral_7 | lh.superiorparietal_14 | rh.precentral_35 | lh.lateraloccipital_3 |
| 622 | rh.fusiform_3 | lh.superiorfrontal_32 | lh.superiorparietal_21 | lh.rostralmiddlefrontal_24 | rh.lingual_3 | rh.posteriorcingulate_5 | lh.middletemporal_1 |
| 623 | lh.rostralmiddlefrontal_3 | lh.rostralmiddlefrontal_24 | lh.rostralmiddlefrontal_24 | rh.rostralmiddlefrontal_7 | rh.precentral_18 | lh.inferiortemporal_7 | rh.precentral_28 |
| 624 | lh.fusiform_2 | lh.superiorfrontal_12 | lh.superiorparietal_12 | rh.superiorfrontal_29 | lh.fusiform_5 | lh.superiorfrontal_20 | lh.inferiortemporal_3 |
| 625 | rh.rostralmiddlefrontal_13 | rh.cuneus_7 | lh.middletemporal_10 | rh.precuneus_21 | lh.postcentral_2 | rh.posteriorcingulate_7 | lh.supramarginal_20 |
| 626 | rh.postcentral_16 | rh.lingual_14 | lh.fusiform_2 | lh.precentral_24 | rh.transversetemporal_2 | rh.inferiorparietal_10 | rh.precuneus_21 |
| 627 | rh.pericalcarine_7 | rh.superiorfrontal_20 | rh.precentral_6 | lh.superiorparietal_21 | rh.superiorfrontal_21 | rh.superiorfrontal_42 | rh.lingual_5 |
| 628 | lh.superiorfrontal_19 | lh.pericalcarine_7 | lh.precuneus_1 | rh.inferiorparietal_26 | lh.superiorparietal_7 | lh.medialorbitofrontal_8 | rh.transversetemporal_3 |
| 629 | rh.lingual_4 | lh.caudalmiddlefrontal_12 | rh.rostralmiddlefrontal_16 | rh.paracentral_1 | lh.fusiform_12 | rh.postcentral_9 | rh.lateraloccipital_19 |
| 630 | lh.superiorfrontal_14 | lh.precentral_7 | rh.pericalcarine_8 | lh.superiorparietal_14 | lh.parahippocampal_2 | rh.superiortemporal_1 | lh.precentral_27 |
| 631 | lh.lingual_10 | lh.middletemporal_3 | lh.precentral_17 | lh.precuneus_19 | lh.fusiform_15 | rh.supramarginal_9 | rh.inferiortemporal_3 |
| 632 | rh.cuneus_8 | lh.lateraloccipital_14 | lh.postcentral_12 | rh.bankssts_5 | lh.pericalcarine_5 | lh.inferiortemporal_16 | rh.middletemporal_19 |
| 633 | rh.lateraloccipital_8 | rh.superiorfrontal_7 | rh.parstriangularis_3 | lh.insula_12 | rh.lateraloccipital_19 | lh.superiorfrontal_25 | rh.parahippocampal_3 |
| 634 | rh.lateraloccipital_19 | lh.superiorparietal_28 | rh.lingual_2 | lh.superiorparietal_12 | lh.superiorfrontal_34 | rh.lateraloccipital_11 | rh.rostralmiddlefrontal_1 |
| 635 | rh.insula_2 | rh.lateraloccipital_13 | lh.rostralmiddlefrontal_7 | rh.supramarginal_20 | rh.inferiortemporal_12 | lh.insula_16 | lh.precentral_34 |
| 636 | lh.lateralorbitofrontal_8 | rh.parsopercularis_4 | lh.superiorfrontal_39 | lh.bankssts_5 | lh.superiorparietal_5 | rh.lingual_10 | lh.fusiform_11 |
| 637 | lh.precentral_10 | rh.bankssts_4 | rh.paracentral_1 | lh.superiorparietal_29 | rh.supramarginal_18 | lh.superiorfrontal_31 | rh.precuneus_2 |
| 638 | lh.postcentral_2 | lh.supramarginal_11 | rh.precuneus_15 | rh.rostralanteriorcingulate_3 | lh.precentral_2 | lh.supramarginal_20 | rh.paracentral_1 |
| 639 | lh.pericalcarine_4 | lh.fusiform_4 | lh.superiorfrontal_12 | rh.bankssts_6 | lh.insula_3 | lh.rostralmiddlefrontal_17 | rh.precentral_17 |
| 640 | rh.superiorfrontal_5 | lh.supramarginal_20 | rh.precuneus_21 | lh.fusiform_2 | lh.postcentral_26 | lh.superiorfrontal_39 | rh.superiorparietal_21 |
| 641 | rh.inferiortemporal_7 | lh.paracentral_8 | rh.pericalcarine_6 | rh.precentral_1 | lh.inferiortemporal_1 | rh.postcentral_19 | lh.rostralmiddlefrontal_18 |
| 642 | lh.superiorparietal_8 | rh.superiorparietal_17 | lh.middletemporal_13 | lh.parstriangularis_6 | lh.inferiortemporal_4 | lh.bankssts_2 | rh.superiorfrontal_20 |

| | | | | | | | |
|---|---|---|---|---|---|---|---|
| 643 | lh.superiorparietal_23 | rh.precentral_6 | lh.lingual_12 | rh.precentral_10 | lh.rostralmiddlefrontal_18 | lh.posteriorcingulate_3 | rh.superiortemporal_16 |
| 644 | rh.superiorfrontal_38 | lh.fusiform_3 | lh.superiorparietal_14 | lh.cuneus_7 | rh.inferiortemporal_10 | rh.fusiform_10 | lh.lateraloccipital_16 |
| 645 | rh.postcentral_1 | rh.precentral_2 | rh.bankssts_6 | rh.precentral_6 | rh.pericalcarine_2 | rh.pericalcarine_4 | rh.parsorbitalis_3 |
| 646 | rh.inferiortemporal_10 | lh.fusiform_2 | lh.parahippocampal_2 | rh.insula_9 | lh.superiorfrontal_9 | lh.supramarginal_5 | lh.superiorfrontal_12 |
| 647 | rh.superiortemporal_25 | rh.rostralmiddlefrontal_15 | rh.precentral_2 | rh.parsopercularis_1 | rh.parstriangularis_4 | rh.fusiform_1 | rh.inferiortemporal_10 |
| 648 | lh.rostralmiddlefrontal_18 | lh.fusiform_8 | lh.precentral_7 | lh.supramarginal_12 | lh.superiorfrontal_18 | lh.insula_6 | rh.superiorfrontal_36 |
| 649 | lh.lateraloccipital_4 | rh.lateraloccipital_12 | lh.lingual_3 | lh.middletemporal_3 | rh.fusiform_8 | lh.superiortemporal_2 | lh.lingual_15 |
| 650 | lh.lateraloccipital_11 | lh.supramarginal_8 | lh.inferiorparietal_14 | lh.rostralmiddlefrontal_17 | lh.rostralmiddlefrontal_8 | rh.lateraloccipital_18 | lh.postcentral_28 |
| 651 | lh.transversetemporal_1 | rh.fusiform_6 | rh.parsopercularis_4 | lh.rostralmiddlefrontal_6 | lh.precentral_22 | lh.inferiorparietal_19 | rh.rostralmiddlefrontal_5 |
| 652 | rh.lingual_12 | lh.superiorparietal_14 | lh.insula_6 | lh.superiorfrontal_32 | lh.cuneus_6 | rh.lingual_12 | rh.precuneus_12 |
| 653 | lh.superiorfrontal_36 | rh.pericalcarine_8 | rh.supramarginal_2 | lh.supramarginal_11 | rh.precuneus_17 | rh.superiorfrontal_7 | lh.inferiortemporal_10 |
| 654 | lh.pericalcarine_2 | rh.postcentral_5 | rh.superiorparietal_17 | lh.supramarginal_8 | lh.superiorfrontal_16 | rh.precentral_3 | lh.parahippocampal_2 |
| 655 | rh.precentral_18 | lh.superiortemporal_20 | rh.precuneus_17 | lh.insula_7 | rh.superiorparietal_6 | lh.supramarginal_17 | lh.postcentral_15 |
| 656 | rh.superiorparietal_23 | rh.superiorfrontal_9 | lh.superiortemporal_20 | rh.superiorparietal_7 | lh.pericalcarine_4 | rh.paracentral_1 | lh.lateraloccipital_22 |
| 657 | rh.lateraloccipital_4 | lh.lingual_12 | lh.caudalmiddlefrontal_12 | rh.rostralmiddlefrontal_16 | lh.rostralmiddlefrontal_12 | rh.cuneus_1 | lh.lingual_17 |
| 658 | rh.inferiortemporal_12 | lh.lateraloccipital_15 | lh.parsopercularis_10 | lh.caudalmiddlefrontal_12 | lh.insula_6 | rh.precentral_26 | rh.middletemporal_4 |
| 659 | lh.lateralorbitofrontal_5 | lh.inferiortemporal_10 | rh.superiorparietal_7 | lh.fusiform_3 | lh.lateraloccipital_8 | lh.bankssts_3 | lh.precuneus_18 |
| 660 | rh.superiorfrontal_21 | rh.parsopercularis_8 | rh.bankssts_5 | rh.precentral_17 | rh.lateraloccipital_4 | lh.precentral_32 | rh.supramarginal_2 |
| 661 | rh.precentral_31 | rh.caudalmiddlefrontal_2 | rh.parsopercularis_1 | lh.superiorfrontal_39 | rh.superiorfrontal_1 | lh.supramarginal_11 | rh.fusiform_13 |
| 662 | rh.superiorparietal_7 | lh.postcentral_3 | Left-Accumbens-area | lh.inferiortemporal_10 | rh.postcentral_1 | rh.superiorparietal_19 | rh.lateralorbitofrontal_3 |
| 663 | rh.lateraloccipital_18 | lh.middletemporal_7 | lh.superiorfrontal_32 | lh.lateraloccipital_12 | rh.lateraloccipital_8 | lh.precentral_5 | rh.postcentral_19 |
| 664 | lh.superiorparietal_28 | rh.superiorfrontal_3 | lh.superiorparietal_5 | rh.superiorparietal_27 | rh.superiorfrontal_8 | lh.superiorparietal_26 | lh.superiortemporal_20 |
| 665 | lh.lateraloccipital_8 | rh.postcentral_25 | rh.postcentral_5 | rh.superiorparietal_20 | lh.lateraloccipital_11 | rh.entorhinal_1 | rh.fusiform_9 |
| 666 | lh.fusiform_1 | lh.rostralanteriorcingulate_3 | rh.superiortemporal_16 | lh.rostralanteriorcingulate_2 | rh.fusiform_3 | lh.superiorparietal_8 | rh.superiortemporal_11 |
| 667 | lh.superiortemporal_8 | rh.supramarginal_2 | lh.superiorparietal_28 | rh.superiorparietal_17 | rh.medialorbitofrontal_10 | lh.lateraloccipital_2 | rh.fusiform_2 |
| 668 | lh.lingual_5 | lh.postcentral_25 | lh.precuneus_19 | lh.lateraloccipital_10 | rh.superiorparietal_23 | lh.superiorfrontal_3 | lh.lingual_2 |
| 669 | rh.postcentral_18 | rh.superiorparietal_7 | rh.precentral_31 | lh.fusiform_8 | rh.supramarginal_15 | lh.superiortemporal_20 | lh.lateraloccipital_9 |
| 670 | lh.medialorbitofrontal_4 | rh.postcentral_20 | lh.inferiortemporal_4 | rh.parsopercularis_4 | lh.lateraloccipital_9 | rh.superiortemporal_20 | rh.superiortemporal_10 |
| 671 | lh.superiorfrontal_18 | rh.supramarginal_16 | rh.superiorparietal_20 | rh.insula_15 | lh.fusiform_2 | lh.inferiortemporal_6 | lh.inferiortemporal_6 |
| 672 | lh.superiorfrontal_15 | rh.lateraloccipital_21 | lh.transversetemporal_3 | lh.middletemporal_7 | rh.postcentral_18 | rh.entorhinal_2 | lh.lateraloccipital_7 |
| 673 | lh.precentral_22 | rh.cuneus_3 | lh.fusiform_8 | rh.lateralorbitofrontal_6 | lh.lateraloccipital_4 | lh.postcentral_9 | lh.fusiform_15 |
| 674 | lh.parahippocampal_4 | rh.postcentral_7 | lh.superiorparietal_8 | lh.precentral_34 | rh.postcentral_16 | rh.temporalpole_1 | rh.lateraloccipital_22 |
| 675 | lh.superiorfrontal_34 | lh.lateralorbitofrontal_10 | rh.parsopercularis_8 | lh.parahippocampal_2 | lh.postcentral_23 | rh.postcentral_3 | rh.temporalpole_1 |
| 676 | lh.lingual_8 | lh.parahippocampal_2 | lh.superiorparietal_7 | rh.superiortemporal_12 | lh.pericalcarine_3 | lh.insula_9 | rh.fusiform_4 |
| 677 | lh.rostralmiddlefrontal_16 | lh.rostralmiddlefrontal_7 | rh.cuneus_4 | lh.postcentral_11 | rh.lingual_2 | rh.lateraloccipital_16 | rh.lateraloccipital_15 |
| 678 | lh.superiorparietal_14 | lh.superiortemporal_25 | rh.rostralmiddlefrontal_7 | rh.superiorfrontal_3 | lh.rostralmiddlefrontal_16 | rh.fusiform_15 | rh.supramarginal_18 |
| 679 | lh.lateraloccipital_9 | rh.precuneus_22 | lh.middletemporal_3 | rh.bankssts_4 | rh.inferiortemporal_7 | rh.superiorparietal_1 | lh.superiorparietal_7 |
| 680 | rh.middletemporal_8 | rh.parahippocampal_3 | rh.rostralmiddlefrontal_11 | lh.precentral_1 | lh.medialorbitofrontal_5 | rh.caudalmiddlefrontal_3 | lh.fusiform_13 |
| 681 | rh.superiortemporal_24 | rh.pericalcarine_6 | rh.rostralanteriorcingulate_2 | lh.lateraloccipital_1 | rh.lingual_4 | rh.posteriorcingulate_8 | rh.fusiform_14 |
| 682 | lh.insula_9 | rh.cuneus_4 | lh.supramarginal_11 | rh.lingual_8 | rh.superiortemporal_25 | rh.postcentral_12 | lh.lateraloccipital_8 |
| 683 | rh.postcentral_12 | lh.postcentral_2 | rh.fusiform_6 | lh.lingual_13 | lh.insula_9 | rh.superiorfrontal_9 | lh.transversetemporal_2 |
| 684 | rh.precuneus_17 | rh.superiorparietal_6 | rh.superiorparietal_27 | lh.postcentral_25 | rh.rostralmiddlefrontal_11 | lh.pericalcarine_5 | rh.inferiortemporal_6 |
| 685 | rh.fusiform_5 | lh.lingual_3 | Right-Accumbens-area | lh.postcentral_2 | lh.lingual_14 | Left-Amygdala | rh.fusiform_15 |
| 686 | lh.lateralorbitofrontal_1 | lh.inferiorparietal_14 | rh.supramarginal_16 | rh.caudalmiddlefrontal_2 | rh.rostralmiddlefrontal_6 | lh.postcentral_10 | lh.superiortemporal_9 |
| 687 | lh.rostralmiddlefrontal_12 | rh.middletemporal_13 | lh.postcentral_26 | rh.rostralmiddlefrontal_15 | rh.rostralmiddlefrontal_1 | lh.insula_10 | rh.precentral_1 |
| 688 | rh.medialorbitofrontal_10 | rh.superiorparietal_29 | rh.inferiortemporal_6 | rh.supramarginal_2 | lh.fusiform_9 | lh.precentral_10 | rh.bankssts_4 |

| | | | | | | | |
|---|---|---|---|---|---|---|---|
| 689 | lh.superiorfrontal_4 | rh.precuneus_12 | rh.lateraloccipital_13 | lh.lingual_12 | lh.superiorparietal_23 | lh.precentral_22 | lh.insula_9 |
| 690 | lh.postcentral_17 | lh.superiorparietal_8 | rh.superiorfrontal_7 | lh.supramarginal_20 | lh.transversetemporal_1 | lh.precuneus_14 | rh.lingual_15 |
| 691 | lh.lateraloccipital_19 | lh.superiorparietal_26 | rh.rostralanteriorcingulate_1 | lh.inferiorparietal_14 | lh.paracentral_2 | rh.precentral_17 | lh.superiortemporal_14 |
| 692 | rh.postcentral_4 | lh.parsopercularis_10 | rh.superiorparietal_28 | lh.rostralmiddlefrontal_7 | lh.superiorfrontal_5 | rh.superiorfrontal_26 | rh.lateraloccipital_4 |
| 693 | lh.superiortemporal_26 | lh.parsopercularis_5 | rh.superiortemporal_12 | rh.superiorparietal_6 | rh.lateraloccipital_18 | rh.supramarginal_4 | lh.medialorbitofrontal_7 |
| 694 | rh.lingual_10 | rh.precentral_1 | rh.caudalmiddlefrontal_2 | lh.postcentral_3 | rh.superiorfrontal_15 | lh.lateraloccipital_16 | rh.superiorfrontal_7 |
| 695 | lh.lingual_1 | lh.superiorparietal_29 | lh.parsopercularis_5 | lh.supramarginal_14 | rh.pericalcarine_7 | rh.precentral_34 | rh.parsorbitalis_1 |
| 696 | lh.inferiortemporal_8 | rh.precentral_4 | rh.rostralmiddlefrontal_15 | rh.paracentral_9 | rh.middletemporal_8 | lh.supramarginal_7 | lh.middletemporal_16 |
| 697 | rh.parstriangularis_4 | rh.superiorparietal_20 | lh.precentral_9 | lh.superiorparietal_8 | lh.pericalcarine_2 | lh.precentral_31 | rh.superiorfrontal_12 |
| 698 | rh.superiorfrontal_1 | rh.paracentral_1 | lh.lingual_13 | rh.superiortemporal_19 | rh.superiortemporal_24 | lh.lateraloccipital_17 | lh.rostralmiddlefrontal_16 |
| 699 | rh.supramarginal_15 | rh.insula_3 | lh.lateraloccipital_15 | lh.rostralmiddlefrontal_16 | lh.lingual_10 | lh.bankssts_4 | rh.parahippocampal_1 |
| 700 | rh.superiorparietal_1 | lh.rostralmiddlefrontal_16 | lh.rostralanteriorcingulate_4 | rh.superiorparietal_29 | lh.postcentral_15 | lh.cuneus_4 | lh.postcentral_24 |
| 701 | rh.lateraloccipital_9 | rh.inferiorparietal_5 | rh.precentral_17 | rh.postcentral_5 | rh.lateralorbitofrontal_14 | lh.postcentral_24 | rh.rostralmiddlefrontal_25 |
| 702 | rh.insula_12 | rh.superiorfrontal_29 | rh.precentral_1 | lh.rostralanteriorcingulate_3 | lh.lingual_9 | rh.lateraloccipital_2 | lh.fusiform_17 |
| 703 | lh.postcentral_29 | rh.rostralanteriorcingulate_4 | lh.precentral_34 | lh.paracentral_2 | lh.lateraloccipital_19 | lh.lateraloccipital_3 | rh.superiorparietal_4 |
| 704 | lh.superiorparietal_9 | rh.parsopercularis_3 | lh.postcentral_3 | rh.superiorparietal_19 | rh.precentral_2 | lh.superiorfrontal_40 | rh.superiorparietal_3 |
| 705 | lh.inferiortemporal_9 | lh.insula_10 | rh.bankssts_4 | lh.rostralmiddlefrontal_12 | rh.postcentral_19 | rh.pericalcarine_7 | lh.rostralmiddlefrontal_25 |
| 706 | lh.postcentral_23 | lh.rostralmiddlefrontal_12 | lh.inferiortemporal_10 | lh.superiortemporal_20 | rh.pericalcarine_5 | rh.postcentral_18 | lh.postcentral_26 |
| 707 | lh.cuneus_2 | rh.insula_2 | rh.postcentral_20 | lh.lateraloccipital_13 | lh.superiortemporal_8 | lh.postcentral_16 | lh.fusiform_6 |
| 708 | rh.postcentral_10 | lh.lingual_13 | lh.supramarginal_8 | rh.parahippocampal_3 | lh.inferiortemporal_9 | rh.inferiortemporal_3 | lh.fusiform_16 |
| 709 | rh.pericalcarine_4 | lh.superiorparietal_7 | lh.rostralmiddlefrontal_12 | rh.parsopercularis_8 | lh.postcentral_24 | rh.superiortemporal_21 | rh.parahippocampal_2 |
| 710 | lh.precentral_2 | lh.postcentral_26 | lh.precentral_1 | lh.precentral_9 | lh.insula_11 | rh.superiortemporal_18 | rh.superiortemporal_12 |
| 711 | lh.superiorparietal_3 | lh.cuneus_7 | lh.paracentral_2 | lh.superiortemporal_25 | lh.lingual_8 | lh.superiorfrontal_18 | lh.transversetemporal_3 |
| 712 | lh.cuneus_5 | rh.precentral_17 | rh.superiorparietal_19 | lh.lateralorbitofrontal_10 | lh.postcentral_29 | rh.superiortemporal_14 | rh.lateraloccipital_16 |
| 713 | rh.lingual_9 | lh.rostralmiddlefrontal_18 | lh.superiorparietal_29 | rh.precuneus_22 | lh.precentral_27 | lh.precentral_29 | rh.paracentral_9 |
| 714 | lh.postcentral_24 | rh.superiortemporal_25 | lh.postcentral_25 | rh.middletemporal_13 | rh.superiorfrontal_9 | rh.inferiortemporal_1 | rh.transversetemporal_2 |
| 715 | rh.postcentral_19 | rh.parstriangularis_6 | lh.cuneus_7 | rh.supramarginal_16 | rh.lingual_12 | lh.precentral_11 | lh.precentral_9 |
| 716 | lh.rostralmiddlefrontal_14 | lh.superiorfrontal_39 | rh.lateraloccipital_12 | lh.parsopercularis_10 | rh.lingual_10 | rh.supramarginal_16 | rh.postcentral_18 |
| 717 | lh.postcentral_15 | lh.paracentral_2 | rh.precuneus_22 | rh.precentral_28 | lh.parahippocampal_4 | lh.precentral_14 | rh.lateralorbitofrontal_1 |
| 718 | rh.cuneus_6 | lh.supramarginal_9 | lh.rostralmiddlefrontal_16 | lh.precentral_27 | rh.rostralmiddlefrontal_25 | lh.precentral_25 | lh.lateralorbitofrontal_3 |
| 719 | rh.rostralmiddlefrontal_25 | lh.precentral_34 | lh.middletemporal_7 | lh.lingual_16 | rh.superiorfrontal_32 | rh.paracentral_9 | rh.superiortemporal_1 |
| 720 | lh.insula_3 | rh.paracentral_9 | lh.lateraloccipital_14 | rh.insula_2 | lh.lingual_1 | rh.precentral_31 | lh.supramarginal_14 |
| 721 | rh.lateraloccipital_16 | lh.insula_1 | lh.fusiform_4 | rh.precentral_4 | rh.postcentral_12 | lh.insula_13 | rh.fusiform_12 |
| 722 | lh.cuneus_4 | lh.inferiortemporal_1 | rh.superiorfrontal_29 | rh.precuneus_12 | rh.superiorparietal_20 | rh.postcentral_24 | rh.superiortemporal_6 |
| 723 | lh.fusiform_4 | rh.superiorparietal_19 | rh.superiortemporal_11 | rh.postcentral_7 | lh.superiortemporal_26 | lh.postcentral_19 | lh.middletemporal_14 |
| 724 | rh.superiorfrontal_8 | rh.precuneus_17 | lh.fusiform_3 | rh.precuneus_17 | lh.precuneus_14 | Brain-Stem | lh.inferiortemporal_1 |
| 725 | rh.precentral_2 | rh.rostralmiddlefrontal_3 | rh.superiortemporal_25 | lh.rostralmiddlefrontal_18 | lh.fusiform_1 | rh.superiorfrontal_16 | lh.superiortemporal_7 |
| 726 | lh.rostralmiddlefrontal_8 | rh.superiorparietal_27 | lh.postcentral_29 | rh.superiortemporal_25 | rh.lateraloccipital_16 | lh.middletemporal_10 | lh.fusiform_10 |
| 727 | lh.superiorfrontal_9 | rh.superiorparietal_23 | lh.rostralanteriorcingulate_1 | lh.superiorparietal_7 | rh.lateraloccipital_9 | rh.postcentral_5 | rh.superiorparietal_23 |
| 728 | lh.paracentral_2 | lh.lateraloccipital_10 | rh.lateraloccipital_21 | lh.postcentral_26 | lh.lateralorbitofrontal_13 | lh.temporalpole_3 | lh.precentral_18 |
| 729 | lh.lateraloccipital_2 | lh.transversetemporal_3 | lh.supramarginal_9 | lh.insula_17 | rh.cuneus_1 | rh.precentral_10 | lh.parahippocampal_1 |
| 730 | lh.superiortemporal_11 | lh.precentral_1 | rh.superiorparietal_6 | rh.rostralmiddlefrontal_11 | rh.postcentral_10 | lh.lateraloccipital_8 | rh.postcentral_1 |
| 731 | lh.medialorbitofrontal_5 | rh.supramarginal_18 | lh.superiorfrontal_16 | rh.postcentral_19 | lh.lateraloccipital_2 | lh.lingual_14 | lh.middletemporal_4 |
| 732 | lh.superiorfrontal_8 | rh.transversetemporal_2 | rh.precuneus_12 | rh.insula_3 | rh.lingual_1 | rh.pericalcarine_1 | lh.superiorfrontal_40 |
| 733 | lh.superiortemporal_4 | lh.supramarginal_14 | lh.precentral_31 | rh.inferiorparietal_5 | rh.fusiform_5 | rh.precentral_4 | lh.inferiortemporal_9 |
| 734 | rh.cuneus_5 | lh.lateraloccipital_12 | rh.parstriangularis_6 | lh.parsopercularis_5 | rh.superiorparietal_1 | rh.cuneus_6 | rh.superiorfrontal_39 |

| | | | | | | | |
|---|---|---|---|---|---|---|---|
| 735 | lh.precentral_27 | lh.precentral_27 | lh.lateraloccipital_10 | rh.insula_12 | rh.cuneus_8 | lh.lateralorbitofrontal_12 | lh.lateraloccipital_2 |
| 736 | lh.lateraloccipital_20 | lh.lateraloccipital_1 | lh.postcentral_11 | lh.postcentral_29 | rh.pericalcarine_4 | lh.supramarginal_8 | rh.postcentral_25 |
| 737 | rh.lateralorbitofrontal_12 | lh.middletemporal_16 | lh.superiortemporal_25 | rh.superiorparietal_26 | lh.superiorfrontal_30 | lh.pericalcarine_6 | lh.lateraloccipital_20 |
| 738 | lh.lingual_13 | rh.insula_12 | rh.middletemporal_13 | rh.parsopercularis_3 | lh.lateraloccipital_20 | rh.superiortemporal_17 | lh.pericalcarine_4 |
| 739 | lh.bankssts_1 | lh.postcentral_29 | rh.superiorfrontal_23 | rh.superiorparietal_23 | lh.superiorparietal_9 | lh.precentral_9 | rh.lateralorbitofrontal_2 |
| 740 | lh.insula_6 | Left-Accumbens-area | lh.precuneus_14 | rh.lingual_13 | lh.superiortemporal_4 | lh.superiorfrontal_15 | rh.fusiform_10 |
| 741 | rh.lateraloccipital_14 | rh.postcentral_19 | lh.precentral_27 | lh.insula_3 | lh.cuneus_2 | rh.bankssts_5 | lh.lateraloccipital_19 |
| 742 | rh.middletemporal_10 | rh.fusiform_4 | lh.lingual_16 | lh.superiorfrontal_16 | lh.inferiortemporal_8 | lh.superiorfrontal_6 | rh.pericalcarine_2 |
| 743 | lh.superiorfrontal_11 | rh.superiortemporal_12 | lh.rostralmiddlefrontal_14 | lh.inferiortemporal_1 | lh.paracentral_8 | lh.precentral_20 | lh.superiortemporal_25 |
| 744 | lh.superiorfrontal_5 | rh.inferiortemporal_10 | rh.superiorparietal_29 | rh.superiorfrontal_23 | rh.precentral_28 | lh.postcentral_6 | rh.lateralorbitofrontal_5 |
| 745 | rh.superiorfrontal_15 | rh.superiortemporal_6 | rh.rostralmiddlefrontal_3 | rh.middletemporal_10 | lh.bankssts_1 | rh.lateraloccipital_9 | lh.fusiform_8 |
| 746 | rh.lateraloccipital_10 | lh.precentral_9 | rh.precentral_4 | rh.supramarginal_18 | lh.superiorfrontal_33 | lh.pericalcarine_4 | lh.temporalpole_3 |
| 747 | rh.superiorparietal_19 | Right-Accumbens-area | lh.insula_9 | rh.parstriangularis_6 | rh.paracentral_9 | rh.superiorfrontal_36 | lh.lateraloccipital_11 |
| 748 | rh.pericalcarine_3 | rh.precentral_28 | lh.supramarginal_14 | lh.inferiortemporal_9 | rh.lingual_9 | lh.superiorfrontal_33 | lh.fusiform_5 |
| 749 | rh.lateralorbitofrontal_14 | rh.lingual_17 | rh.lingual_17 | rh.middletemporal_8 | lh.superiorparietal_3 | rh.superiortemporal_10 | lh.fusiform_7 |
| 750 | rh.paracentral_9 | lh.parsopercularis_2 | rh.lingual_8 | rh.inferiortemporal_10 | lh.superiortemporal_11 | rh.postcentral_11 | rh.middletemporal_8 |
| 751 | rh.superiorparietal_20 | rh.middletemporal_8 | rh.transversetemporal_2 | lh.insula_10 | rh.lateraloccipital_14 | lh.parstriangularis_7 | lh.postcentral_29 |
| 752 | rh.parahippocampal_6 | rh.rostralmiddlefrontal_11 | rh.superiorparietal_23 | lh.supramarginal_9 | lh.postcentral_17 | rh.parstriangularis_1 | lh.superiortemporal_4 |
| 753 | lh.precuneus_14 | lh.insula_3 | rh.inferiorparietal_5 | rh.transversetemporal_2 | lh.superiorfrontal_39 | rh.supramarginal_7 | rh.lateraloccipital_8 |
| 754 | rh.rostralmiddlefrontal_11 | lh.lateraloccipital_13 | lh.parsopercularis_2 | rh.rostralmiddlefrontal_3 | rh.cuneus_6 | lh.lingual_11 | rh.superiortemporal_24 |
| 755 | rh.rostralmiddlefrontal_6 | lh.postcentral_11 | lh.lateralorbitofrontal_2 | lh.precuneus_14 | rh.postcentral_4 | lh.postcentral_3 | lh.bankssts_1 |
| 756 | rh.cuneus_3 | lh.inferiortemporal_9 | rh.lateralorbitofrontal_14 | lh.superiorparietal_3 | rh.superiorfrontal_7 | rh.supramarginal_1 | rh.postcentral_20 |
| 757 | rh.lateralorbitofrontal_5 | rh.superiortemporal_11 | rh.parsopercularis_3 | rh.rostralanteriorcingulate_4 | lh.precentral_30 | lh.lingual_10 | rh.precuneus_22 |
| 758 | lh.superiorparietal_17 | lh.precentral_14 | rh.lateralorbitofrontal_12 | lh.insula_1 | rh.lateralorbitofrontal_5 | lh.lingual_1 | lh.fusiform_3 |
| 759 | lh.superiortemporal_3 | lh.superiorparietal_3 | rh.superiorfrontal_3 | lh.middletemporal_16 | rh.superiorfrontal_36 | lh.precentral_1 | rh.lingual_17 |
| 760 | rh.rostralmiddlefrontal_9 | rh.middletemporal_10 | rh.lateralorbitofrontal_5 | rh.supramarginal_15 | lh.cuneus_5 | rh.superiorparietal_27 | lh.postcentral_3 |
| 761 | rh.superiorfrontal_32 | rh.supramarginal_15 | lh.superiortemporal_8 | rh.superiortemporal_2 | rh.lateraloccipital_10 | lh.postcentral_5 | rh.lateraloccipital_23 |
| 762 | rh.superiorfrontal_9 | rh.postcentral_18 | lh.superiorparietal_3 | rh.superiorfrontal_39 | lh.precentral_34 | rh.parstriangularis_5 | lh.inferiortemporal_4 |
| 763 | rh.fusiform_7 | rh.inferiortemporal_12 | rh.postcentral_18 | rh.superiortemporal_7 | rh.middletemporal_10 | rh.bankssts_6 | rh.medialorbitofrontal_6 |
| 764 | lh.lateraloccipital_15 | rh.rostralanteriorcingulate_1 | lh.postcentral_23 | Left-Accumbens-area | rh.lateralorbitofrontal_3 | rh.superiortemporal_5 | rh.lateralorbitofrontal_17 |
| 765 | rh.superiorfrontal_7 | lh.superiorfrontal_16 | lh.lateraloccipital_12 | lh.transversetemporal_3 | rh.superiorfrontal_4 | rh.supramarginal_14 | rh.lateraloccipital_14 |
| 766 | lh.superiorfrontal_16 | lh.lingual_16 | lh.insula_13 | lh.parsopercularis_2 | lh.insula_17 | rh.superiortemporal_23 | lh.superiortemporal_2 |
| 767 | lh.postcentral_22 | lh.rostralanteriorcingulate_4 | rh.postcentral_19 | rh.fusiform_4 | rh.lateralorbitofrontal_12 | lh.superiortemporal_14 | rh.superiortemporal_18 |
| 768 | lh.lingual_11 | lh.insula_17 | rh.postcentral_7 | rh.superiortemporal_6 | lh.cuneus_4 | lh.lateraloccipital_4 | rh.lateraloccipital_13 |
| 769 | rh.precentral_28 | lh.precuneus_14 | lh.rostralmiddlefrontal_18 | lh.rostralmiddlefrontal_14 | rh.cuneus_5 | rh.superiortemporal_24 | lh.inferiortemporal_2 |
| 770 | rh.lateraloccipital_13 | rh.lingual_8 | lh.inferiortemporal_1 | lh.insula_11 | lh.parstriangularis_1 | rh.cuneus_8 | rh.superiortemporal_8 |
| 771 | lh.pericalcarine_1 | lh.rostralmiddlefrontal_14 | rh.postcentral_1 | rh.postcentral_18 | rh.superiorfrontal_23 | lh.supramarginal_1 | rh.lingual_10 |
| 772 | rh.transversetemporal_1 | rh.postcentral_1 | lh.lateralorbitofrontal_1 | rh.lateraloccipital_20 | rh.lateralorbitofrontal_1 | rh.parahippocampal_2 | lh.lateraloccipital_4 |
| 773 | lh.superiorfrontal_30 | lh.fusiform_9 | rh.middletemporal_10 | rh.inferiortemporal_12 | lh.lingual_5 | rh.precentral_21 | lh.entorhinal_3 |
| 774 | rh.lateralorbitofrontal_1 | rh.parstriangularis_4 | rh.parahippocampal_1 | rh.superiorfrontal_10 | rh.precentral_17 | lh.inferiortemporal_5 | lh.pericalcarine_6 |
| 775 | rh.postcentral_25 | lh.superiortemporal_8 | rh.rostralmiddlefrontal_6 | Right-Accumbens-area | rh.superiorparietal_19 | rh.precentral_5 | lh.lingual_1 |
| 776 | rh.lateralorbitofrontal_3 | rh.lingual_13 | rh.lingual_13 | lh.precentral_30 | rh.postcentral_25 | rh.middletemporal_15 | rh.rostralmiddlefrontal_12 |
| 777 | rh.cuneus_7 | rh.parstriangularis_7 | rh.superiorfrontal_39 | rh.parstriangularis_4 | rh.superiorfrontal_20 | rh.fusiform_3 | rh.postcentral_10 |
| 778 | lh.superiorfrontal_33 | rh.superiorfrontal_23 | lh.transversetemporal_1 | rh.rostralmiddlefrontal_6 | rh.lateraloccipital_13 | lh.postcentral_22 | rh.fusiform_3 |
| 779 | lh.postcentral_16 | lh.inferiortemporal_8 | rh.precentral_30 | lh.lateraloccipital_6 | lh.precentral_9 | lh.superiortemporal_8 | lh.precentral_31 |
| 780 | rh.superiorfrontal_36 | lh.insula_11 | lh.medialorbitofrontal_5 | rh.lingual_17 | lh.superiorfrontal_12 | lh.postcentral_26 | rh.lateraloccipital_9 |

| | | | | | | | |
|---|---|---|---|---|---|---|---|
| 781 | rh.pericalcarine_8 | rh.rostralmiddlefrontal_6 | rh.superiortemporal_24 | lh.superiortemporal_19 | rh.precentral_1 | rh.postcentral_15 | rh.fusiform_8 |
| 782 | rh.lateraloccipital_12 | rh.inferiortemporal_7 | lh.lateraloccipital_13 | rh.postcentral_1 | rh.rostralmiddlefrontal_9 | rh.lingual_13 | rh.lateraloccipital_18 |
| 783 | rh.fusiform_6 | lh.parahippocampal_4 | lh.lateralorbitofrontal_8 | lh.precentral_14 | lh.supramarginal_14 | rh.inferiortemporal_11 | lh.precentral_1 |
| 784 | lh.precentral_9 | lh.rostralanteriorcingulate_1 | rh.superiortemporal_6 | lh.precentral_31 | lh.fusiform_4 | rh.middletemporal_14 | rh.inferiortemporal_7 |
| 785 | lh.cuneus_3 | lh.precentral_30 | lh.lateraloccipital_1 | rh.fusiform_11 | lh.lateraloccipital_15 | rh.cuneus_5 | rh.superiortemporal_9 |
| 786 | rh.postcentral_17 | rh.rostralanteriorcingulate_2 | lh.fusiform_9 | rh.superiortemporal_11 | lh.superiorparietal_17 | lh.lateraloccipital_6 | lh.superiortemporal_26 |
| 787 | lh.precentral_34 | lh.postcentral_23 | rh.middletemporal_8 | lh.parahippocampal_1 | lh.precentral_18 | rh.fusiform_14 | rh.lateraloccipital_12 |
| 788 | lh.supramarginal_14 | rh.postcentral_12 | rh.lateralorbitofrontal_4 | lh.precentral_18 | lh.lingual_11 | lh.lateraloccipital_20 | rh.lateraloccipital_21 |
| 789 | lh.rostralmiddlefrontal_1 | rh.superiortemporal_24 | lh.parahippocampal_1 | rh.rostralanteriorcingulate_1 | rh.superiorfrontal_12 | rh.inferiortemporal_15 | rh.lateraloccipital_10 |
| 790 | lh.precentral_18 | rh.lateraloccipital_20 | rh.supramarginal_15 | lh.superiortemporal_8 | rh.lateraloccipital_12 | lh.inferiortemporal_11 | rh.superiortemporal_25 |
| 791 | lh.fusiform_14 | lh.transversetemporal_1 | lh.superiorfrontal_8 | lh.fusiform_9 | lh.postcentral_28 | rh.supramarginal_15 | lh.lingual_12 |
| 792 | rh.precentral_1 | lh.superiorfrontal_8 | rh.supramarginal_18 | lh.inferiortemporal_8 | lh.pericalcarine_1 | rh.inferiortemporal_7 | rh.postcentral_28 |
| 793 | rh.postcentral_22 | lh.rostralmiddlefrontal_3 | lh.superiorparietal_26 | rh.superiorfrontal_4 | rh.paracentral_1 | rh.supramarginal_18 | lh.middletemporal_2 |
| 794 | lh.postcentral_1 | rh.postcentral_16 | rh.postcentral_12 | lh.rostralanteriorcingulate_4 | rh.parahippocampal_6 | lh.middletemporal_4 | rh.superiorparietal_6 |
| 795 | lh.insula_11 | lh.precentral_18 | lh.lateralorbitofrontal_7 | lh.postcentral_20 | rh.superiorfrontal_10 | lh.supramarginal_13 | lh.fusiform_2 |
| 796 | rh.precentral_17 | rh.lateralorbitofrontal_12 | lh.parahippocampal_4 | lh.parahippocampal_4 | rh.fusiform_7 | lh.transversetemporal_2 | rh.pericalcarine_4 |
| 797 | rh.lateraloccipital_21 | lh.lateralorbitofrontal_8 | rh.superiortemporal_19 | lh.postcentral_23 | rh.transversetemporal_1 | lh.cuneus_1 | lh.superiortemporal_11 |
| 798 | lh.precentral_30 | rh.superiorfrontal_10 | rh.superiorfrontal_10 | lh.superiorfrontal_8 | rh.parahippocampal_1 | rh.postcentral_13 | rh.superiortemporal_20 |
| 799 | lh.paracentral_8 | rh.superiorfrontal_4 | rh.inferiortemporal_10 | rh.lateralorbitofrontal_12 | rh.lateraloccipital_21 | lh.postcentral_12 | lh.lingual_6 |
| 800 | lh.superiorparietal_29 | lh.medialorbitofrontal_4 | rh.superiorparietal_4 | rh.inferiortemporal_7 | lh.postcentral_16 | lh.fusiform_15 | rh.middletemporal_10 |
| 801 | rh.superiorfrontal_20 | rh.rostralmiddlefrontal_25 | lh.inferiortemporal_8 | rh.postcentral_12 | lh.superiorfrontal_40 | rh.inferiortemporal_2 | lh.superiorparietal_5 |
| 802 | rh.superiorfrontal_12 | rh.rostralmiddlefrontal_9 | lh.inferiortemporal_9 | rh.rostralmiddlefrontal_9 | rh.cuneus_3 | rh.fusiform_17 | lh.lingual_11 |
| 803 | lh.parstriangularis_1 | rh.superiorfrontal_39 | rh.precentral_28 | rh.transversetemporal_1 | rh.postcentral_20 | lh.superiorfrontal_5 | lh.parstriangularis_5 |
| 804 | lh.superiorfrontal_12 | lh.insula_6 | lh.middletemporal_16 | rh.cuneus_2 | rh.pericalcarine_3 | lh.bankssts_6 | rh.rostralmiddlefrontal_9 |
| 805 | rh.postcentral_15 | rh.transversetemporal_1 | rh.parstriangularis_4 | rh.superiortemporal_15 | rh.superiorparietal_4 | lh.parstriangularis_5 | rh.postcentral_16 |
| 806 | lh.lateraloccipital_14 | lh.lateraloccipital_6 | rh.superiortemporal_9 | rh.superiortemporal_24 | lh.precentral_1 | rh.postcentral_6 | rh.lingual_4 |
| 807 | lh.precentral_1 | rh.medialorbitofrontal_10 | rh.medialorbitofrontal_10 | rh.parstriangularis_7 | rh.pericalcarine_8 | rh.precentral_6 | rh.postcentral_12 |
| 808 | lh.pericalcarine_7 | rh.fusiform_7 | rh.fusiform_4 | lh.insula_13 | rh.rostralmiddlefrontal_1 | lh.fusiform_5 | lh.pericalcarine_5 |
| 809 | rh.postcentral_23 | lh.superiortemporal_19 | lh.precentral_18 | lh.superiortemporal_17 | rh.middletemporal_2 | lh.precentral_27 | rh.frontalpole_2 |
| 810 | lh.superiorfrontal_39 | rh.lateralorbitofrontal_14 | rh.superiorfrontal_4 | rh.fusiform_7 | lh.lingual_13 | lh.postcentral_1 | rh.superiorfrontal_29 |
| 811 | rh.rostralmiddlefrontal_17 | rh.fusiform_11 | rh.superiortemporal_7 | lh.insula_6 | rh.superiortemporal_12 | lh.entorhinal_2 | lh.lateraloccipital_15 |
| 812 | rh.superiorfrontal_4 | rh.postcentral_10 | lh.precentral_14 | rh.parahippocampal_1 | lh.superiortemporal_3 | rh.bankssts_2 | lh.transversetemporal_1 |
| 813 | rh.postcentral_20 | lh.postcentral_20 | rh.lateralorbitofrontal_3 | rh.superiortemporal_9 | rh.superiortemporal_18 | lh.middletemporal_2 | rh.postcentral_7 |
| 814 | rh.parahippocampal_1 | lh.superiortemporal_4 | rh.rostralmiddlefrontal_9 | rh.rostralmiddlefrontal_25 | lh.cuneus_3 | rh.superiorfrontal_32 | rh.middletemporal_11 |
| 815 | lh.superiorfrontal_40 | rh.fusiform_3 | rh.cuneus_2 | lh.transversetemporal_1 | rh.postcentral_15 | lh.superiorfrontal_12 | lh.pericalcarine_2 |
| 816 | rh.parstriangularis_5 | lh.precentral_31 | rh.postcentral_17 | rh.rostralanteriorcingulate_2 | rh.postcentral_17 | lh.fusiform_6 | rh.superiorparietal_1 |
| 817 | rh.cuneus_4 | rh.postcentral_17 | rh.postcentral_16 | rh.lateralorbitofrontal_14 | lh.pericalcarine_7 | lh.lingual_8 | rh.fusiform_17 |
| 818 | rh.lingual_16 | lh.insula_9 | lh.superiortemporal_4 | rh.superiorparietal_4 | lh.lateraloccipital_13 | rh.superiortemporal_12 | rh.rostralmiddlefrontal_26 |
| 819 | rh.lingual_11 | rh.parahippocampal_1 | rh.transversetemporal_1 | rh.postcentral_15 | rh.cuneus_7 | rh.postcentral_26 | rh.lateralorbitofrontal_12 |
| 820 | rh.inferiortemporal_2 | rh.cuneus_2 | lh.postcentral_24 | rh.postcentral_17 | rh.lateralorbitofrontal_15 | rh.fusiform_7 | lh.superiorparietal_23 |
| 821 | lh.postcentral_28 | lh.postcentral_16 | rh.parstriangularis_7 | lh.rostralanteriorcingulate_1 | lh.lateraloccipital_10 | lh.middletemporal_1 | rh.superiorparietal_19 |
| 822 | lh.lateraloccipital_1 | rh.postcentral_15 | rh.lateralorbitofrontal_13 | rh.postcentral_16 | lh.lateraloccipital_14 | lh.pericalcarine_3 | lh.cuneus_1 |
| 823 | lh.lateraloccipital_13 | lh.bankssts_1 | rh.inferiortemporal_7 | lh.rostralmiddlefrontal_3 | lh.fusiform_14 | lh.fusiform_16 | rh.parstriangularis_2 |
| 824 | rh.paracentral_1 | lh.medialorbitofrontal_5 | rh.lateraloccipital_20 | lh.lateralorbitofrontal_2 | rh.rostralmiddlefrontal_17 | rh.lingual_9 | rh.medialorbitofrontal_8 |
| 825 | rh.superiortemporal_18 | lh.insula_13 | rh.inferiortemporal_12 | lh.lateralorbitofrontal_8 | rh.inferiortemporal_16 | rh.lateraloccipital_14 | lh.lingual_8 |
| 826 | lh.lateralorbitofrontal_12 | lh.postcentral_24 | lh.rostralmiddlefrontal_3 | lh.medialorbitofrontal_4 | lh.postcentral_22 | rh.superiortemporal_6 | lh.lateraloccipital_10 |

| | | | | | | | |
|---|---|---|---|---|---|---|---|
| 827 | lh.lateraloccipital_10 | lh.lateralorbitofrontal_1 | lh.medialorbitofrontal_4 | lh.superiortemporal_5 | lh.medialorbitofrontal_7 | lh.entorhinal_1 | lh.lateraloccipital_13 |
| 828 | lh.superiortemporal_13 | lh.superiortemporal_26 | lh.lateralorbitofrontal_13 | lh.postcentral_16 | lh.superiorparietal_29 | rh.middletemporal_8 | lh.superiorparietal_17 |
| 829 | lh.superiortemporal_5 | lh.superiorfrontal_11 | lh.medialorbitofrontal_7 | lh.superiortemporal_4 | rh.middletemporal_19 | lh.superiorparietal_23 | rh.lateralorbitofrontal_16 |
| 830 | lh.insula_17 | rh.superiorparietal_4 | rh.superiortemporal_2 | rh.medialorbitofrontal_10 | lh.lateraloccipital_1 | rh.fusiform_4 | lh.cuneus_2 |
| 831 | rh.superiorparietal_4 | rh.lateralorbitofrontal_5 | lh.postcentral_20 | rh.superiortemporal_13 | rh.superiorfrontal_29 | lh.inferiortemporal_1 | lh.superiorparietal_9 |
| 832 | rh.superiorparietal_27 | rh.superiortemporal_2 | lh.postcentral_4 | rh.postcentral_10 | rh.lateraloccipital_20 | rh.precentral_1 | rh.fusiform_1 |
| 833 | rh.middletemporal_6 | rh.postcentral_4 | rh.postcentral_15 | lh.parstriangularis_1 | rh.superiortemporal_23 | rh.precentral_2 | lh.pericalcarine_1 |
| 834 | lh.cuneus_7 | rh.superiortemporal_15 | lh.lateraloccipital_6 | rh.lateraloccipital_1 | rh.middletemporal_6 | rh.transversetemporal_2 | lh.superiortemporal_8 |
| 835 | rh.pericalcarine_6 | lh.parstriangularis_1 | lh.superiortemporal_19 | lh.bankssts_1 | rh.inferiortemporal_2 | rh.middletemporal_10 | rh.lingual_9 |
| 836 | rh.lateraloccipital_20 | lh.superiortemporal_5 | lh.superiortemporal_5 | rh.postcentral_28 | rh.fusiform_6 | lh.cuneus_6 | lh.inferiortemporal_11 |
| 837 | lh.parstriangularis_7 | rh.superiortemporal_7 | lh.superiortemporal_26 | rh.lateralorbitofrontal_5 | rh.parstriangularis_5 | rh.rostralmiddlefrontal_8 | rh.superiortemporal_23 |
| 838 | lh.lingual_4 | rh.superiortemporal_19 | lh.parstriangularis_1 | rh.inferiortemporal_2 | lh.postcentral_1 | rh.parahippocampal_6 | lh.fusiform_12 |
| 839 | rh.inferiortemporal_11 | lh.parahippocampal_1 | rh.fusiform_11 | lh.medialorbitofrontal_5 | rh.parstriangularis_2 | lh.superiortemporal_10 | rh.cuneus_5 |
| 840 | rh.superiortemporal_12 | lh.postcentral_17 | rh.parahippocampal_6 | lh.superiorfrontal_11 | lh.lingual_4 | rh.superiortemporal_9 | rh.pericalcarine_1 |
| 841 | rh.superiortemporal_23 | lh.superiortemporal_3 | lh.postcentral_16 | rh.lateralorbitofrontal_4 | rh.lingual_16 | rh.superiortemporal_15 | lh.lingual_14 |
| 842 | rh.middletemporal_14 | rh.superiortemporal_9 | rh.superiortemporal_15 | lh.insula_9 | lh.rostralmiddlefrontal_11 | rh.lateralorbitofrontal_2 | lh.parahippocampal_4 |
| 843 | lh.lingual_16 | rh.inferiortemporal_2 | lh.superiorfrontal_11 | lh.postcentral_4 | lh.inferiortemporal_6 | lh.supramarginal_14 | rh.cuneus_6 |
| 844 | rh.superiorfrontal_29 | lh.lateralorbitofrontal_2 | rh.rostralmiddlefrontal_25 | lh.postcentral_24 | lh.parstriangularis_5 | rh.postcentral_7 | rh.fusiform_5 |
| 845 | lh.transversetemporal_4 | rh.lateralorbitofrontal_3 | rh.fusiform_7 | lh.superiortemporal_16 | rh.inferiortemporal_5 | lh.parahippocampal_6 | lh.rostralmiddlefrontal_11 |
| 846 | rh.parstriangularis_2 | lh.lateralorbitofrontal_5 | lh.bankssts_1 | rh.fusiform_3 | lh.cuneus_7 | rh.postcentral_16 | rh.pericalcarine_7 |
| 847 | rh.rostralmiddlefrontal_1 | lh.superiortemporal_11 | rh.postcentral_22 | lh.superiortemporal_3 | rh.cuneus_4 | rh.pericalcarine_5 | rh.transversetemporal_1 |
| 848 | rh.superiorfrontal_23 | rh.lingual_11 | lh.lateralorbitofrontal_5 | lh.superiortemporal_26 | rh.superiorparietal_27 | rh.superiortemporal_16 | lh.superiorparietal_3 |
| 849 | rh.cuneus_2 | rh.parstriangularis_5 | rh.superiortemporal_13 | rh.lingual_11 | rh.inferiortemporal_11 | lh.lateraloccipital_9 | lh.postcentral_16 |
| 850 | lh.lateralorbitofrontal_13 | rh.lateralorbitofrontal_4 | rh.fusiform_3 | lh.lateralorbitofrontal_1 | lh.inferiortemporal_15 | rh.bankssts_3 | rh.lateraloccipital_20 |
| 851 | lh.rostralmiddlefrontal_11 | lh.rostralmiddlefrontal_8 | rh.postcentral_28 | rh.lateralorbitofrontal_3 | rh.parsorbitalis_1 | lh.superiortemporal_18 | lh.fusiform_9 |
| 852 | lh.lateraloccipital_12 | rh.lateraloccipital_1 | lh.superiortemporal_11 | rh.postcentral_4 | lh.lateralorbitofrontal_7 | lh.pericalcarine_7 | lh.postcentral_11 |
| 853 | lh.parahippocampal_6 | rh.lateralorbitofrontal_1 | lh.superiortemporal_17 | rh.parstriangularis_5 | lh.parstriangularis_7 | lh.fusiform_9 | lh.lateralorbitofrontal_7 |
| 854 | rh.lingual_13 | rh.inferiortemporal_11 | lh.lateralorbitofrontal_6 | rh.rostralmiddlefrontal_1 | lh.postcentral_11 | rh.superiorfrontal_29 | rh.entorhinal_1 |
| 855 | rh.superiorfrontal_10 | rh.rostralmiddlefrontal_1 | lh.postcentral_17 | rh.inferiortemporal_11 | lh.lateraloccipital_12 | lh.superiortemporal_4 | lh.inferiortemporal_8 |
| 856 | lh.parstriangularis_5 | lh.postcentral_4 | rh.medialorbitofrontal_8 | rh.inferiortemporal_1 | rh.postcentral_23 | lh.fusiform_2 | lh.pericalcarine_3 |
| 857 | lh.inferiortemporal_5 | lh.fusiform_14 | rh.superiortemporal_23 | lh.transversetemporal_4 | lh.fusiform_13 | rh.middletemporal_5 | lh.parahippocampal_3 |
| 858 | lh.insula_13 | rh.middletemporal_6 | lh.parstriangularis_5 | rh.parahippocampal_5 | rh.postcentral_22 | lh.pericalcarine_1 | lh.pericalcarine_7 |
| 859 | lh.medialorbitofrontal_7 | rh.parahippocampal_6 | rh.parahippocampal_5 | rh.parstriangularis_2 | rh.middletemporal_14 | lh.fusiform_14 | lh.lateralorbitofrontal_11 |
| 860 | rh.lateralorbitofrontal_4 | lh.transversetemporal_4 | lh.superiortemporal_13 | lh.postcentral_17 | lh.insula_13 | lh.superiortemporal_3 | rh.pericalcarine_8 |
| 861 | rh.lingual_14 | rh.parstriangularis_2 | rh.lateralorbitofrontal_1 | rh.middletemporal_6 | rh.lateralorbitofrontal_4 | rh.inferiortemporal_10 | rh.postcentral_15 |
| 862 | lh.lateralorbitofrontal_7 | lh.superiortemporal_13 | rh.superiorfrontal_40 | lh.inferiortemporal_5 | lh.parahippocampal_6 | lh.insula_1 | lh.lingual_10 |
| 863 | lh.postcentral_20 | rh.postcentral_28 | lh.superiortemporal_3 | lh.lateralorbitofrontal_7 | rh.superiorfrontal_39 | rh.fusiform_12 | lh.cuneus_6 |
| 864 | lh.middletemporal_5 | lh.inferiortemporal_5 | lh.fusiform_14 | rh.superiorfrontal_40 | rh.fusiform_10 | rh.transversetemporal_3 | rh.rostralmiddlefrontal_17 |
| 865 | lh.middletemporal_9 | lh.lateralorbitofrontal_7 | lh.rostralmiddlefrontal_1 | rh.parstriangularis_1 | lh.superiortemporal_13 | lh.transversetemporal_3 | rh.middletemporal_6 |
| 866 | lh.postcentral_11 | rh.postcentral_22 | rh.rostralmiddlefrontal_1 | rh.lateralorbitofrontal_13 | rh.superiortemporal_9 | lh.postcentral_15 | lh.cuneus_4 |
| 867 | lh.lateraloccipital_6 | lh.postcentral_22 | rh.postcentral_4 | lh.superiortemporal_21 | rh.middletemporal_11 | lh.inferiortemporal_3 | lh.lateraloccipital_1 |
| 868 | lh.lingual_3 | lh.parstriangularis_7 | lh.inferiortemporal_5 | lh.parstriangularis_5 | lh.precentral_31 | rh.medialorbitofrontal_1 | rh.lingual_12 |
| 869 | rh.fusiform_11 | lh.parstriangularis_5 | rh.postcentral_10 | lh.superiortemporal_13 | lh.lateraloccipital_6 | rh.fusiform_13 | lh.lingual_4 |
| 870 | rh.middletemporal_19 | lh.lateralorbitofrontal_13 | rh.lingual_11 | lh.postcentral_6 | lh.lateralorbitofrontal_12 | rh.lateraloccipital_12 | lh.lateralorbitofrontal_15 |
| 871 | rh.superiortemporal_15 | rh.rostralmiddlefrontal_17 | rh.lateraloccipital_1 | lh.inferiortemporal_15 | rh.fusiform_1 | rh.inferiortemporal_12 | rh.lingual_3 |
| 872 | lh.lateralorbitofrontal_2 | lh.rostralmiddlefrontal_1 | rh.inferiortemporal_1 | lh.fusiform_14 | rh.medialorbitofrontal_4 | lh.middletemporal_3 | lh.fusiform_1 |

| | | | | | | | |
|---|---|---|---|---|---|---|---|
| 873 | lh.superiortemporal_19 | lh.medialorbitofrontal_7 | rh.inferiortemporal_2 | lh.lateralorbitofrontal_6 | rh.pericalcarine_6 | rh.superiortemporal_25 | rh.fusiform_7 |
| 874 | rh.middletemporal_2 | rh.inferiortemporal_1 | lh.lateralorbitofrontal_9 | lh.superiortemporal_11 | rh.lingual_14 | rh.lateraloccipital_8 | rh.middletemporal_9 |
| 875 | lh.postcentral_27 | rh.superiortemporal_23 | rh.middletemporal_6 | rh.lateralorbitofrontal_1 | lh.superiortemporal_5 | lh.pericalcarine_2 | rh.lingual_7 |
| 876 | lh.inferiortemporal_6 | rh.lateralorbitofrontal_13 | lh.rostralmiddlefrontal_8 | lh.medialorbitofrontal_7 | rh.superiorfrontal_40 | lh.superiortemporal_9 | lh.lateraloccipital_14 |
| 877 | rh.middletemporal_9 | lh.postcentral_1 | rh.parstriangularis_1 | rh.inferiortemporal_16 | lh.inferiortemporal_5 | rh.postcentral_1 | rh.inferiortemporal_2 |
| 878 | lh.postcentral_4 | rh.parstriangularis_1 | lh.medialorbitofrontal_10 | lh.lateralorbitofrontal_13 | lh.postcentral_20 | lh.lateraloccipital_1 | lh.entorhinal_2 |
| 879 | rh.fusiform_1 | rh.postcentral_23 | lh.postcentral_6 | lh.parstriangularis_7 | rh.middletemporal_9 | lh.middletemporal_7 | lh.cuneus_5 |
| 880 | rh.parsorbitalis_1 | lh.rostralmiddlefrontal_11 | lh.inferiortemporal_15 | lh.rostralmiddlefrontal_8 | rh.superiortemporal_15 | lh.inferiortemporal_12 | rh.parstriangularis_5 |
| 881 | rh.middletemporal_11 | lh.superiortemporal_17 | lh.parahippocampal_3 | rh.parahippocampal_6 | lh.lingual_3 | lh.postcentral_29 | rh.parstriangularis_1 |
| 882 | rh.lateralorbitofrontal_13 | lh.lateralorbitofrontal_12 | rh.lateralorbitofrontal_11 | lh.lateralorbitofrontal_5 | rh.middletemporal_18 | rh.middletemporal_4 | lh.postcentral_17 |
| 883 | rh.superiortemporal_9 | rh.inferiortemporal_16 | lh.postcentral_22 | rh.postcentral_22 | lh.lateralorbitofrontal_2 | lh.cuneus_3 | rh.rostralmiddlefrontal_8 |
| 884 | rh.inferiortemporal_16 | lh.postcentral_28 | lh.transversetemporal_4 | lh.lateralorbitofrontal_9 | lh.lateralorbitofrontal_3 | lh.cuneus_2 | lh.cuneus_3 |
| 885 | lh.precentral_31 | rh.superiorfrontal_40 | rh.inferiortemporal_16 | rh.rostralmiddlefrontal_17 | lh.lingual_16 | lh.fusiform_8 | rh.lateralorbitofrontal_9 |
| 886 | rh.lateralorbitofrontal_15 | lh.inferiortemporal_15 | lh.parstriangularis_7 | rh.superiortemporal_23 | rh.lingual_13 | lh.transversetemporal_4 | rh.postcentral_4 |
| 887 | rh.inferiortemporal_5 | lh.middletemporal_9 | lh.lateralorbitofrontal_3 | rh.medialorbitofrontal_8 | lh.transversetemporal_4 | lh.precentral_18 | lh.medialorbitofrontal_2 |
| 888 | lh.inferiortemporal_13 | rh.middletemporal_19 | lh.postcentral_1 | rh.middletemporal_19 | lh.postcentral_27 | lh.inferiortemporal_2 | lh.lingual_9 |
| 889 | rh.superiorfrontal_39 | rh.superiortemporal_13 | lh.lateralorbitofrontal_4 | rh.middletemporal_9 | rh.medialorbitofrontal_8 | lh.superiortemporal_5 | rh.parahippocampal_6 |
| 890 | rh.lateraloccipital_1 | rh.middletemporal_9 | lh.postcentral_27 | rh.lateralorbitofrontal_11 | rh.lateralorbitofrontal_13 | rh.postcentral_20 | lh.lateraloccipital_6 |
| 891 | rh.lingual_8 | rh.middletemporal_2 | rh.parstriangularis_5 | lh.postcentral_22 | rh.lingual_11 | lh.lateraloccipital_12 | rh.lateralorbitofrontal_13 |
| 892 | rh.fusiform_10 | lh.postcentral_27 | lh.inferiortemporal_6 | lh.superiortemporal_2 | lh.postcentral_4 | rh.bankssts_4 | rh.lateralorbitofrontal_4 |
| 893 | rh.inferiortemporal_1 | rh.middletemporal_14 | rh.middletemporal_14 | rh.middletemporal_2 | rh.rostralmiddlefrontal_12 | lh.parahippocampal_3 | lh.fusiform_4 |
| 894 | rh.parstriangularis_1 | lh.inferiortemporal_6 | rh.superiortemporal_18 | lh.parahippocampal_3 | rh.superiortemporal_20 | lh.cuneus_5 | rh.postcentral_17 |
| 895 | lh.fusiform_13 | rh.medialorbitofrontal_8 | lh.inferiortemporal_14 | lh.middletemporal_9 | lh.middletemporal_12 | lh.lateraloccipital_19 | rh.lingual_16 |
| 896 | rh.medialorbitofrontal_8 | lh.superiortemporal_16 | lh.temporalpole_2 | lh.rostralmiddlefrontal_1 | rh.postcentral_28 | lh.middletemporal_13 | rh.superiortemporal_15 |
| 897 | lh.inferiortemporal_15 | rh.superiortemporal_18 | rh.middletemporal_19 | lh.rostralmiddlefrontal_11 | rh.parstriangularis_1 | lh.lateraloccipital_13 | rh.inferiortemporal_11 |
| 898 | rh.superiortemporal_20 | rh.fusiform_1 | rh.rostralmiddlefrontal_17 | rh.superiortemporal_22 | rh.cuneus_2 | rh.postcentral_4 | rh.lingual_2 |
| 899 | lh.lateralorbitofrontal_3 | lh.superiortemporal_23 | lh.rostralmiddlefrontal_11 | lh.temporalpole_2 | rh.inferiortemporal_1 | lh.lateraloccipital_10 | lh.cuneus_7 |
| 900 | rh.rostralmiddlefrontal_12 | lh.superiortemporal_21 | lh.parahippocampal_6 | lh.superiortemporal_23 | lh.parahippocampal_1 | lh.postcentral_11 | lh.lateraloccipital_12 |
| 901 | rh.postcentral_28 | rh.fusiform_10 | lh.superiortemporal_16 | lh.postcentral_27 | rh.inferiortemporal_3 | rh.inferiortemporal_8 | lh.entorhinal_1 |
| 902 | lh.lateralorbitofrontal_11 | lh.parahippocampal_6 | rh.parstriangularis_2 | lh.postcentral_1 | lh.middletemporal_9 | lh.superiortemporal_22 | lh.lateralorbitofrontal_2 |
| 903 | lh.parstriangularis_3 | lh.superiortemporal_2 | lh.superiortemporal_21 | lh.lateralorbitofrontal_12 | lh.middletemporal_5 | lh.superiortemporal_21 | lh.superiorparietal_29 |
| 904 | lh.superiortemporal_23 | lh.fusiform_13 | rh.inferiortemporal_11 | rh.postcentral_23 | rh.fusiform_11 | lh.superiorparietal_17 | rh.inferiortemporal_1 |
| 905 | rh.superiortemporal_5 | lh.lateralorbitofrontal_6 | lh.middletemporal_9 | lh.inferiortemporal_6 | lh.rostralmiddlefrontal_25 | rh.lateraloccipital_20 | rh.cuneus_3 |
| 906 | rh.superiorfrontal_40 | lh.postcentral_6 | rh.middletemporal_9 | lh.postcentral_28 | rh.inferiortemporal_9 | rh.cuneus_7 | lh.fusiform_14 |
| 907 | lh.inferiortemporal_14 | rh.superiortemporal_20 | rh.medialorbitofrontal_4 | lh.inferiortemporal_14 | rh.parsorbitalis_3 | lh.middletemporal_16 | lh.insula_13 |
| 908 | lh.middletemporal_8 | lh.inferiortemporal_14 | rh.superiortemporal_5 | rh.fusiform_10 | lh.lateralorbitofrontal_11 | lh.inferiortemporal_10 | rh.superiorparietal_27 |
| 909 | rh.lateralorbitofrontal_16 | rh.parahippocampal_5 | lh.entorhinal_2 | rh.superiortemporal_20 | lh.middletemporal_2 | lh.cuneus_7 | rh.pericalcarine_5 |
| 910 | lh.middletemporal_2 | lh.parstriangularis_3 | lh.superiortemporal_23 | lh.fusiform_13 | rh.lateralorbitofrontal_17 | rh.lingual_14 | rh.lingual_14 |
| 911 | rh.medialorbitofrontal_3 | lh.temporalpole_2 | lh.postcentral_28 | rh.fusiform_1 | lh.superiortemporal_2 | lh.lateraloccipital_15 | lh.temporalpole_1 |
| 912 | rh.middletemporal_18 | lh.parstriangularis_4 | rh.middletemporal_2 | lh.parstriangularis_4 | rh.superiortemporal_8 | rh.middletemporal_3 | rh.cuneus_4 |
| 913 | lh.parahippocampal_1 | lh.middletemporal_2 | rh.entorhinal_2 | lh.middletemporal_12 | lh.middletemporal_4 | rh.superiortemporal_2 | rh.cuneus_8 |
| 914 | rh.lateralorbitofrontal_17 | lh.middletemporal_4 | rh.superiortemporal_22 | rh.middletemporal_14 | rh.temporalpole_2 | rh.cuneus_4 | lh.postcentral_27 |
| 915 | rh.rostralmiddlefrontal_26 | lh.lateralorbitofrontal_3 | rh.fusiform_10 | lh.medialorbitofrontal_1 | rh.lateralorbitofrontal_2 | lh.frontalpole_1 | rh.cuneus_7 |
| 916 | rh.superiortemporal_8 | lh.middletemporal_12 | rh.postcentral_23 | lh.middletemporal_4 | rh.medialorbitofrontal_11 | rh.transversetemporal_1 | lh.lingual_3 |
| 917 | lh.superiortemporal_2 | lh.inferiortemporal_13 | lh.entorhinal_1 | lh.middletemporal_2 | lh.entorhinal_3 | lh.lingual_4 | rh.pericalcarine_3 |
| 918 | lh.parstriangularis_4 | rh.medialorbitofrontal_3 | rh.medialorbitofrontal_3 | lh.parstriangularis_3 | rh.lateralorbitofrontal_16 | lh.fusiform_12 | lh.middletemporal_15 |

| | | | | | | | |
|---|---|---|---|---|---|---|---|
| 919 | lh.rostralmiddlefrontal_25 | lh.middletemporal_5 | rh.lateralorbitofrontal_15 | lh.medialorbitofrontal_10 | lh.temporalpole_2 | lh.bankssts_1 | lh.superiortemporal_3 |
| 920 | lh.entorhinal_3 | lh.middletemporal_8 | lh.fusiform_13 | rh.medialorbitofrontal_3 | rh.rostralmiddlefrontal_26 | rh.lingual_8 | rh.inferiortemporal_8 |
| 921 | rh.lateralorbitofrontal_2 | rh.superiortemporal_5 | rh.lateralorbitofrontal_16 | lh.temporalpole_3 | lh.fusiform_18 | rh.postcentral_25 | lh.frontalpole_1 |
| 922 | rh.medialorbitofrontal_4 | lh.medialorbitofrontal_10 | rh.superiortemporal_20 | lh.parahippocampal_6 | rh.lateraloccipital_1 | lh.fusiform_1 | lh.postcentral_20 |
| 923 | lh.inferiortemporal_11 | rh.inferiortemporal_5 | lh.lateralorbitofrontal_12 | lh.lateralorbitofrontal_3 | rh.rostralmiddlefrontal_5 | lh.postcentral_17 | lh.postcentral_4 |
| 924 | lh.middletemporal_4 | lh.lateralorbitofrontal_9 | rh.lateralorbitofrontal_8 | lh.lateralorbitofrontal_4 | lh.superiortemporal_19 | rh.fusiform_5 | rh.medialorbitofrontal_1 |
| 925 | lh.superiortemporal_22 | lh.superiortemporal_10 | lh.middletemporal_4 | lh.middletemporal_8 | lh.inferiortemporal_11 | rh.cuneus_3 | lh.parahippocampal_6 |
| 926 | rh.medialorbitofrontal_1 | rh.lateralorbitofrontal_16 | rh.fusiform_1 | lh.superiortemporal_10 | lh.rostralmiddlefrontal_2 | lh.fusiform_10 | lh.parstriangularis_7 |
| 927 | rh.inferiortemporal_3 | rh.parsorbitalis_1 | lh.medialorbitofrontal_1 | rh.superiortemporal_18 | lh.parstriangularis_3 | rh.inferiortemporal_6 | lh.parstriangularis_4 |
| 928 | lh.middletemporal_15 | rh.lateralorbitofrontal_15 | lh.middletemporal_2 | rh.superiortemporal_5 | rh.lingual_8 | lh.transversetemporal_1 | lh.postcentral_1 |
| 929 | rh.parsorbitalis_3 | rh.inferiortemporal_3 | rh.parahippocampal_4 | rh.entorhinal_2 | rh.superiortemporal_5 | lh.parahippocampal_4 | lh.medialorbitofrontal_6 |
| 930 | lh.middletemporal_12 | rh.rostralmiddlefrontal_26 | rh.parsorbitalis_1 | rh.inferiortemporal_8 | lh.inferiortemporal_13 | rh.middletemporal_11 | lh.inferiortemporal_5 |
| 931 | rh.inferiortemporal_9 | rh.middletemporal_11 | rh.temporalpole_2 | rh.lateralorbitofrontal_10 | lh.parstriangularis_4 | lh.postcentral_20 | rh.lingual_1 |
| 932 | lh.superiortemporal_10 | rh.rostralmiddlefrontal_12 | lh.superiortemporal_2 | rh.inferiortemporal_3 | lh.temporalpole_3 | lh.middletemporal_15 | rh.middletemporal_14 |
| 933 | lh.medialorbitofrontal_10 | rh.middletemporal_18 | lh.middletemporal_8 | lh.inferiortemporal_2 | lh.middletemporal_15 | lh.postcentral_27 | rh.fusiform_6 |
| 934 | rh.medialorbitofrontal_11 | rh.medialorbitofrontal_4 | lh.fusiform_18 | lh.inferiortemporal_13 | rh.medialorbitofrontal_1 | lh.middletemporal_14 | lh.postcentral_22 |
| 935 | rh.inferiortemporal_4 | rh.medialorbitofrontal_1 | lh.parstriangularis_3 | rh.parahippocampal_4 | rh.fusiform_16 | lh.inferiortemporal_8 | lh.lingual_13 |
| 936 | rh.middletemporal_3 | lh.temporalpole_3 | rh.rostralmiddlefrontal_26 | rh.lateralorbitofrontal_16 | lh.lateralorbitofrontal_15 | rh.frontalpole_2 | lh.parstriangularis_3 |
| 937 | lh.parsorbitalis_3 | lh.superiortemporal_22 | lh.temporalpole_3 | lh.medialorbitofrontal_2 | rh.lateralorbitofrontal_8 | rh.pericalcarine_3 | lh.lateralorbitofrontal_6 |
| 938 | lh.lateralorbitofrontal_15 | rh.inferiortemporal_9 | rh.lateralorbitofrontal_17 | rh.rostralmiddlefrontal_26 | lh.inferiortemporal_14 | rh.superiortemporal_22 | rh.middletemporal_5 |
| 939 | lh.medialorbitofrontal_8 | lh.middletemporal_15 | rh.rostralmiddlefrontal_8 | rh.medialorbitofrontal_6 | lh.middletemporal_8 | lh.inferiortemporal_9 | rh.superiortemporal_5 |
| 940 | rh.rostralmiddlefrontal_5 | lh.parsorbitalis_3 | rh.inferiortemporal_3 | rh.inferiortemporal_9 | lh.entorhinal_2 | lh.parahippocampal_2 | rh.superiortemporal_2 |
| 941 | rh.temporalpole_2 | lh.entorhinal_3 | lh.parstriangularis_4 | lh.parsorbitalis_3 | rh.medialorbitofrontal_3 | lh.lateraloccipital_14 | lh.lingual_5 |
| 942 | rh.superiortemporal_2 | lh.rostralmiddlefrontal_25 | lh.middletemporal_12 | rh.medialorbitofrontal_1 | rh.rostralmiddlefrontal_8 | lh.superiortemporal_25 | lh.parsorbitalis_3 |
| 943 | lh.fusiform_18 | rh.lateralorbitofrontal_11 | lh.middletemporal_15 | lh.fusiform_18 | lh.medialorbitofrontal_2 | rh.fusiform_8 | rh.cuneus_1 |
| 944 | lh.entorhinal_2 | rh.superiortemporal_22 | rh.lateralorbitofrontal_10 | rh.medialorbitofrontal_4 | lh.superiortemporal_22 | rh.fusiform_11 | rh.postcentral_23 |
| 945 | rh.rostralmiddlefrontal_8 | rh.inferiortemporal_4 | lh.medialorbitofrontal_6 | lh.superiortemporal_12 | lh.parsorbitalis_3 | lh.inferiortemporal_4 | rh.superiortemporal_17 |
| 946 | lh.medialorbitofrontal_6 | lh.fusiform_18 | rh.medialorbitofrontal_11 | rh.inferiortemporal_4 | lh.superiortemporal_23 | lh.superiorparietal_29 | rh.pericalcarine_6 |
| 947 | lh.temporalpole_2 | lh.medialorbitofrontal_2 | rh.rostralmiddlefrontal_12 | lh.superiortemporal_22 | rh.superiortemporal_10 | rh.lingual_16 | rh.lateraloccipital_1 |
| 948 | rh.superiortemporal_7 | rh.lateralorbitofrontal_17 | rh.inferiortemporal_5 | lh.middletemporal_15 | lh.medialorbitofrontal_10 | rh.superiortemporal_13 | lh.superiortemporal_13 |
| 949 | lh.medialorbitofrontal_2 | rh.lateralorbitofrontal_2 | lh.inferiortemporal_13 | rh.middletemporal_18 | lh.medialorbitofrontal_6 | lh.superiortemporal_13 | rh.lingual_13 |
| 950 | lh.rostralmiddlefrontal_2 | lh.parahippocampal_3 | rh.middletemporal_11 | rh.inferiortemporal_5 | lh.superiortemporal_10 | lh.superiortemporal_26 | lh.lateralorbitofrontal_12 |
| 951 | lh.temporalpole_3 | lh.lateralorbitofrontal_11 | rh.medialorbitofrontal_6 | rh.temporalpole_2 | rh.temporalpole_3 | rh.lateraloccipital_1 | lh.parsorbitalis_1 |
| 952 | lh.superiortemporal_21 | rh.temporalpole_2 | lh.middletemporal_5 | lh.entorhinal_3 | lh.superiortemporal_7 | lh.lingual_3 | rh.fusiform_11 |
| 953 | lh.lateralorbitofrontal_6 | rh.entorhinal_2 | rh.medialorbitofrontal_1 | lh.rostralmiddlefrontal_25 | rh.medialorbitofrontal_6 | lh.temporalpole_1 | lh.medialorbitofrontal_10 |
| 954 | lh.postcentral_6 | lh.inferiortemporal_11 | rh.inferiortemporal_9 | rh.rostralmiddlefrontal_12 | rh.middletemporal_3 | lh.middletemporal_9 | lh.superiortemporal_5 |
| 955 | lh.entorhinal_1 | rh.inferiortemporal_8 | lh.entorhinal_3 | lh.medialorbitofrontal_6 | rh.superiortemporal_2 | rh.superiortemporal_7 | rh.middletemporal_3 |
| 956 | rh.entorhinal_2 | lh.medialorbitofrontal_6 | rh.middletemporal_18 | lh.middletemporal_5 | lh.lateralorbitofrontal_6 | lh.fusiform_4 | rh.entorhinal_2 |
| 957 | rh.inferiortemporal_8 | lh.lateralorbitofrontal_4 | lh.superiortemporal_10 | rh.lateralorbitofrontal_15 | lh.entorhinal_1 | rh.frontalpole_1 | lh.postcentral_6 |
| 958 | rh.lateralorbitofrontal_8 | lh.entorhinal_2 | lh.rostralmiddlefrontal_2 | lh.entorhinal_2 | lh.medialorbitofrontal_8 | lh.medialorbitofrontal_10 | lh.superiortemporal_10 |
| 959 | rh.medialorbitofrontal_6 | rh.superiortemporal_8 | lh.rostralmiddlefrontal_25 | rh.lateralorbitofrontal_2 | rh.superiortemporal_1 | lh.superiortemporal_23 | lh.transversetemporal_4 |
| 960 | rh.middletemporal_17 | rh.medialorbitofrontal_6 | rh.lateralorbitofrontal_2 | rh.parsorbitalis_1 | lh.inferiortemporal_2 | lh.postcentral_4 | lh.superiortemporal_22 |
| 961 | rh.superiortemporal_13 | rh.parsorbitalis_3 | rh.inferiortemporal_4 | rh.lateralorbitofrontal_17 | rh.entorhinal_1 | rh.lingual_3 | lh.middletemporal_9 |
| 962 | lh.superiortemporal_16 | lh.medialorbitofrontal_1 | lh.inferiortemporal_2 | lh.rostralmiddlefrontal_2 | rh.superiortemporal_7 | rh.cuneus_2 | lh.inferiortemporal_14 |
| 963 | rh.parahippocampal_5 | lh.rostralmiddlefrontal_2 | lh.lateralorbitofrontal_11 | lh.entorhinal_1 | rh.inferiortemporal_8 | lh.middletemporal_5 | lh.middletemporal_5 |
| 964 | rh.superiortemporal_10 | rh.rostralmiddlefrontal_8 | rh.medialorbitofrontal_5 | rh.lateralorbitofrontal_9 | lh.postcentral_6 | lh.lingual_13 | lh.lingual_16 |

| | | | | | | | |
|---|---|---|---|---|---|---|---|
| 965 | lh.superiortemporal_7 | lh.inferiortemporal_2 | rh.inferiortemporal_8 | rh.middletemporal_11 | rh.inferiortemporal_4 | lh.lingual_9 | rh.medialorbitofrontal_3 |
| 966 | rh.entorhinal_1 | rh.medialorbitofrontal_11 | lh.parsorbitalis_3 | rh.lateralorbitofrontal_7 | rh.lateralorbitofrontal_9 | rh.postcentral_23 | rh.parahippocampal_5 |
| 967 | rh.lateralorbitofrontal_9 | rh.parahippocampal_4 | rh.lateralorbitofrontal_9 | rh.rostralmiddlefrontal_8 | lh.superiortemporal_21 | lh.lingual_16 | lh.middletemporal_8 |
| 968 | rh.parsorbitalis_2 | lh.entorhinal_1 | lh.superiortemporal_22 | lh.inferiortemporal_11 | rh.entorhinal_2 | rh.parahippocampal_4 | lh.frontalpole_2 |
| 969 | lh.lateralorbitofrontal_4 | lh.medialorbitofrontal_8 | rh.entorhinal_1 | rh.lateralorbitofrontal_8 | rh.parahippocampal_5 | lh.lateralorbitofrontal_4 | rh.lingual_8 |
| 970 | lh.superiortemporal_17 | rh.lateralorbitofrontal_8 | lh.inferiortemporal_11 | lh.superiortemporal_6 | rh.superiortemporal_13 | rh.middletemporal_6 | rh.postcentral_22 |
| 971 | rh.fusiform_16 | rh.lateralorbitofrontal_9 | rh.parsorbitalis_3 | lh.lateralorbitofrontal_11 | lh.parahippocampal_3 | rh.postcentral_17 | rh.parahippocampal_4 |
| 972 | rh.temporalpole_3 | lh.lateralorbitofrontal_15 | lh.medialorbitofrontal_2 | rh.parsorbitalis_3 | rh.middletemporal_17 | rh.lingual_1 | rh.superiortemporal_7 |
| 973 | lh.inferiortemporal_2 | rh.middletemporal_3 | rh.medialorbitofrontal_2 | rh.medialorbitofrontal_11 | lh.lateralorbitofrontal_4 | rh.pericalcarine_6 | rh.cuneus_2 |
| 974 | rh.parahippocampal_4 | rh.superiortemporal_10 | rh.superiortemporal_10 | rh.medialorbitofrontal_2 | rh.parahippocampal_4 | lh.frontalpole_2 | rh.superiortemporal_13 |
| 975 | rh.superiortemporal_1 | rh.lateralorbitofrontal_10 | rh.parsorbitalis_2 | rh.superiortemporal_10 | lh.lateralorbitofrontal_9 | lh.superiortemporal_19 | lh.superiortemporal_19 |
| 976 | lh.lateralorbitofrontal_9 | rh.parsorbitalis_2 | rh.rostralmiddlefrontal_5 | rh.superiortemporal_8 | lh.superiortemporal_14 | rh.lingual_2 | lh.medialorbitofrontal_8 |
| 977 | lh.rostralmiddlefrontal_20 | rh.rostralmiddlefrontal_5 | lh.medialorbitofrontal_9 | rh.parsorbitalis_4 | lh.rostralmiddlefrontal_20 | rh.middletemporal_1 | rh.lingual_11 |
| 978 | lh.middletemporal_11 | rh.entorhinal_1 | rh.lateralorbitofrontal_7 | rh.superiortemporal_17 | lh.superiortemporal_17 | rh.superiortemporal_4 | lh.lateralorbitofrontal_4 |
| 979 | lh.parsorbitalis_2 | lh.superiortemporal_12 | lh.superiortemporal_6 | rh.parsorbitalis_2 | rh.parsorbitalis_2 | rh.inferiortemporal_4 | lh.inferiortemporal_13 |
| 980 | rh.superiortemporal_19 | rh.middletemporal_17 | rh.fusiform_16 | lh.parsorbitalis_2 | rh.middletemporal_5 | rh.medialorbitofrontal_9 | lh.lateralorbitofrontal_9 |
| 981 | rh.superiortemporal_22 | lh.parsorbitalis_2 | lh.superiortemporal_12 | lh.medialorbitofrontal_9 | lh.parsorbitalis_2 | lh.middletemporal_8 | lh.superiortemporal_21 |
| 982 | rh.lateralorbitofrontal_11 | lh.rostralmiddlefrontal_20 | rh.superiortemporal_17 | lh.medialorbitofrontal_8 | lh.frontalpole_1 | lh.inferiortemporal_14 | lh.parsorbitalis_2 |
| 983 | Brain-Stem | rh.fusiform_16 | rh.superiortemporal_8 | rh.entorhinal_1 | lh.superiortemporal_16 | rh.fusiform_6 | lh.rostralmiddlefrontal_20 |
| 984 | lh.medialorbitofrontal_1 | rh.parsorbitalis_4 | rh.parsorbitalis_4 | lh.lateralorbitofrontal_14 | rh.temporalpole_1 | rh.parahippocampal_5 | rh.middletemporal_1 |
| 985 | lh.parahippocampal_3 | lh.medialorbitofrontal_9 | lh.medialorbitofrontal_8 | rh.medialorbitofrontal_5 | rh.superiortemporal_22 | rh.postcentral_22 | rh.frontalpole_1 |
| 986 | rh.middletemporal_5 | lh.superiortemporal_6 | rh.middletemporal_17 | lh.rostralmiddlefrontal_20 | lh.middletemporal_11 | lh.inferiortemporal_13 | rh.middletemporal_17 |
| 987 | rh.parsorbitalis_4 | rh.superiortemporal_1 | lh.rostralmiddlefrontal_20 | rh.middletemporal_17 | lh.parsorbitalis_1 | lh.lingual_5 | lh.superiortemporal_23 |
| 988 | lh.medialorbitofrontal_9 | lh.middletemporal_11 | rh.temporalpole_1 | lh.lateralorbitofrontal_15 | rh.lateralorbitofrontal_11 | lh.lateralorbitofrontal_9 | rh.medialorbitofrontal_9 |
| 989 | lh.parsorbitalis_1 | rh.superiortemporal_17 | rh.middletemporal_3 | lh.lateralorbitofrontal_16 | rh.superiortemporal_19 | rh.parsorbitalis_2 | rh.superiortemporal_22 |
| 990 | lh.frontalpole_1 | rh.medialorbitofrontal_5 | lh.parsorbitalis_4 | rh.fusiform_16 | lh.medialorbitofrontal_1 | lh.rostralmiddlefrontal_20 | rh.inferiortemporal_4 |
| 991 | rh.lateralorbitofrontal_10 | rh.medialorbitofrontal_2 | lh.lateralorbitofrontal_16 | rh.middletemporal_3 | lh.frontalpole_2 | rh.lingual_11 | lh.medialorbitofrontal_1 |
| 992 | rh.medialorbitofrontal_5 | rh.lateralorbitofrontal_7 | lh.lateralorbitofrontal_15 | rh.rostralmiddlefrontal_5 | rh.superiortemporal_17 | lh.superiortemporal_17 | lh.superiortemporal_17 |
| 993 | lh.medialorbitofrontal_3 | rh.temporalpole_3 | lh.middletemporal_11 | lh.parsorbitalis_4 | lh.temporalpole_1 | lh.superiortemporal_16 | rh.parsorbitalis_2 |
| 994 | lh.superiortemporal_6 | lh.lateralorbitofrontal_14 | lh.parsorbitalis_2 | rh.superiortemporal_1 | lh.medialorbitofrontal_9 | lh.lateralorbitofrontal_16 | rh.lateralorbitofrontal_11 |
| 995 | lh.superiortemporal_12 | lh.superiortemporal_7 | rh.frontalpole_2 | lh.middletemporal_11 | rh.parsorbitalis_4 | rh.lateralorbitofrontal_11 | rh.superiortemporal_4 |
| 996 | rh.middletemporal_7 | lh.parsorbitalis_4 | lh.lateralorbitofrontal_14 | rh.frontalpole_1 | lh.medialorbitofrontal_3 | rh.superiortemporal_19 | lh.medialorbitofrontal_9 |
| 997 | lh.frontalpole_2 | lh.lateralorbitofrontal_16 | rh.superiortemporal_1 | lh.superiortemporal_15 | rh.medialorbitofrontal_5 | rh.medialorbitofrontal_7 | rh.parsorbitalis_4 |
| 998 | lh.superiortemporal_14 | rh.frontalpole_1 | lh.frontalpole_2 | rh.temporalpole_1 | rh.frontalpole_1 | rh.medialorbitofrontal_2 | rh.superiortemporal_19 |
| 999 | rh.superiortemporal_17 | lh.medialorbitofrontal_3 | lh.temporalpole_1 | rh.frontalpole_2 | rh.medialorbitofrontal_9 | rh.parsorbitalis_4 | rh.medialorbitofrontal_2 |
| 1000 | rh.frontalpole_1 | lh.parsorbitalis_1 | rh.temporalpole_3 | lh.medialorbitofrontal_3 | rh.middletemporal_7 | lh.medialorbitofrontal_1 | lh.medialorbitofrontal_3 |
| 1001 | rh.medialorbitofrontal_9 | rh.middletemporal_5 | rh.middletemporal_5 | rh.temporalpole_3 | rh.lateralorbitofrontal_10 | rh.medialorbitofrontal_5 | lh.middletemporal_11 |
| 1002 | lh.parsorbitalis_4 | rh.temporalpole_1 | rh.frontalpole_1 | lh.parsorbitalis_1 | rh.frontalpole_2 | rh.lateralorbitofrontal_10 | rh.medialorbitofrontal_5 |
| 1003 | rh.medialorbitofrontal_2 | lh.frontalpole_2 | lh.superiortemporal_7 | lh.frontalpole_2 | rh.medialorbitofrontal_2 | lh.medialorbitofrontal_3 | rh.middletemporal_7 |
| 1004 | rh.temporalpole_1 | lh.frontalpole_1 | lh.medialorbitofrontal_3 | lh.temporalpole_1 | rh.middletemporal_1 | rh.middletemporal_17 | lh.lateralorbitofrontal_16 |
| 1005 | lh.temporalpole_1 | lh.temporalpole_1 | lh.frontalpole_1 | rh.middletemporal_5 | lh.superiortemporal_6 | rh.middletemporal_7 | lh.superiortemporal_16 |
| 1006 | rh.medialorbitofrontal_7 | rh.middletemporal_7 | rh.medialorbitofrontal_9 | lh.superiortemporal_7 | lh.superiortemporal_12 | lh.lateralorbitofrontal_14 | rh.medialorbitofrontal_7 |
| 1007 | lh.lateralorbitofrontal_16 | lh.superiortemporal_14 | lh.parsorbitalis_1 | lh.frontalpole_1 | lh.parsorbitalis_4 | lh.medialorbitofrontal_9 | lh.lateralorbitofrontal_14 |
| 1008 | rh.middletemporal_1 | rh.medialorbitofrontal_9 | lh.superiortemporal_15 | rh.medialorbitofrontal_9 | lh.lateralorbitofrontal_16 | lh.middletemporal_11 | rh.lateralorbitofrontal_10 |
| 1009 | lh.lateralorbitofrontal_14 | lh.superiortemporal_15 | lh.superiortemporal_14 | rh.medialorbitofrontal_7 | rh.medialorbitofrontal_7 | lh.superiortemporal_6 | lh.parsorbitalis_4 |
| 1010 | rh.lateralorbitofrontal_7 | rh.frontalpole_2 | rh.middletemporal_1 | lh.superiortemporal_14 | lh.lateralorbitofrontal_14 | lh.parsorbitalis_4 | lh.superiortemporal_6 |

| | | | | | | | |
|---|---|---|---|---|---|---|---|
| 1011 | rh.frontalpole_2 | rh.medialorbitofrontal_7 | rh.middletemporal_7 | rh.superiortemporal_4 | rh.lateralorbitofrontal_7 | rh.lateralorbitofrontal_7 | lh.superiortemporal_15 |
| 1012 | lh.superiortemporal_15 | rh.middletemporal_1 | rh.medialorbitofrontal_7 | rh.middletemporal_7 | rh.superiortemporal_4 | lh.superiortemporal_15 | rh.superiortemporal_3 |
| 1013 | rh.superiortemporal_4 | rh.superiortemporal_4 | rh.superiortemporal_4 | rh.middletemporal_1 | lh.superiortemporal_15 | lh.superiortemporal_12 | rh.lateralorbitofrontal_7 |
| 1014 | rh.superiortemporal_3 | rh.superiortemporal_3 | rh.superiortemporal_3 | rh.superiortemporal_3 | rh.superiortemporal_3 | rh.superiortemporal_3 | lh.superiortemporal_12 |
| 1015 | lh.superiortemporal_1 | lh.superiortemporal_1 | lh.superiortemporal_1 | lh.superiortemporal_1 | lh.superiortemporal_1 | lh.superiortemporal_1 | lh.superiortemporal_1 |
