# Supplementary material for: Robust circuitry-based scores of structural importance of human brain areas
Source: PLoS One. 2024 Jan 17;19(1):e0292613. doi: 10.1371/journal.pone.0292613 (PMC10793925; doi:10.1371/journal.pone.0292613)
Supplement: S1 File — (PDF) [file pone.0292613.s002.pdf]

```python
import os


def linelist(filename):
    """Makes list of the lines of the txt"""

    with open(filename) as f:
        lines = f.readlines()
    return lines


def find_nth(haystack, needle, n):
    start = haystack.find(needle)
    while start >= 0 and n > 1:
        start = haystack.find(needle, start + len(needle))
        n -= 1
    return start


def norm(x, y):
    """Converts a pair of numbers to a decimal fraction"""

    numpair = str(x) + "." + str(y)

    return numpair


def separ(decimal):
    """Converts the decimal fraction back to pair of numbers"""

    pont = decimal.find(".")
    first = int(decimal[:pont])
    second = int(decimal[pont + 1:])

    nemdecimal = [first, second]
    return nemdecimal


def calculation(folder1):
    """Calculates the different properties of the vertices and then the 
edges"""

    i = 1
    j = 1
    el_howmanykinds = {}  # hányféle él van belőle összesen
    elc_sum1 = {}  # össz fiber count
    elc_sum = {}
    ell_sum1 = {}  # össz fiber length
    ell_sum = {}
    positions = {}  # csúcsok koordinátái
    vertexoccurs = {}
    fiberc_sum = {}
    fractional1 = {}
    fractional = {}

    while i < 1016:
        while j < 1016:
            if i < j:
                el_howmanykinds[norm(i, j)] = 0
            j = j + 1
        i = i + 1
```

```
        j = 1

    for parr in el_howmanykinds.keys():
        elc_sum1[parr] = 0
        ell_sum1[parr] = 0
        fractional1[parr] = 0

    i = 1
    while i < 1016:
        positions[i] = [0, 0, 0, 0]
        vertexoccurs[i] = 0
        fiberc_sum[i] = 0
        i = i + 1

    i = 0
    j = 0
    for file in os.listdir(folder1):
        filename = os.fsdecode(file)
        filehely = folder + "\\" + filename
        text = linelist(filehely)

        source = "source="
        node = "<node"

        for line in text:
            if node in line:
                temporary = int(line[line.find('"') + 1:line.rfind('"')])

                neededx = text[i + 1]
                neededy = text[i + 2]
                neededz = text[i + 3]

                if neededx[neededx.find(">") + 1:neededx.rfind("<")] !=
"nan":
                    xkoord = float(neededx[neededx.find(">") +
1:neededx.rfind("<")])
                    ykoord = float(neededy[neededy.find(">") +
1:neededy.rfind("<")])
                    zkoord = float(neededz[neededz.find(">") +
1:neededz.rfind("<")])

                    positions[temporary][0] = positions[temporary][0] +
xkoord
                    positions[temporary][1] = positions[temporary][1] +
ykoord
                    positions[temporary][2] = positions[temporary][2] +
zkoord
                else:
                    positions[temporary][3] = positions[temporary][3] + 1
# nan-ok számolása

            if source in line:
                needed1 = text[i + 3]
                needed2 = text[i + 1]
                needed3 = text[i + 2]
                temporary1 = float(needed1[needed1.find(">") +
1:needed1.rfind("<")])
                temporary2 = float(needed2[needed2.find(">") +
1:needed2.rfind("<")])
                temporary3 = float(needed3[needed3.find(">") +
1:needed3.rfind("<")])
```

```
                first = int(line[find_nth(line, '"', 1) + 1:find_nth(line,
'"', 2)])
                second = int(line[find_nth(line, '"', 3) + 1:find_nth(line,
'"', 4)])
                numpair = norm(first, second)

                elc_sum1[numpair] = elc_sum1[numpair] + temporary1
                ell_sum1[numpair] = ell_sum1[numpair] + temporary2
                fractional1[numpair] = fractional1[numpair] + temporary3
                el_howmanykinds[numpair] = el_howmanykinds[numpair] + 1

            i = i + 1
        i = 0
        j = j + 1
        print(j)

    for elem in elc_sum1.keys():       # Kivenni azokat az éleket, amik nem
szerepeltek
        if elc_sum1[elem] != 0:
            elc_sum[elem] = elc_sum1[elem]
            ell_sum[elem] = ell_sum1[elem]
            fractional[elem] = fractional1[elem]

    for elem in elc_sum.keys():
        elc_sum[elem] = elc_sum[elem] / 1064
    for elem in ell_sum.keys():
        ell_sum[elem] = ell_sum[elem] / el_howmanykinds[elem]
    for elem in fractional.keys():
        fractional[elem] = fractional[elem] / 1064
    for elem in fiberc_sum.keys():
        fiberc_sum[elem] = fiberc_sum[elem] / 1064

    for elem in positions.values():
        elem[0] = elem[0] / (1064 - elem[3])
        elem[1] = elem[1] / (1064 - elem[3])
        elem[2] = elem[2] / (1064 - elem[3])
        del elem[3]

    for elem in elc_sum.keys():
        numpair = separ(elem)
        first = numpair[0]
        second = numpair[1]
        vertexoccurs[first] = vertexoccurs[first] + 1
        vertexoccurs[second] = vertexoccurs[second] + 1

        fiberc_sum[first] = fiberc_sum[first] + elc_sum[elem]
        fiberc_sum[second] = fiberc_sum[second] + elc_sum[elem]

    return [elc_sum, ell_sum, positions, vertexoccurs, fiberc_sum,
el_howmanykinds, fractional]


def name(lines, line):
    """Megtalálja a nameét az adott indexű agyterületnek."""

    needed = lines[line]
    namee = needed[needed.find(">") + 1:needed.rfind("<")]
    return namee


def parts(lines):
```

```python
    """Megtalálja a sorát az adott indexű agyterületnek."""

    areas = {}
    ident = 1
    for s in lines:
        if "d6" in s:
            if ident > 1:
                line = lines.index(s)
                part = name(lines, line)
                areas[ident - 1] = part
            ident = ident + 1
    return areas


def parts_others(lines):
    """A csúcsokhoz társítja pl. a csúcs agyterületének a nameét."""

    areas = {}
    i = 1
    while i < 1016:
        areas[i] = []
        i = i + 1
    ident4 = 1
    ident5 = 1
    ident6 = 1
    ident7 = 1
    for s in lines:
        if "d4" in s:
            if ident4 > 1:
                line = lines.index(s)
                part = name(lines, line)
                areas[ident4 - 1].append(part)
            ident4 = ident4 + 1

        if "d5" in s:
            if ident5 > 1:
                line = lines.index(s)
                part = name(lines, line)
                areas[ident5 - 1].append(part)
            ident5 = ident5 + 1

        if "d6" in s:
            if ident6 > 1:
                line = lines.index(s)
                part = name(lines, line)
                areas[ident6 - 1].append(part)
            ident6 = ident6 + 1

        if "d7" in s:
            if ident7 > 1:
                line = lines.index(s)
                part = name(lines, line)
                areas[ident7 - 1].append(part)
            ident7 = ident7 + 1
    return areas


testfile = "Gráfok 1064 emberre\\100206_repeated10_scale500.graphml"
folder = "Gráfok 1064 emberre"
directory = os.fsencode(folder)
averaged_graf = "Általam írt txt-k\\Átlagolt gráf.txt"
```

```
el_howmanykindse = "Általam írt txt-k\\Él hányok.txt"
vertex_howmanykindse = "Általam írt txt-k\\Csúcs hányok.txt"

datas = linelist(testfile)
brain_areas = parts(datas)
brain_areas_others = parts_others(datas)

print("Areas:")
print(brain_areas)
print(brain_areas_others)
print()

how_many = calculation(folder)        # Kiszámolunk minden adatot
fiberc_averageok = how_many[0]
fiberl_averageok = how_many[1]
positions = how_many[2]
occurs = how_many[3]
fiberc_vertexok = how_many[4]
el_occurs = how_many[5]
anisotropies = how_many[6]

positions_x = []
positions_y = []
positions_z = []
for vertex in positions.values():     # Lista a csúcsok helyzeteiről
    positions_x.append(vertex[0])
    positions_y.append(vertex[1])
    positions_z.append(vertex[2])

with open(averaged_graf, "a") as filee:
    for vertex in positions.keys():
        filee.write(str('    <node id="' + str(vertex) + '">\n'))
        filee.write(str('        <data key="d0">' + str(positions_x[vertex -
1]) + '</data>\n'))
        filee.write(str('        <data key="d1">' + str(positions_y[vertex -
1]) + '</data>\n'))
        filee.write(str('        <data key="d2">' + str(positions_z[vertex -
1]) + '</data>\n'))
        filee.write(str('        <data key="d3">' + str(vertex) +
'</data>\n'))
        filee.write(str('        <data key="d4">' +
str(brain_areas_others[vertex][0]) + '</data>\n'))
        filee.write(str('        <data key="d5">' +
str(brain_areas_others[vertex][1]) + '</data>\n'))
        filee.write(str('        <data key="d6">' +
str(brain_areas_others[vertex][2]) + '</data>\n'))
        filee.write(str('        <data key="d7">' +
str(brain_areas_others[vertex][3]) + '</data>\n'))
        filee.write(str('    </node>\n'))

    for el in fiberc_averageok.keys():
        pair = separ(el)
        firsto = pair[0]
        secondd = pair[1]
        filee.write(str('    <edge source="' + str(firsto) + '" target="' +
str(secondd) + '">\n'))
        filee.write(str('        <data key="d9">' +
str(fiberl_averageok[el]) + '</data>\n'))
        filee.write(str('        <data key="d10">' + str(anisotropies[el]) +
'</data>\n'))
        filee.write(str('        <data key="d11">' +
```

```
str(fiberc_averageok[el]) + '</data>\n'))
        filee.write(str('    </edge>\n'))

with open(el_howmanykindse, "w") as filee:
    filee.write(str(el_occurs))

with open(vertex_howmanykindse, "w") as filee:
    filee.write(str(occurs))
```

```python
import os
import random


def linelist(filename):
    """Makes list of the lines of the txt"""

    with open(filename) as f:
        lines = f.readlines()
    return lines


def find_nth(haystack, needle, n):
    start = haystack.find(needle)
    while start >= 0 and n > 1:
        start = haystack.find(needle, start + len(needle))
        n -= 1
    return start


def norm(x, y):
    """Converts a pair of numbers to a decimal fraction"""

    numpair = str(x) + "." + str(y)

    return numpair


def separ(decimal):
    """Converts the decimal fraction back to pair of numbers"""

    pont = decimal.find(".")
    first = int(decimal[:pont])
    second = int(decimal[pont + 1:])

    nemdecimal = [first, second]
    return nemdecimal


def calculation(folder1):
    """Calculates the different properties of the vertices and then the
edges"""

    i = 1
    j = 1
    el_howmanykinds = {}  # hányféle él van belőle összesen
    elc_sum1 = {}  # össz fiber count
    elc_sum = {}
    ell_sum1 = {}  # össz fiber length
    ell_sum = {}
    positions = {}  # csúcsok koordinátái
    vertexoccurs = {}
    fiberc_sum = {}
    fractional1 = {}
    fractional = {}

    while i < 1016:
        while j < 1016:
            if i < j:
                el_howmanykinds[norm(i, j)] = 0
            j = j + 1
```

```
        i = i + 1
        j = 1

    for parr in el_howmanykinds.keys():
        elc_sum1[parr] = 0
        ell_sum1[parr] = 0
        fractional1[parr] = 0

    i = 1
    while i < 1016:
        positions[i] = [0, 0, 0, 0]
        vertexoccurs[i] = 0
        fiberc_sum[i] = 0
        i = i + 1

    chosen = random.sample(range(1064), 100)
    chosen_list = []
    for num in range(1064):
        if num in chosen:
            chosen_list.append(1)
        else:
            chosen_list.append(0)
    print("Random 100 elem:")
    print(chosen)
    print(chosen_list)

    i = 0
    j = 0
    for file in os.listdir(folder1):
        if chosen_list[j] == 1:
            filename = os.fsdecode(file)
            filehely = folder + "\\" + filename
            text = linelist(filehely)

            source = "source="
            node = "<node"

            for line in text:
                if node in line:
                    temporary = int(line[line.find('"') +
1:line.rfind('"')])

                    neededx = text[i + 1]
                    neededy = text[i + 2]
                    neededz = text[i + 3]

                    if neededx[neededx.find(">") + 1:neededx.rfind("<")] !=
"nan":
                        xkoord = float(neededx[neededx.find(">") +
1:neededx.rfind("<")])
                        ykoord = float(neededy[neededy.find(">") +
1:neededy.rfind("<")])
                        zkoord = float(neededz[neededz.find(">") +
1:neededz.rfind("<")])

                        positions[temporary][0] = positions[temporary][0] +
xkoord
                        positions[temporary][1] = positions[temporary][1] +
ykoord
                        positions[temporary][2] = positions[temporary][2] +
zkoord
```

```
                    else:
                        positions[temporary][3] = positions[temporary][3] +
1  # nan-ok számolása

                if source in line:
                    needed1 = text[i + 3]
                    needed2 = text[i + 1]
                    needed3 = text[i + 2]
                    temporary1 = float(needed1[needed1.find(">") +
1:needed1.rfind("<")])
                    temporary2 = float(needed2[needed2.find(">") +
1:needed2.rfind("<")])
                    temporary3 = float(needed3[needed3.find(">") +
1:needed3.rfind("<")])
                    first = int(line[find_nth(line, '"', 1) +
1:find_nth(line, '"', 2)])
                    second = int(line[find_nth(line, '"', 3) +
1:find_nth(line, '"', 4)])
                    numpair = norm(first, second)

                    elc_sum1[numpair] = elc_sum1[numpair] + temporary1
                    ell_sum1[numpair] = ell_sum1[numpair] + temporary2
                    fractional1[numpair] = fractional1[numpair] +
temporary3
                    el_howmanykinds[numpair] = el_howmanykinds[numpair] + 1

                i = i + 1
            i = 0
        j = j + 1
        print(j)

    for elem in elc_sum1.keys():       # Kivenni azokat az éleket, amik nem
szerepeltek
        if elc_sum1[elem] != 0:
            elc_sum[elem] = elc_sum1[elem]
            ell_sum[elem] = ell_sum1[elem]
            fractional[elem] = fractional1[elem]

    for elem in elc_sum.keys():
        elc_sum[elem] = elc_sum[elem] / 100
    for elem in ell_sum.keys():
        ell_sum[elem] = ell_sum[elem] / el_howmanykinds[elem]
    for elem in fractional.keys():
        fractional[elem] = fractional[elem] / 100
    for elem in fiberc_sum.keys():
        fiberc_sum[elem] = fiberc_sum[elem] / 100

    for elem in positions.values():
        elem[0] = elem[0] / (100 - elem[3])
        elem[1] = elem[1] / (100 - elem[3])
        elem[2] = elem[2] / (100 - elem[3])
        del elem[3]

    for elem in elc_sum.keys():
        numpair = separ(elem)
        first = numpair[0]
        second = numpair[1]
        vertexoccurs[first] = vertexoccurs[first] + 1
        vertexoccurs[second] = vertexoccurs[second] + 1

        fiberc_sum[first] = fiberc_sum[first] + elc_sum[elem]
```

```python
        fiberc_sum[second] = fiberc_sum[second] + elc_sum[elem]

    return [elc_sum, ell_sum, positions, vertexoccurs, fiberc_sum,
el_howmanykinds, fractional]


def name(lines, line):
    """Megtalálja a nameét az adott indexű agyterületnek."""

    needed = lines[line]
    namee = needed[needed.find(">") + 1:needed.rfind("<")]
    return namee


def parts(lines):
    """Megtalálja a sorát az adott indexű agyterületnek."""

    areas = {}
    ident = 1
    for s in lines:
        if "d6" in s:
            if ident > 1:
                line = lines.index(s)
                part = name(lines, line)
                areas[ident - 1] = part
            ident = ident + 1
    return areas


def parts_others(lines):
    """A csúcsokhoz társítja pl. a csúcs agyterületének a nameét."""

    areas = {}
    i = 1
    while i < 1016:
        areas[i] = []
        i = i + 1
    ident4 = 1
    ident5 = 1
    ident6 = 1
    ident7 = 1
    for s in lines:
        if "d4" in s:
            if ident4 > 1:
                line = lines.index(s)
                part = name(lines, line)
                areas[ident4 - 1].append(part)
            ident4 = ident4 + 1

        if "d5" in s:
            if ident5 > 1:
                line = lines.index(s)
                part = name(lines, line)
                areas[ident5 - 1].append(part)
            ident5 = ident5 + 1

        if "d6" in s:
            if ident6 > 1:
                line = lines.index(s)
                part = name(lines, line)
                areas[ident6 - 1].append(part)
```

```
            ident6 = ident6 + 1

        if "d7" in s:
            if ident7 > 1:
                line = lines.index(s)
                part = name(lines, line)
                areas[ident7 - 1].append(part)
            ident7 = ident7 + 1
    return areas


testfile = "Gráfok 1064 emberre\\100206_repeated10_scale500.graphml"
folder = "Gráfok 1064 emberre"
directory = os.fsencode(folder)
rand_averaged_graf = "Általam írt txt-k\\Random átlagolt gráf.txt"
el_howmanykindse = "Általam írt txt-k\\Él hányok.txt"
vertex_howmanykindse = "Általam írt txt-k\\Csúcs hányok.txt"

datas = linelist(testfile)
brain_areas = parts(datas)
brain_areas_others = parts_others(datas)

print("Areas:")
print(brain_areas)
print(brain_areas_others)
print()

how_many = calculation(folder)       # Kiszámolunk minden adatot
fiberc_averageok = how_many[0]
fiberl_averageok = how_many[1]
positions = how_many[2]
occurs = how_many[3]
fiberc_vertexok = how_many[4]
el_occurs = how_many[5]
anisotropies = how_many[6]

positions_x = []
positions_y = []
positions_z = []
for vertex in positions.values():    # Lista a csúcsok helyzeteiről
    positions_x.append(vertex[0])
    positions_y.append(vertex[1])
    positions_z.append(vertex[2])

with open(rand_averaged_graf, "a") as filee:
    for vertex in positions.keys():
        filee.write(str('   <node id="' + str(vertex) + '">\n'))
        filee.write(str('       <data key="d0">' + str(positions_x[vertex -
1]) + '</data>\n'))
        filee.write(str('       <data key="d1">' + str(positions_y[vertex -
1]) + '</data>\n'))
        filee.write(str('       <data key="d2">' + str(positions_z[vertex -
1]) + '</data>\n'))
        filee.write(str('       <data key="d3">' + str(vertex) +
'</data>\n'))
        filee.write(str('       <data key="d4">' +
str(brain_areas_others[vertex][0]) + '</data>\n'))
        filee.write(str('       <data key="d5">' +
str(brain_areas_others[vertex][1]) + '</data>\n'))
        filee.write(str('       <data key="d6">' +
str(brain_areas_others[vertex][2]) + '</data>\n'))
```

```
        filee.write(str('        <data key="d7">' +
str(brain_areas_others[vertex][3]) + '</data>\n'))
        filee.write(str('    </node>\n'))

    for el in fiberc_averageok.keys():
        pair = separ(el)
        firsto = pair[0]
        secondd = pair[1]
        filee.write(str('    <edge source="' + str(firsto) + '" target="' +
str(secondd) + '">\n'))
        filee.write(str('        <data key="d9">' +
str(fiberl_averageok[el]) + '</data>\n'))
        filee.write(str('        <data key="d10">' + str(anisotropies[el]) +
'</data>\n'))
        filee.write(str('        <data key="d11">' +
str(fiberc_averageok[el]) + '</data>\n'))
        filee.write(str('    </edge>\n'))
```

```python
import matplotlib.pyplot as plt
from scipy.stats import spearmanr
from scipy.stats import kendalltau
import pandas as pd
import xlsxwriter


def linelist(filename):
    """Makes list of the lines of the txt"""

    with open(filename) as f:
        lines = f.readlines()
    return lines


def find_nth(haystack, needle, n):
    """Adott szövegből az n-edik helyét keresi meg."""
    start = haystack.find(needle)
    while start >= 0 and n > 1:
        start = haystack.find(needle, start+len(needle))
        n -= 1
    return start


def calculation(file):
    """Megszámolja a különböző csúcsok tulajdonságait."""

    howmanykinds = {}       # hányféle él van belőle összesen
    fiberc_sum = {}    # össz fiber count
    fiberc_max = {}     # legnagyobb fiber count
    fiberc_atl = {}
    fiberl_sum = {}    # össz fiber length
    fiberl_max = {}     # legnagyobb fiber length
    fiberl_atl = {}
    positions = {}         # vertexok koordinátái

    i = 1
    while i < 1016:
        howmanykinds[i] = 0
        fiberc_sum[i] = 0
        fiberc_max[i] = 0
        fiberc_atl[i] = 0
        fiberl_sum[i] = 0
        fiberl_max[i] = 0
        fiberl_atl[i] = 0
        positions[i] = [0, 0, 0, 0]
        i = i + 1

    i = 0

    text = linelist(file)

    source = "source="
    node = "<node"

    for line in text:
        if node in line:
            temporary = int(line[line.find('"')+1:line.rfind('"')])

            neededx = text[i + 1]
            neededy = text[i + 2]
```

```
            neededz = text[i + 3]

            if neededx[neededx.find(">")+1:neededx.rfind("<")] != "nan":
                xkoord =
float(neededx[neededx.find(">")+1:neededx.rfind("<")])
                ykoord =
float(neededy[neededy.find(">")+1:neededy.rfind("<")])
                zkoord =
float(neededz[neededz.find(">")+1:neededz.rfind("<")])

                positions[temporary][0] = positions[temporary][0] + xkoord
                positions[temporary][1] = positions[temporary][1] + ykoord
                positions[temporary][2] = positions[temporary][2] + zkoord
            else:
                positions[temporary][3] = positions[temporary][3] + 1

        if source in line:
            needed1 = text[i + 3]
            needed2 = text[i + 1]
            temporary1 =
float(needed1[needed1.find(">")+1:needed1.rfind("<")])
            temporary2 =
float(needed2[needed2.find(">")+1:needed2.rfind("<")])
            first = int(line[find_nth(line, '"', 1)+1:find_nth(line, '"',
2)])
            second = int(line[find_nth(line, '"', 3)+1:find_nth(line, '"',
4)])

            if temporary1 > fiberc_max[first]:
                fiberc_max[first] = temporary1
            fiberc_sum[first] = fiberc_sum[first] + temporary1
            if temporary1 > fiberc_max[second]:
                fiberc_max[second] = temporary1
            fiberc_sum[second] = fiberc_sum[second] + temporary1

            if temporary2 > fiberl_max[first]:
                fiberl_max[first] = temporary2
            fiberl_sum[first] = fiberl_sum[first] + temporary2
            if temporary2 > fiberl_max[second]:
                fiberl_max[second] = temporary2
            fiberl_sum[second] = fiberl_sum[second] + temporary2

            howmanykinds[first] = howmanykinds[first] + 1
            howmanykinds[second] = howmanykinds[second] + 1

        i = i + 1

    for elem in fiberc_sum.keys():
        if howmanykinds[elem] != 0:
            fiberc_atl[elem] = fiberc_sum[elem] / howmanykinds[elem]
            fiberl_atl[elem] = fiberl_sum[elem] / howmanykinds[elem]
        else:
            fiberc_atl[elem] = 0
            fiberl_atl[elem] = 0

    menny = [fiberc_sum, fiberc_max, fiberc_atl, fiberl_sum, fiberl_max,
fiberl_atl, positions, howmanykinds]
    return menny


def name(lines, line):
```

```python
    """Megtalálja a nameét az adott indexű agyterületnek."""

    needed = lines[line]
    namee = needed[needed.find(">")+1:needed.rfind("<")]
    return namee


def parts(lines):
    """Megtalálja a sorát az adott indexű agyterületnek."""

    areas = {}
    ident = 1
    for s in lines:
        if "d6" in s:
            if ident > 1:
                line = lines.index(s)
                part = name(lines, line)
                areas[ident - 1] = part
            ident = ident + 1
    return areas


def toorder(ord):
    """A nameeket orderbe renezi."""

    order = []
    for element in ord:
        order.append(element[0])
    return order


def itoorder(areas, order):
    """Az indexeket orderbe ordezi."""

    iorder = []
    for element in order:
        ind = list(areas.values()).index(element)
        iorder.append(ind)
    return iorder


def inversionnumber(iorder1, iorder2):
    """Két indextoorder kiszámolja a köztük levő inverziószámot."""
    inv = 0
    inver = []
    temporary = 0
    for elem1 in iorder1:
        i_hely1 = iorder1.index(elem1)
        i_hely2 = iorder2.index(elem1)
        for elem2 in iorder1:
            j_hely1 = iorder1.index(elem2)
            j_hely2 = iorder2.index(elem2)
            if (i_hely1 > j_hely1 and i_hely2 < j_hely2) or (i_hely1 <
j_hely1 and i_hely2 > j_hely2):
                inv = inv + 1
                temporary = temporary + 1
        inver.append(temporary)
        temporary = 0
    inv = inv / 2
    inver.insert(0, inv)
    return inver
```

```python
def distance(iorder1, iorder2, n):
    tav = 0
    tavok = []
    for elem in iorder1:
        tav = tav + abs(iorder1.index(elem) - iorder2.index(elem))
        tavok.append(abs(iorder1.index(elem) - iorder2.index(elem)))
    tav = tav / n
    tavok.insert(0, tav)
    return tavok


def mod_expdist(n, k):
    s = 1
    mod_dist = 0
    while s <= n:
        mod_dist = mod_dist + abs(k - s)
        s = s + 1
    mod_dist = mod_dist / n
    return mod_dist


def until(halmaz, vart):
    till_end = []
    till = 0
    x = 0
    for elem in halmaz:
        if elem > vart[x]:
            till = till + 1
        till_end.append(till)
        x = x + 1
    return till_end


print()
print("Gráf.")
print()

averaged_graf = "Általam írt txt-k\\Átlagolt gráf.txt"
mod_averaged_graf = "Általam írt txt-k\\Módosított átlagolt gráf.txt"
rand_averaged_graf = "Általam írt txt-k\\Random átlagolt gráf.txt"
vertex_occursk = "Általam írt txt-k\\Csúcs hányok.txt"
rand_graf = "Általam írt txt-k\\Random gráf.txt"

numbers = []
a = 1015
while 0 < a:
    numbers.append(a)
    a = a - 1
numbers.reverse()

halfs = []
for number in numbers:
    halfs.append(number / 2)

fel = []
for number in numbers:
    fel.append(1015 / 2)

iorder_max_haanyok = []
```

```
invset_hco = []
invset_hcm = []
invset_hca = []
invset_hlo = []
invset_hlm = []
invset_hla = []
invset_hh = []
invset_mhca = []
invset_mhla = []

invset_oo = []
invset_om = []
invset_oa = []
invset_mo = []
invset_mm = []
invset_ma = []
invset_ao = []
invset_am = []
invset_aa = []

distset_hco = []
distset_hcm = []
distset_hca = []
distset_hlo = []
distset_hlm = []
distset_hla = []

distset_oo = []
distset_om = []
distset_oa = []
distset_mo = []
distset_mm = []
distset_ma = []
distset_ao = []
distset_am = []
distset_aa = []

datas = linelist(averaged_graf)
brain_areas = parts(datas)
print("Areas:")
print(brain_areas)
print()

how_many = calculation(rand_averaged_graf)   # Kiszámolunk minden adatot

fiberc_sums = how_many[0]          # Kivesszük külön a tulajdonságokat
fiberc_maxok = how_many[1]
fiberc_averageok = how_many[2]
fiberl_sums = how_many[3]
fiberl_maxok = how_many[4]
fiberl_averageok = how_many[5]
helyzetek = how_many[6]
foknumbers = how_many[7]       # Ez megegyezik a bekért vertex_occurs-kal

# vertex_occurs bekérése külső file-ból - fokszámok
datas = linelist(vertex_occursk)
vertex_occurs = {}
for linee in datas:     # 1 sora van
    vertex_haanyok = linee.split(", ")
    vertex_haanyok[0] = vertex_haanyok[0][1:]
```

```
    vertex_haanyok[-1] = vertex_haanyok[-1][:-2]
    for elementt in vertex_haanyok:
        disjoint = elementt.split(": ")
        disjoint[0] = int(disjoint[0])
        disjoint[1] = float(disjoint[1])
        vertex_occurs[disjoint[0]] = disjoint[1]

print("Helyzetek:")
print(helyzetek)
print()

print("Alapdatas a how_many függvényből:")
print(vertex_occurs)
print()
print(fiberc_sums)
print(fiberc_maxok)
print(fiberc_averageok)
print()
print(fiberl_sums)
print(fiberl_maxok)
print(fiberl_averageok)
print()

ord_occurs = sorted(vertex_occurs.items(), key=lambda item: item[1], reverse=True)       # Csökkenőbe ordezzük
ord_fiberc_sums = sorted(fiberc_sums.items(), key=lambda item: item[1], reverse=True)
ord_fiberc_maxok = sorted(fiberc_maxok.items(), key=lambda item: item[1], reverse=True)
ord_fiberc_averageok = sorted(fiberc_averageok.items(), key=lambda item: item[1], reverse=True)
ord_fiberl_sums = sorted(fiberl_sums.items(), key=lambda item: item[1], reverse=True)
ord_fiberl_maxok = sorted(fiberl_maxok.items(), key=lambda item: item[1], reverse=True)
ord_fiberl_averageok = sorted(fiberl_averageok.items(), key=lambda item: item[1], reverse=True)

print("ordezett alapdatas:")
print(ord_occurs)
print()
print(ord_fiberc_sums)
print(ord_fiberc_maxok)
print(ord_fiberc_averageok)
print()
print(ord_fiberl_sums)
print(ord_fiberl_maxok)
print(ord_fiberl_averageok)
print()

iorder_occurs = toorder(ord_occurs)        # orderbe ordezük, indexet kiírva
iorder_fiberc_sums = toorder(ord_fiberc_sums)
iorder_fiberc_maxok = toorder(ord_fiberc_maxok)
iorder_fiberc_averageok = toorder(ord_fiberc_averageok)
iorder_fiberl_sums = toorder(ord_fiberl_sums)
iorder_fiberl_maxok = toorder(ord_fiberl_maxok)
iorder_fiberl_averageok = toorder(ord_fiberl_averageok)

fileheely = "Általam írt txt-k\\El adatok.txt"
datas = linelist(fileheely)
```

```
for linee in datas:
    if datas.index(linee) == 0:
        iorder_max_haanyok = linee.split(", ")
        iorder_max_haanyok[0] = iorder_max_haanyok[0][1:]
        iorder_max_haanyok[-1] = iorder_max_haanyok[-1][:-2]
iorder_max_occurs = list(map(int, iorder_max_haanyok))

"""
file_object = open("Összeredmény.txt", "w")
file_object.write("Hány élben szerepel:\n" + str(iorder_occurs) + "\n")
file_object = open("Összeredmény.txt", "a")
file_object.write("Fiber count datas:\n")
file_object.write(str(iorder_fiberc_sums) + "\n")
file_object.write(str(iorder_fiberc_maxok) + "\n")
file_object.write(str(iorder_fiberc_averageok) + "\n")
file_object.write("Fiber length datas:\n")
file_object.write(str(iorder_fiberl_sums) + "\n")
file_object.write(str(iorder_fiberl_maxok) + "\n")
file_object.write(str(iorder_fiberl_averageok) + "\n")
"""

print("Alapdatas iorderjei:")
print(iorder_occurs)
print()
print(iorder_fiberc_sums)
print(iorder_fiberc_maxok)
print(iorder_fiberc_averageok)
print()
print(iorder_fiberl_sums)
print(iorder_fiberl_maxok)
print(iorder_fiberl_averageok)
print()
print(iorder_max_occurs)
print()

degree_nameek = []
fiberc_sums_nameek = []
fiberc_maxok_nameek = []
fiberc_averageok_nameek = []
fiberl_sums_nameek = []
fiberl_maxok_nameek = []
fiberl_averageok_nameek = []

for elementt in iorder_occurs:
    degree_nameek.append(brain_areas[elementt])
for elementt in iorder_fiberc_sums:
    fiberc_sums_nameek.append(brain_areas[elementt])
for elementt in iorder_fiberc_maxok:
    fiberc_maxok_nameek.append(brain_areas[elementt])
for elementt in iorder_fiberc_averageok:
    fiberc_averageok_nameek.append(brain_areas[elementt])
for elementt in iorder_fiberl_sums:
    fiberl_sums_nameek.append(brain_areas[elementt])
for elementt in iorder_fiberl_maxok:
    fiberl_maxok_nameek.append(brain_areas[elementt])
for elementt in iorder_fiberl_averageok:
    fiberl_averageok_nameek.append(brain_areas[elementt])

# Cikkhez excel készítése
workbook = xlsxwriter.Workbook('Vertices.xlsx')
worksheet = workbook.add_worksheet()
```

```
for number in numbers:
    worksheet.write((number - 1), 0, degree_nameek[number - 1])
for number in numbers:
    worksheet.write((number - 1), 1, fiberc_sums_nameek[number - 1])
for number in numbers:
    worksheet.write((number - 1), 2, fiberc_maxok_nameek[number - 1])
for number in numbers:
    worksheet.write((number - 1), 3, fiberc_averageok_nameek[number - 1])
for number in numbers:
    worksheet.write((number - 1), 4, fiberl_sums_nameek[number - 1])
for number in numbers:
    worksheet.write((number - 1), 5, fiberl_maxok_nameek[number - 1])
for number in numbers:
    worksheet.write((number - 1), 6, fiberl_averageok_nameek[number - 1])
workbook.close()

hely_sums = {}
for vertexx in iorder_occurs:
    hely_sums[vertexx] = iorder_occurs.index(vertexx)
    hely_sums[vertexx] = hely_sums[vertexx] +
iorder_fiberc_sums.index(vertexx)
    hely_sums[vertexx] = hely_sums[vertexx] +
iorder_fiberc_maxok.index(vertexx)
    hely_sums[vertexx] = hely_sums[vertexx] +
iorder_fiberc_averageok.index(vertexx)
print("Helyzet összegek:")
print(str(hely_sums))
print()

# Spearman elemzések
df_hco = pd.DataFrame({'vertex_occurs': vertex_occurs, 'fiberc_sums':
fiberc_sums})
df_hcm = pd.DataFrame({'vertex_occurs': vertex_occurs, 'fiberc_maxok':
fiberc_maxok})
df_hca = pd.DataFrame({'vertex_occurs': vertex_occurs, 'fiberc_averageok':
fiberc_averageok})
df_hlo = pd.DataFrame({'vertex_occurs': vertex_occurs, 'fiberl_sums':
fiberl_sums})
df_hlm = pd.DataFrame({'vertex_occurs': vertex_occurs, 'fiberl_maxok':
fiberl_maxok})
df_hla = pd.DataFrame({'vertex_occurs': vertex_occurs, 'fiberl_averageok':
fiberl_averageok})
coeff_hco, p_hco = spearmanr(df_hco['vertex_occurs'],
df_hco['fiberc_sums'])
coeff_hcm, p_hcm = spearmanr(df_hcm['vertex_occurs'],
df_hcm['fiberc_maxok'])
coeff_hca, p_hca = spearmanr(df_hca['vertex_occurs'],
df_hca['fiberc_averageok'])
coeff_hlo, p_hlo = spearmanr(df_hlo['vertex_occurs'],
df_hlo['fiberl_sums'])
coeff_hlm, p_hlm = spearmanr(df_hlm['vertex_occurs'],
df_hlm['fiberl_maxok'])
coeff_hla, p_hla = spearmanr(df_hla['vertex_occurs'],
df_hla['fiberl_averageok'])

tau_hco, pt_hco = kendalltau(list(vertex_occurs.values()),
list(fiberc_sums.values()))
tau_hcm, pt_hcm = kendalltau(list(vertex_occurs.values()),
list(fiberc_maxok.values()))
tau_hca, pt_hca = kendalltau(list(vertex_occurs.values()),
list(fiberc_averageok.values()))
```

```
tau_hlo, pt_hlo = kendalltau(list(vertex_occurs.values()),
list(fiberl_sums.values()))
tau_hlm, pt_hlm = kendalltau(list(vertex_occurs.values()),
list(fiberl_maxok.values()))
tau_hla, pt_hla = kendalltau(list(vertex_occurs.values()),
list(fiberl_averageok.values()))

print("Amikre mennek az együtthatók:")
print(vertex_occurs)
print(fiberc_averageok)
print(list(vertex_occurs.values()))
print(df_hca)
print()

print("Spearman Coefficient-ek és p-value-k occurs-kal:")
print(coeff_hco, p_hco, " és ", tau_hco, pt_hco)
print(coeff_hcm, p_hcm, " és ", tau_hcm, pt_hcm)
print(coeff_hca, p_hca, " és ", tau_hca, pt_hca)
print()
print(coeff_hlo, p_hlo, " és ", tau_hlo, pt_hlo)
print(coeff_hlm, p_hlm, " és ", tau_hlm, pt_hlm)
print(coeff_hla, p_hla, " és ", tau_hla, pt_hla)
print()

df_oo = pd.DataFrame({'fiberc_sums': fiberc_sums, 'fiberl_sums':
fiberl_sums})
df_om = pd.DataFrame({'fiberc_sums': fiberc_sums, 'fiberl_maxok':
fiberl_maxok})
df_oa = pd.DataFrame({'fiberc_sums': fiberc_sums, 'fiberl_averageok':
fiberl_averageok})
df_mo = pd.DataFrame({'fiberc_maxok': fiberc_maxok, 'fiberl_sums':
fiberl_sums})
df_mm = pd.DataFrame({'fiberc_maxok': fiberc_maxok, 'fiberl_maxok':
fiberl_maxok})
df_ma = pd.DataFrame({'fiberc_maxok': fiberc_maxok, 'fiberl_averageok':
fiberl_averageok})
df_ao = pd.DataFrame({'fiberc_averageok': fiberc_averageok, 'fiberl_sums':
fiberl_sums})
df_am = pd.DataFrame({'fiberc_averageok': fiberc_averageok, 'fiberl_maxok':
fiberl_maxok})
df_aa = pd.DataFrame({'fiberc_averageok': fiberc_averageok,
'fiberl_averageok': fiberl_averageok})
coeff_oo, p_oo = spearmanr(df_oo['fiberc_sums'], df_oo['fiberl_sums'])
coeff_om, p_om = spearmanr(df_om['fiberc_sums'], df_om['fiberl_maxok'])
coeff_oa, p_oa = spearmanr(df_oa['fiberc_sums'], df_oa['fiberl_averageok'])
coeff_mo, p_mo = spearmanr(df_mo['fiberc_maxok'], df_mo['fiberl_sums'])
coeff_mm, p_mm = spearmanr(df_mm['fiberc_maxok'], df_mm['fiberl_maxok'])
coeff_ma, p_ma = spearmanr(df_ma['fiberc_maxok'],
df_ma['fiberl_averageok'])
coeff_ao, p_ao = spearmanr(df_ao['fiberc_averageok'], df_ao['fiberl_sums'])
coeff_am, p_am = spearmanr(df_am['fiberc_averageok'],
df_am['fiberl_maxok'])
coeff_aa, p_aa = spearmanr(df_aa['fiberc_averageok'],
df_aa['fiberl_averageok'])

tau_oo, pt_oo = kendalltau(list(fiberc_sums.values()),
list(fiberl_sums.values()))
tau_om, pt_om = kendalltau(list(fiberc_sums.values()),
list(fiberl_maxok.values()))
tau_oa, pt_oa = kendalltau(list(fiberc_sums.values()),
list(fiberl_averageok.values()))
```

```
tau_mo, pt_mo = kendalltau(list(fiberc_maxok.values()),
list(fiberl_sums.values()))
tau_mm, pt_mm = kendalltau(list(fiberc_maxok.values()),
list(fiberl_maxok.values()))
tau_ma, pt_ma = kendalltau(list(fiberc_maxok.values()),
list(fiberl_averageok.values()))
tau_ao, pt_ao = kendalltau(list(fiberc_averageok.values()),
list(fiberl_sums.values()))
tau_am, pt_am = kendalltau(list(fiberc_averageok.values()),
list(fiberl_maxok.values()))
tau_aa, pt_aa = kendalltau(list(fiberc_averageok.values()),
list(fiberl_averageok.values()))

print("Spearman Coefficient-ek és p-valuek cl-lel:")
print(coeff_oo, p_oo, " és ", tau_oo, pt_oo)
print(coeff_om, p_om, " és ", tau_om, pt_om)
print(coeff_oa, p_oa, " és ", tau_oa, pt_oa)
print()
print(coeff_mo, p_mo, " és ", tau_mo, pt_mo)
print(coeff_mm, p_mm, " és ", tau_mm, pt_mm)
print(coeff_ma, p_ma, " és ", tau_ma, pt_ma)
print()
print(coeff_ao, p_ao, " és ", tau_ao, pt_ao)
print(coeff_am, p_am, " és ", tau_am, pt_am)
print(coeff_aa, p_aa, " és ", tau_aa, pt_aa)
print()

df_com = pd.DataFrame({'fiberc_sums': fiberc_sums, 'fiberc_maxok':
fiberc_maxok})
df_coa = pd.DataFrame({'fiberc_sums': fiberc_sums, 'fiberc_averageok':
fiberc_averageok})
df_cma = pd.DataFrame({'fiberc_maxok': fiberc_maxok, 'fiberc_averageok':
fiberc_averageok})
df_lom = pd.DataFrame({'fiberl_sums': fiberl_sums, 'fiberl_maxok':
fiberl_maxok})
df_loa = pd.DataFrame({'fiberl_sums': fiberl_sums, 'fiberl_averageok':
fiberl_averageok})
df_lma = pd.DataFrame({'fiberl_maxok': fiberl_maxok, 'fiberl_averageok':
fiberl_averageok})
coeff_com, p_com = spearmanr(df_com['fiberc_sums'], df_com['fiberc_maxok'])
coeff_coa, p_coa = spearmanr(df_coa['fiberc_sums'],
df_coa['fiberc_averageok'])
coeff_cma, p_cma = spearmanr(df_cma['fiberc_maxok'],
df_cma['fiberc_averageok'])
coeff_lom, p_lom = spearmanr(df_lom['fiberl_sums'], df_lom['fiberl_maxok'])
coeff_loa, p_loa = spearmanr(df_loa['fiberl_sums'],
df_loa['fiberl_averageok'])
coeff_lma, p_lma = spearmanr(df_lma['fiberl_maxok'],
df_lma['fiberl_averageok'])

tau_com, pt_com = kendalltau(list(fiberc_sums.values()),
list(fiberc_maxok.values()))
tau_coa, pt_coa = kendalltau(list(fiberc_sums.values()),
list(fiberc_averageok.values()))
tau_cma, pt_cma = kendalltau(list(fiberc_maxok.values()),
list(fiberc_averageok.values()))
tau_lom, pt_lom = kendalltau(list(fiberl_sums.values()),
list(fiberl_maxok.values()))
tau_loa, pt_loa = kendalltau(list(fiberl_sums.values()),
list(fiberl_averageok.values()))
tau_lma, pt_lma = kendalltau(list(fiberl_maxok.values()),
```

```
list(fiberl_averageok.values()))

print("Spearman Coefficient-ek súlyok saját magukkal és hsumok saját 
magukkal:")
print(coeff_com, p_com, " és ", tau_com, pt_com)
print(coeff_coa, p_coa, " és ", tau_coa, pt_coa)
print(coeff_cma, p_cma, " és ", tau_cma, pt_cma)
print()
print(coeff_lom, p_lom, " és ", tau_lom, pt_lom)
print(coeff_loa, p_loa, " és ", tau_loa, pt_loa)
print(coeff_lma, p_lma, " és ", tau_lma, pt_lma)
print()

# Példa a randomra
numbers_dict = {}
for number in numbers:
    numbers_dict[number] = number
df_random = pd.DataFrame({'fiberc_averageok': fiberc_averageok, 
'numbers_dict': numbers_dict})
coeff_random, p_random = spearmanr(df_random['fiberc_averageok'], 
df_random['numbers_dict'])
print("Spearman Coefficient-ek ami random:")
print(coeff_random, p_random)
print()

fiberc_indexek = []
fiberl_indexek = []
for elementt in numbers:
    fiberc_indexek.append(iorder_fiberc_maxok.index(elementt))
    fiberl_indexek.append(iorder_fiberl_averageok.index(elementt))

fiberc_atl_order = []
fiberl_atl_order = []
for elementt in iorder_fiberc_averageok:
    fiberc_atl_order.append(fiberc_averageok[elementt])
    fiberl_atl_order.append(fiberl_averageok[elementt])

# plt.plot(fiberc_indexek, fiberl_indexek, "s", markersize=0.3, color="r")
plt.plot(fiberc_atl_order, fiberl_atl_order, "s", markersize=0.3, 
color="r")
plt.xlim([0, 2])
plt.show()

inver_hco = inversionnumber(iorder_occurs, iorder_fiberc_sums)       # 
Kiszámoljuk az inverziószámokat
inver_hcm = inversionnumber(iorder_occurs, iorder_fiberc_maxok)
inver_hca = inversionnumber(iorder_occurs, iorder_fiberc_averageok)
inver_hlo = inversionnumber(iorder_occurs, iorder_fiberl_sums)
inver_hlm = inversionnumber(iorder_occurs, iorder_fiberl_maxok)
inver_hla = inversionnumber(iorder_occurs, iorder_fiberl_averageok)
inver_hh = inversionnumber(iorder_max_occurs, iorder_occurs)
inver_mhca = inversionnumber(iorder_max_occurs, iorder_fiberc_averageok)
inver_mhla = inversionnumber(iorder_max_occurs, iorder_fiberl_averageok)

print("Inver-ek occurs-kal:")
print(inver_hco)
print(inver_hcm)
print(inver_hca)
print()
print(inver_hlo)
print(inver_hlm)
```

```
print(inver_hla)
print()
print(inver_hh)
print()
print(inver_mhca)
print(inver_mhla)
print()

for ele in inver_hco:
    invset_hco.append(ele)
for ele in inver_hcm:
    invset_hcm.append(ele)
for ele in inver_hca:
    invset_hca.append(ele)
for ele in inver_hlo:
    invset_hlo.append(ele)
for ele in inver_hlm:
    invset_hlm.append(ele)
for ele in inver_hla:
    invset_hla.append(ele)
for ele in inver_hh:
    invset_hh.append(ele)
for ele in inver_mhca:
    invset_mhca.append(ele)
for ele in inver_mhla:
    invset_mhla.append(ele)
del invset_hco[0]
del invset_hcm[0]
del invset_hca[0]
del invset_hlo[0]
del invset_hlm[0]
del invset_hla[0]
del invset_hh[0]
del invset_mhca[0]
del invset_mhla[0]

invuntil_hco = until(invset_hco, fel)
invuntil_hcm = until(invset_hcm, fel)
invuntil_hca = until(invset_hca, fel)
invuntil_hlo = until(invset_hlo, fel)
invuntil_hlm = until(invset_hlm, fel)
invuntil_hla = until(invset_hla, fel)
invuntil_hh = until(invset_hh, fel)
invuntil_mhca = until(invset_mhca, fel)
invuntil_mhla = until(invset_mhla, fel)

"""
tavol_hco = distance(iorder_occurs, iorder_fiberc_sums, 1015)        #
Kiszámoljuk a távolságokat
tavol_hcm = distance(iorder_occurs, iorder_fiberc_maxok, 1015)
tavol_hca = distance(iorder_occurs, iorder_fiberc_averageok, 1015)
tavol_hlo = distance(iorder_occurs, iorder_fiberl_sums, 1015)
tavol_hlm = distance(iorder_occurs, iorder_fiberl_maxok, 1015)
tavol_hla = distance(iorder_occurs, iorder_fiberl_averageok, 1015)

print("Tavol-ok occurs-kal:")
print(tavol_hco)
print(tavol_hcm)
print(tavol_hca)
print()
print(tavol_hlo)
```

```
print(tavol_hlm)
print(tavol_hla)
print()

for ele in tavol_hco:
    distset_hco.append(ele)
for ele in tavol_hcm:
    distset_hcm.append(ele)
for ele in tavol_hca:
    distset_hca.append(ele)
for ele in tavol_hlo:
    distset_hlo.append(ele)
for ele in tavol_hlm:
    distset_hlm.append(ele)
for ele in tavol_hla:
    distset_hla.append(ele)
del distset_hco[0]
del distset_hcm[0]
del distset_hca[0]
del distset_hlo[0]
del distset_hlm[0]
del distset_hla[0]
"""

inver_oo = inversionnumber(iorder_fiberl_sums, iorder_fiberc_sums)
inver_om = inversionnumber(iorder_fiberl_sums, iorder_fiberc_maxok)
inver_oa = inversionnumber(iorder_fiberl_sums, iorder_fiberc_averageok)
inver_mo = inversionnumber(iorder_fiberl_maxok, iorder_fiberc_sums)
inver_mm = inversionnumber(iorder_fiberl_maxok, iorder_fiberc_maxok)
inver_ma = inversionnumber(iorder_fiberl_maxok, iorder_fiberc_averageok)
inver_ao = inversionnumber(iorder_fiberl_averageok, iorder_fiberc_sums)
inver_am = inversionnumber(iorder_fiberl_averageok, iorder_fiberc_maxok)
inver_aa = inversionnumber(iorder_fiberl_averageok,
iorder_fiberc_averageok)

print("Inver-ek occurs nélkül:")
print(inver_oo)
print(inver_om)
print(inver_oa)
print()
print(inver_mo)
print(inver_mm)
print(inver_ma)
print()
print(inver_ao)
print(inver_am)
print(inver_aa)
print()

for ele in inver_oo:
    invset_oo.append(ele)
for ele in inver_om:
    invset_om.append(ele)
for ele in inver_oa:
    invset_oa.append(ele)
for ele in inver_mo:
    invset_mo.append(ele)
for ele in inver_mm:
    invset_mm.append(ele)
for ele in inver_ma:
    invset_ma.append(ele)
```

```
for ele in inver_ao:
    invset_ao.append(ele)
for ele in inver_am:
    invset_am.append(ele)
for ele in inver_aa:
    invset_aa.append(ele)
del invset_oo[0]
del invset_om[0]
del invset_oa[0]
del invset_mo[0]
del invset_mm[0]
del invset_ma[0]
del invset_ao[0]
del invset_am[0]
del invset_aa[0]

invuntil_oo = until(invset_oo, fel)
invuntil_om = until(invset_om, fel)
invuntil_oa = until(invset_oa, fel)
invuntil_mo = until(invset_mo, fel)
invuntil_mm = until(invset_mm, fel)
invuntil_ma = until(invset_ma, fel)
invuntil_ao = until(invset_ao, fel)
invuntil_am = until(invset_am, fel)
invuntil_aa = until(invset_aa, fel)

"""
tavol_oo = distance(iorder_fiberc_sums, iorder_fiberl_sums, 1015)
tavol_om = distance(iorder_fiberc_sums, iorder_fiberl_maxok, 1015)
tavol_oa = distance(iorder_fiberc_sums, iorder_fiberl_averageok, 1015)
tavol_mo = distance(iorder_fiberc_maxok, iorder_fiberl_sums, 1015)
tavol_mm = distance(iorder_fiberc_maxok, iorder_fiberl_maxok, 1015)
tavol_ma = distance(iorder_fiberc_maxok, iorder_fiberl_averageok, 1015)
tavol_ao = distance(iorder_fiberc_averageok, iorder_fiberl_sums, 1015)
tavol_am = distance(iorder_fiberc_averageok, iorder_fiberl_maxok, 1015)
tavol_aa = distance(iorder_fiberc_averageok, iorder_fiberl_averageok, 1015)

print("Tavol-ok occurs nélkül:")
print(tavol_oo)
print(tavol_om)
print(tavol_oa)
print()
print(tavol_mo)
print(tavol_mm)
print(tavol_ma)
print()
print(tavol_ao)
print(tavol_am)
print(tavol_aa)
print()

for ele in tavol_oo:
    distset_oo.append(ele)
for ele in tavol_om:
    distset_om.append(ele)
for ele in tavol_oa:
    distset_oa.append(ele)
for ele in tavol_mo:
    distset_mo.append(ele)
for ele in tavol_mm:
    distset_mm.append(ele)
```

```
for ele in tavol_ma:
    distset_ma.append(ele)
for ele in tavol_ao:
    distset_ao.append(ele)
for ele in tavol_am:
    distset_am.append(ele)
for ele in tavol_aa:
    distset_aa.append(ele)
del distset_oo[0]
del distset_om[0]
del distset_oa[0]
del distset_mo[0]
del distset_mm[0]
del distset_ma[0]
del distset_ao[0]
del distset_am[0]
del distset_aa[0]
"""

a1 = plt.subplot2grid((3, 2), (0, 0), colspan=1, rowspan=1)
a2 = plt.subplot2grid((3, 2), (0, 1), colspan=1, rowspan=1)
b1 = plt.subplot2grid((3, 2), (1, 0), colspan=1, rowspan=1)
b2 = plt.subplot2grid((3, 2), (1, 1), colspan=1, rowspan=1)
c1 = plt.subplot2grid((3, 2), (2, 0), colspan=1, rowspan=1)

a1.plot(numbers, invuntil_hco, color="r", label="Degree, SUM-weight")
a1.plot(numbers, invuntil_hcm, color="g", label="Degree, MAX-weight")
a1.plot(numbers, invuntil_hca, color="b", label="Degree, AVG-weight")
a1.plot(numbers, halfs, color="k")
a1.legend(loc="upper left")

a2.plot(numbers, invuntil_hlo, color="r", label="Degree, SUM-length")
a2.plot(numbers, invuntil_hlm, color="g", label="Degree, MAX-length")
a2.plot(numbers, invuntil_hla, color="b", label="Degree, AVG-length")
a2.plot(numbers, halfs, color="k")
a2.legend(loc="upper left")

"""
c2 = plt.subplot2grid((2, 3), (2, 1), colspan=1, rowspan=1)
c2.plot(numbers, invuntil_hh, color="r")
c2.plot(numbers, invuntil_mhca, color="g")
c2.plot(numbers, invuntil_mhla, color="b")
c2.plot(numbers, halfs, color="k")
"""

b1.plot(numbers, invuntil_oo, color="r", label="SUM-weight, SUM-length")
b1.plot(numbers, invuntil_om, color="g", label="SUM-weight, MAX-length")
b1.plot(numbers, invuntil_oa, color="b", label="SUM-weight, AVG-length")
b1.plot(numbers, halfs, color="k")
b1.legend(loc="upper left")

b2.plot(numbers, invuntil_mo, color="r", label="MAX-weight, SUM-length")
b2.plot(numbers, invuntil_mm, color="g", label="MAX-weight, MAX-length")
b2.plot(numbers, invuntil_ma, color="b", label="MAX-weight, AVG-length")
b2.plot(numbers, halfs, color="k")
b2.legend(loc="upper left")

c1.plot(numbers, invuntil_ao, color="r", label="AVG-weight, SUM-length")
c1.plot(numbers, invuntil_am, color="g", label="AVG-weight, MAX-length")
c1.plot(numbers, invuntil_aa, color="b", label="AVG-weight, AVG-length")
c1.plot(numbers, halfs, color="k")
```

```
c1.legend(loc="upper left")

plt.show()

"""
plt.plot(numbers, invuntil_mo, color="r", label="Súlymax., hsumössz.")
plt.plot(numbers, invuntil_mm, color="g", label="Súlymax., hsummax.")
plt.plot(numbers, invuntil_ma, color="b", label="Súlymax., hsumátl.")
plt.plot(numbers, halfs, color="k")
plt.tick_params(labelsize=15)
plt.show()
"""

plt.plot(numbers, invuntil_hco, color="r", label="Degree, SUM-weight")
plt.plot(numbers, invuntil_hcm, color="g", label="Degree, MAX-weight")
plt.plot(numbers, invuntil_hca, color="b", label="Degree, AVG-weight")
plt.plot(numbers, halfs, color="k")
plt.tick_params(labelsize=15)
plt.xlabel("Number of vertices in ordering")
plt.ylabel("Number of inversions")
plt.show()

plt.plot(numbers, invuntil_hlo, color="r", label="Degree, SUM-weight")
plt.plot(numbers, invuntil_hlm, color="g", label="Degree, MAX-weight")
plt.plot(numbers, invuntil_hla, color="b", label="Degree, AVG-weight")
plt.plot(numbers, halfs, color="k")
plt.tick_params(labelsize=15)
plt.xlabel("Number of vertices in ordering")
plt.ylabel("Number of inversions")
plt.show()

plt.plot(numbers, invuntil_oo, color="r", label="SUM-weight, SUM-length")
plt.plot(numbers, invuntil_om, color="g", label="SUM-weight, MAX-length")
plt.plot(numbers, invuntil_oa, color="b", label="SUM-weight, AVG-length")
plt.plot(numbers, halfs, color="k")
plt.tick_params(labelsize=15)
plt.xlabel("Number of vertices in ordering")
plt.ylabel("Number of inversions")
plt.show()

plt.plot(numbers, invuntil_mo, color="r", label="MAX-weight, SUM-length")
plt.plot(numbers, invuntil_mm, color="g", label="MAX-weight, MAX-length")
plt.plot(numbers, invuntil_ma, color="b", label="MAX-weight, AVG-length")
plt.plot(numbers, halfs, color="k")
plt.tick_params(labelsize=15)
plt.xlabel("Number of vertices in ordering")
plt.ylabel("Number of inversions")
plt.show()

plt.plot(numbers, invuntil_ao, color="r", label="AVG-weight, SUM-length")
plt.plot(numbers, invuntil_am, color="g", label="AVG-weight, MAX-length")
plt.plot(numbers, invuntil_aa, color="b", label="AVG-weight, AVG-length")
plt.plot(numbers, halfs, color="k")
plt.tick_params(labelsize=15)
plt.xlabel("Number of vertices in ordering")
plt.ylabel("Number of inversions")
plt.show()
```
